# SRP orchestrates protein biogenesis beyond initial ER membrane targeting

**DOI:** 10.1038/s41467-026-74404-2

**Published:** 2026-06-16

**Authors:** Ilgın Eser Kotan, Sabrina Sartori, Rudra Bose, Bernd Bukau, Günter Kramer

**Affiliations:** 1 Center for Molecular Biology of the University of Heidelberg (ZMBH), DKFZ-ZMBH Alliance, Heidelberg, Germany; 2 Present Address: Institute for Neurodegenerative Diseases, University of California, San Francisco, San Francisco, CA USA

**Keywords:** Ribosome, Membrane proteins, Endoplasmic reticulum, Protein translocation

## Abstract

The Signal Recognition Particle (SRP) targets nascent proteins to the Sec61 translocon for import into the endoplasmic reticulum (ER). However, its range of substrates, point of engagement during targeting, and hence full biological impact remain unclear. Here, we examined SRP interactions with the nascent proteome of *S. cerevisiae* during translation and membrane targeting. SRP binds effectively to transmembrane domains (TMDs) as they emerge from the ribosomal tunnel, but only to a minority of cleavable signal peptides. We identify nascent chain features that promote SRP binding, allowing to develop a predictive algorithm. We show SRP performs a role in triaging nascent ER proteins into distinct targeting routes and downstream maturation processes. Furthermore, ribosomes frequently dissociate from the membrane before completing translocation, allowing the chaperone Ssb to assist folding of emerging cytosolic domains. Ribosomes translating multipass membrane proteins are retargeted to the translocon through repeated SRP interactions with internal TMDs, emphasizing collaboration between SRP and chaperones in membrane protein biogenesis.

## Abstract (web summary)

Proteins reach the endoplasmic reticulum (ER) via multiple pathways, often aided by the Signal Recognition Particle (SRP). Here, authors show that SRP acts repeatedly during cotranslational targeting and they map diverse ER targeting routes proteome-wide.

## Introduction

Around 30% of proteins synthesized in the cytosol of eukaryotic cells must be targeted to the endoplasmic reticulum (ER) to enter the secretory pathway. Major hydrophobic targeting signals include N-terminal cleavable signal peptides (SPs) for proteins fully translocated into the ER lumen and transmembrane domains (TMDs) for membrane proteins. The Signal Recognition Particle (SRP) is central to this process^1,2^, recruited to both the targeting signal in the nascent chain and to the ribosome, causing translation to stall^3–5^. SRP binds to the SRP receptor on the ER membrane, promoting docking of the ribosome-nascent chain complex (RNC) to the Sec61 translocon, the protein conducting channel of the ER membrane. SRP thereby dissociates and translation resumes, enabling cotranslational transport of ER luminal protein segments through the translocation pore, while TMDs and SPs enter the membrane bilayer via a lateral gate of the translocon. Although this classical model is well established, key aspects remain incompletely understood or controversial.

First, while SPs were the first type of ER-targeting signal identified to recruit SRP^6^, recent studies suggest that many SP-containing proteins, both in bacteria and yeast, may employ alternative targeting pathways, potentially due to lower hydrophobicity of SPs compared to TMDs^7–11^. Surprisingly, the yeast SRP was suggested to indiscriminately bind the seemingly SRP-independent SPs^12^. Furthermore, the *Saccharomyces cerevisiae* SRP can be genetically deleted^8^, raising questions about redundancy in ER targeting pathways^13^.

Second, while SRP was initially thought to bind RNCs shortly after the full emergence of a targeting signal at the tunnel exit^7,14–16^, alternative models suggest SRP binds ribosomes well before the targeting signal is exposed. Accordingly, SRP binding is either induced by signal´s presence inside the ribosomal tunnel^17,18^, or even occurs before the synthesis of a targeting signal, mediated by the 3′ UTR of the mRNA^12^. The latter model suggests SRP binding to the ribosome neither stalls translation nor causes immediate docking to the translocon.

Third, the role of SRP in ER targeting of multipass membrane proteins is unclear. Translating ribosomes are generally thought to remain docked to the translocon until completion of translation^19,20^ or even longer^21^. However, recent findings indicate that translocation of short nascent chain segments into the ER can be mediated by Oxa1 family members instead of the Sec61 translocon, and proteins containing translocated segments of different lengths may have to shuttle between these different machineries^22,23^. This suggests that ribosome interactions with membrane components require a degree of flexibility that is not accommodated by simpler models and prompts the question whether SRP’s role is complete after the initial targeting of RNCs to the membrane.

In this work, we performed a proteome-wide profiling analysis of SRP engagement with nascent secretory proteins in *S. cerevisiae* to address these issues. This study provides detailed insights on (i) the identity and length of the nascent chains engaged by SRP, (ii) the timing of targeting of RNCs to the membrane, (iii) potential SRP-mediated ribosome pausing, and (iv) the sequence properties of targeting signals affecting SRP binding, guiding downstream nascent chain maturation processes within the ER. We furthermore combined compartment-specific ribosome selection with limited proteolysis and ribosome profiling, demonstrating that ribosomes often dissociate prematurely from the translocon during translocation of multipass membrane proteins, with additional SRP engagements aiding their retargeting. This mechanism allows proper insertion of emerging TMDs into the membrane before completion of nascent chain synthesis and enables cytosolic chaperones to assist in cotranslational folding. Together, our results expand our understanding of the mechanism and scope of SRP-mediated ER targeting, revealing a multifaceted role for SRP in the biogenesis of secretory and membrane proteins.

## Results

### Ribosome exposure of targeting signals is critical for SRP binding

**Fig. 1:**
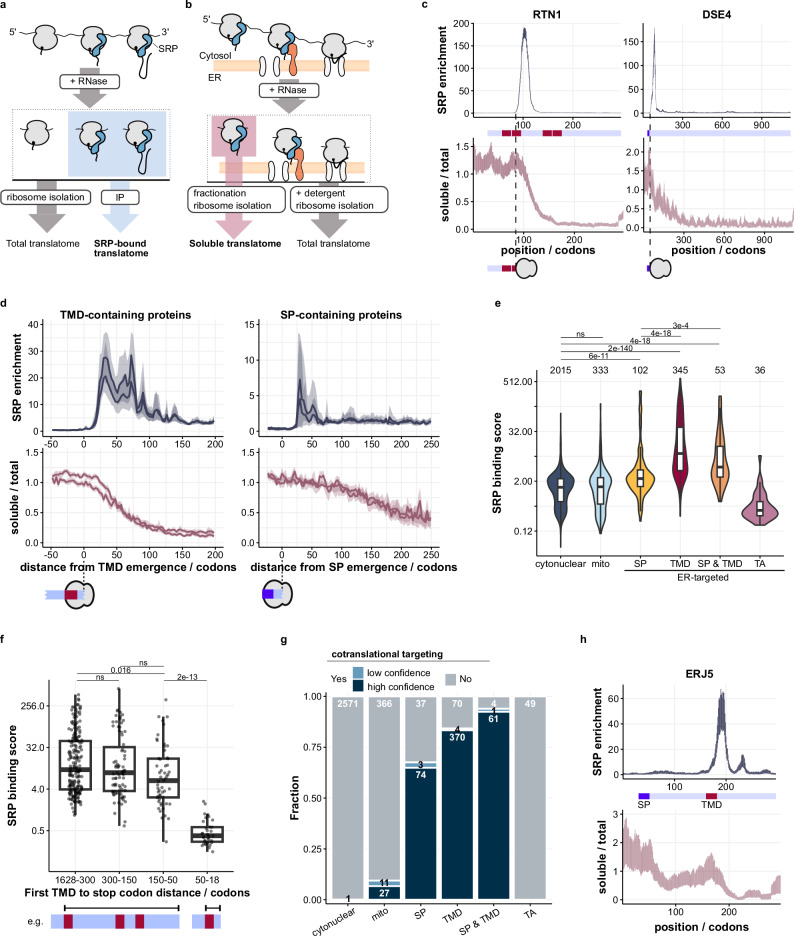
Translatome-wide analysis of cotranslational SRP interactions and membrane targeting. **a** SeRP and **b** Soluble-RP workflows. **c** SRP enrichment (from SRP-SeRP) and targeting (from soluble-RP) profiles of TMD-containing Rtn1 and SP-containing Dse4. Depicted positions of TMDs (red) or SPs (blue) are shifted by 30 codons to indicate when they emerge from the exit tunnel. **d** Metagene profiles showing averaged SRP binding and membrane targeting profiles of all TMD or SP-containing proteins, aligned to the calculated start of emergence of the targeting signal from the exit tunnel. For multipass membrane proteins, the most N-terminal TMD was used for alignment. Each line indicates one biological replicate (*n* = 2). Shaded area indicates the 95% confidence interval derived by bootstrapping. **e** Distribution of SRP binding scores for genes in indicated classes. Shown are mean SeRP binding scores from two biological replicates. Only genes with sufficient read coverage (RPKM > 15) were included; numbers above indicate included genes. The median is indicated by the center line of the boxplot, the box represents the interquartile range (IQC, 25th–75th percentiles), and whiskers extend to the most extreme values within 1.5× the IQC. Significance was assessed by two-sided Wilcoxon rank-sum test with Holm-adjusted *p*-values (adjusted *p*-value> 0.05: not significant). **f** TMD-containing proteins are grouped according to the distance of their first TMD to the stop codon and their average SRP binding scores (*n* = 2), derived as in (**e**), are compared. Boxplot parameters are defined and statistical testing is performed as described in (**e**). **g** Fraction of proteins in each localization category exhibiting no, low-confidence, or high-confidence cotranslational targeting according to soluble-RP. Classification was based on sigmoid curve detection and percent reduction in normalized soluble reads after targeting onset: no (< 25% or non-sigmoidal), yes-low confidence (25–50%), and yes-high confidence (≥ 50%). **h** SRP enrichment and membrane targeting profiles of type I membrane protein Erj5. Ribosome illustrations have been adapted from Koubek J, Schmitt J, Galmozzi CV, and Kramer G (2021). Mechanisms of Cotranslational Protein Maturation in Bacteria. Front. Mol. Biosci. 8:689755. doi: 10.3389/fmolb.2021.689755 under a CC BY license: https://creativecommons.org/licenses/by/4.0/.

We first performed Selective Ribosome Profiling (SeRP) with yeast SRP to explore its nascent chain interactome. We isolated and sequenced ribosome-protected mRNA footprints from all ribosomes and those co-purified with a GFP-tagged SRP subunit, Srp54 (Fig. 1a), creating the “total translatome” and the “SRP-bound translatome”, respectively. By analyzing codon-resolved footprint enrichment in the SRP-bound translatome over the total translatome, we determined when during translation SRP binds RNCs. Our analysis showed SRP selectively binds RNCs after either a TMD (Rtn1) or an SP (Dse4) has fully emerged from the ribosomal exit tunnel (Fig. 1c, upper panel). Metagene profiles of all TMD- and SP-containing nascent proteins aligned to the beginning of tunnel emergence of the targeting signals (assuming 30 residues within the exit tunnel) showed that SRP binds once 20–25 residues of the signal are exposed (Fig. 1d, upper panel). To assess whether the N-terminal GFP tag biased SRP interactions, we performed SeRP with a C-terminally tagged Srp54. The resulting nascent chain binding patterns were highly similar, arguing against tag-dependent effects (Supplementary Fig. 1a).

Our findings contrast with a previous study suggesting that SRP is “pre-recruited” to RNCs before targeting signals are synthesized^12^. It has been proposed that SRP pre-recruitment might especially be relevant during the initial translation of an mRNA in the cytoplasm, and hence may be difficult to detect experimentally without prior enrichment of the soluble, not yet ER-associated fraction of mRNAs. However, our SRP-SeRP analysis from the polysomes contained in the soluble lysate showed no evidence of nascent chain-independent pre-recruitment of SRP (Supplementary Fig. 1b). Furthermore, we reanalyzed data from a previous study^12^ employing a generally used ratio-based analysis for defining RNC binding in SeRP studies^7,24,25^ that included statistical significance testing. Our analysis also did not support early SRP pre-recruitment (Supplementary Fig. 1c) but revealed SRP binding to emerged TMDs. Thus, both datasets corroborate the classical model that in yeast cells, SRP binds only after exposure of nascent targeting signals.

### TMD-containing proteins are the primary substrates of SRP

Using our SeRP data, we identified nascent SRP substrates by calculating SRP binding scores for each protein, reflecting the maximum SRP enrichment along the codon positions (see “Methods”). Our approach revealed that secretory pathway proteins, with the exception of tail-anchored (TA) proteins, had significantly higher scores than cytosolic and mitochondrial proteins (Fig. 1e), in line with previous findings^26–28^. Notably, TMD-containing membrane proteins exhibited the highest median binding scores. SRP was found to bind membrane proteins regardless of the position of their first TMD within the polypeptide, except for C-terminal TMDs of TA proteins, which are accessible only posttranslationally (Fig. 1g and Supplementary Fig. 2a, b). Conversely, a large number of SP-containing proteins had low enrichment scores comparable to non-ER proteins, indicating they are less frequently targeted by SRP (Fig. 1e). This is showcased by type I membrane proteins with both N-terminal SPs and internal TMDs, where SRP selectively bound TMDs (Fig. 1h), leading to higher median scores than those of proteins with only an SP. Overall, our results indicate that the SRP substrate pool in yeast cells is dominated by TMD-containing proteins, independent of the proximity of the TMD to the N-terminus, with most SP-containing proteins excluded.

The unexpected strong preference of SRP for TMDs led us to conduct additional control experiments to further validate our methodology. First, we tested whether the RNase treatment, which is critical for ribosome profiling, destabilizes SRP-substrate interactions. SRP54 co-sedimentation with ribosomes was unchanged with or without prior RNase treatment, indicating that RNase treatment does not disrupt SRP-ribosome association (Supplementary Fig. 1d). Second, we examined whether detergents in our buffers, needed to solubilize membrane-associated ribosomes, interfere with SRP binding, particularly to SP-containing proteins. We performed SeRP experiments without detergent, combined with conditions ± chemical crosslinking using BS^3^ (Supplementary Fig. 1e), previously shown to crosslink SRP to nascent chains^17^. In all conditions tested, we observed the same SRP substrate spectrum, demonstrating that the apparent lack of SRP association with many SP-containing proteins is not due to detergent interference (Supplementary Fig. 1g, h, j). Notably, omitting detergent further pronounced the SRP preference for TMDs, as reflected by increased binding scores of this substrate class, suggesting detergents may partially weaken SRP-substrate interactions. However, detergent-free conditions reduced RNC recovery and overall data quality; therefore, detergent-containing conditions were used for subsequent analyses. Moreover, because crosslinking was performed prior to RNase treatment, these results further argue against the possibility that RNase treatment causes a loss of SRP interactions. Third, we tested whether tagging Srp54 affects SRP function by performing SRP-SeRP using a different GFP-tagged SRP subunit. Cells expressing N-terminally GFP-tagged Sec65 displayed no growth defect (Supplementary Fig. 1f) and SeRP analysis using this strain revealed an unchanged SRP substrate spectrum (Supplementary Fig. 1h). Taken together, these results demonstrate that the apparent underrepresentation of SP-containing proteins is not due to a detection bias but instead reflects the in vivo substrate spectrum of SRP.

To understand why only certain ER-targeting signals bind SRP, we conducted a sequence-logo analysis of all SRP-bound secretory pathway proteins. We examined the identity of the C-terminal 60 amino acids of the nascent chain at the onset of SRP binding. We found that SRP binding coincided with the exposure of a hydrophobic stretch located 10 amino acids from the tunnel exit (Supplementary Fig. 2c). This stretch was enriched in Leucine (L), Isoleucine (I), Phenylalanine (F), Valine (V) as well as Tryptophan (W) residues in no particular order, while being depleted of charged residues, Serine (S), and helix breaking Proline (P). These findings suggest a stringent hydrophobicity threshold may limit SRP binding to most SPs.

### Efficient cotranslational membrane targeting of both SRP substrates and non-substrates

Building on our finding that most SP-containing proteins in yeast are not SRP substrates (Fig. 1e), we investigated how and when these proteins reach the ER. We employed a modified ribosome profiling protocol, termed soluble-RP, to track when RNCs associate with the ER membrane. This technique quantitatively assesses RNC targeting to the ER membrane by measuring their depletion from the soluble fraction of cell lysates. For each gene, we computed the codon-wise ratio of normalized ribosome footprints in the soluble translatome to those in the total translatome, thereby quantifying the distribution of ribosomes between the cytosol and the membrane fraction throughout translation (see Supplementary Fig. 3 for details). As mRNAs are nuclease digested prior to fractionation, the method distinguishes RNCs that are directly associated with the membrane from those that are only indirectly tethered via a membrane-associated polysome (Fig. 1b). As translation progressed, we observed RNCs of both TMD- and SP-containing proteins disappearing from the soluble fraction, indicating cotranslational targeting to the ER membrane (Fig. 1d). For proteome-wide annotation of cotranslationally targeted proteins we analyzed the soluble-RP data using a sigmoidal fitting algorithm^29^, and compared the data with our SRP binding profiles (Supplementary Data 2–4). We found that among all proteins reliably detected, the vast majority of ER-destined proteins were targeted cotranslationally with very high efficiency: 84% of TMD-containing proteins, 68% of SP-containing proteins, and 94% of proteins containing both SP and TMD (Fig. 1g), with median targeting efficiencies of 85, 81, and 80%, respectively.

Proteins not targeted cotranslationally primarily had C-terminal targeting signals (TMDs in the last C-terminal 150 amino acids) or are SP-containing proteins shorter than 300 residues (Supplementary Fig. 2d). Our analysis covers approximately two-thirds of ER-destined proteins due to read coverage thresholds required for reliable annotation. To extend our findings to all annotated ER proteins and to account for the disproportionate exclusion of shorter proteins by such thresholds, we modeled the probability of cotranslational targeting as a function of protein length using logistic regression. This recapitulated the experimentally observed prevalence of cotranslational targeting for TMD-containing proteins (84%), while predicting a slightly lower yet still predominant fraction for SP-containing proteins (61%). Overall, these results indicate that most SP-containing proteins are cotranslationally targeted to the ER despite not binding SRP, suggesting that they may utilize alternative targeting pathways. In contrast, posttranslational targeting in yeast primarily occurs when targeting signals is located towards the C-terminus, and emerge too late for cotranslational engagement.

Examining the coordination of targeting with ongoing translation, we found that SRP substrates exhibited immediate and nearly complete disappearance of RNCs from the cytoplasm following SRP binding, which thereafter is followed by SRP dissociation (Fig. 1c bottom panel). Metagene analysis revealed that membrane targeting of SP-containing proteins was more delayed compared to TMD-containing proteins, indicating a difference in the synchronization of protein synthesis and ER targeting. Proteins with both SPs and TMDs followed more complex targeting pathways; for example, the membrane association of a subset of Erj5 RNCs began with SP emergence, but targeting of all nascent chains completed only after TMD exposure and SRP binding (Fig. 1h).

We also identified 27 mitochondrial proteins showing cotranslational targeting to the membrane fraction (which contains mitochondrial and ER membranes) (Fig. 1g). These 27 proteins are destined to various mitochondrial compartments, including outer and inner membranes and matrix (Supplementary Fig. 4, see respectively Tom70, Tim50, and Mef1). Membrane-embedded mitochondrial proteins were targeted shortly after their TMDs emerged at the tunnel exit, while matrix proteins were often targeted late during translation, with little correlation to transit peptide emergence. Overall, these findings suggest that these proteins are cotranslationally targeted to mitochondria, supporting previous reports^30,31^. Few of these nascent proteins (e.g., Tim50) were bound by SRP, but their cotranslational membrane targeting was detectable even in cells lacking SRP (see below). These proteins may be clients of the recently described ER-SURF pathway, which initially targets RNCs to the ER with the help of SRP or by using alternative mechanisms, from where proteins are directed to mitochondria posttranslationally^32^.

### Signal peptide-containing proteins are targeted to the ER membrane by various pathways

**Fig. 2:**
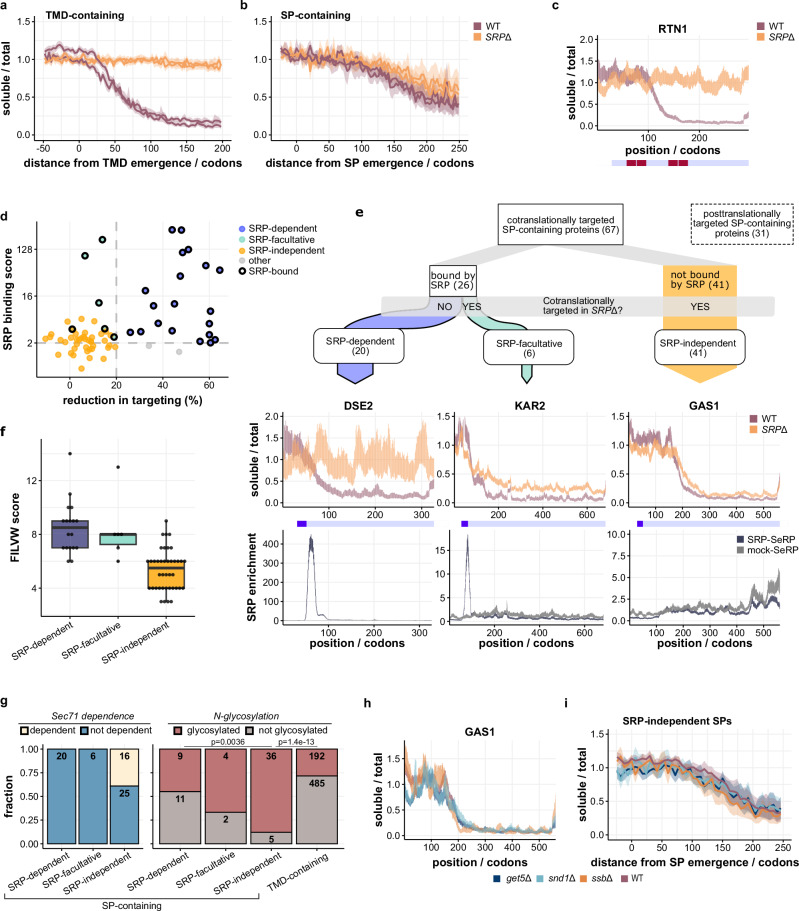
Alternative targeting mechanisms used by SP-containing proteins. **a** Metagene targeting profiles of all TMD- or **b** SP-containing proteins, aligned at the start of targeting signal exposure. Experiments were performed using WT (pink) or *srp54*Δ (*SRP*Δ, yellow) yeast (*n* = 2). Shaded areas indicate the 95% confidence interval. **c** Targeting profile of the TMD-containing protein Rtn1 in WT and *SRP*Δ strains. **d** Comparison of SRP-binding and the dependence on SRP for targeting of SP-containing proteins. SRP binding scores are compared to the percent difference of targeting efficiency (WT versus *SRP*Δ). The manually curated group of SRP-binders is outlined in black. A minimal value of 20% difference in targeting efficiency was used as a threshold for SRP-dependency. **e** Scheme outlining the classification of SP-containing proteins based on their SRP-dependence (top) and example profiles of targeting (middle) or SRP enrichment (bottom, dark blue) from each class. Mock-SeRP profiles (grey), performed using a strain lacking any GFP-tag (Minoa et al., 2024), were included to illustrate background enrichment. **f** Total count of Phe, Ile, Leu, and Trp residues in hydrophobic region of SPs in each SRP-dependence category. Boxplot parameters are defined as in (Fig. 1e). **g** Number of proteins in each SRP-dependence class that were found to be Sec71-dependent for cotranslational ER membrane targeting according to Jan et al., 2014 (left) or are annotated to be glycosylated according to UniProt (right). **h** Targeting profile of SRP-independent Gas1 protein, in WT (pink), *get5*Δ (dark blue)*, snd1*Δ (light blue), and *ssb*Δ (orange) strains (*n* = 1). **i** Metagene targeting profiles of SRP-independent SP-containing proteins, in WT, *get5*Δ*, snd1*Δ, and *ssb*Δ strains (*n* = 1). Shaded areas indicate the 95% confidence interval derived by bootstrapping.

The existence of multiple, potentially redundant ER-targeting pathways raises the possibility that SRP binding is not always equivalent to SRP dependence for cotranslational targeting. To identify proteins that strictly require SRP for membrane targeting, we conducted soluble-RP analysis in *srp54*Δ cells (*SRP*Δ). Although not a complete deletion of SRP, loss of the functionally essential SRP54 subunit abolishes SRP-mediated targeting, even if the remaining subunits assemble on the SRP RNA to form a subparticle^33^. In the absence of SRP, 93% of membrane proteins that are cotranslationally targeted in wild-type cells lose this mode of targeting (Fig. 2a, c and Supplementary Fig. 5a), suggesting that those proteins that still reach the ER membrane are likely inserted posttranslationally. An exception was the widely used model SRP substrate Dap2 (DPAP-B), also identified as an SRP substrate by our SeRP approach (Supplementary Fig. 5b). Previously, Dap2 was found to be targeted to the ER membrane in cells lacking SRP via an unknown targeting mechanism following an adaptive response to SRP depletion^34^. We observed that Dap2 is cotranslationally targeted also in the absence of SRP, albeit more slowly (Supplementary Fig. 5b). This makes Dap2 a unique case where cotranslational targeting of a TMD-containing protein persists without SRP.

In contrast, the cotranslational targeting of most SP-containing proteins, including some that engaged SRP, remained unaffected by SRP absence (Fig. 2b and Supplementary Fig. 5a). In total, we classified 67 SP-containing proteins into three categories, SRP-dependent, SRP-facultative, or SRP-independent, based on their SRP engagement efficiency (binding score) and the effect of SRP deletion on their cotranslational targeting (Fig. 2d, e). Among these, 20 were SRP-dependent, relying on SRP for efficient targeting (Fig. 2e and Supplementary Fig. 5c). Six were SRP-facultative, as they bound SRP but were able to use alternative pathways (Fig. 2e and Supplementary Fig. 5d), enhancing targeting robustness. These rare proteins included the ER luminal Hsp70 Kar2 and its nucleotide exchange factor Lhs1. Lastly, 41 proteins were SRP-independent, as neither efficiency nor timing of targeting was affected by SRP. This group included previously identified SRP-independent proteins: Prc1 (CPY), Gas1, and Pdi1^8,9^ (Fig. 2e and Supplementary Fig. 5e). A comprehensive annotation of SRP-dependence, quantification of targeting efficiencies, and targeting onsets in each strain is provided in Supplementary Data 3.

Collectively, our findings indicate that most SP-containing proteins in yeast cells are not SRP substrates, while nearly all TMD-containing proteins depend on SRP for cotranslational targeting, with redundancy in this transport mode limited to a few proteins with key roles in ER proteostasis.

### Properties of SRP-bound signal peptides

We set out to identify key properties of nascent chains that regulate SRP binding to SP-containing proteins. SPs consist of a positively charged N-terminal region, a hydrophobic central region (h-region), and a C-terminal region. Generally, h-regions of SPs are shorter and less hydrophobic than TMDs^10,35^. Consistent with this, SRP-dependent and SRP-facultative SPs have longer and more hydrophobic h-regions, resembling TMDs (Supplementary Fig. 6a, b). A previously used compound score based on length and hydrophobicity of SPs predicted SRP dependence^10^, but it wrongly annotated many SRP-dependent SPs as SRP-independent (Supplementary Fig. 6c, d). We now refined this prediction by combining two criteria: First, SRP binding is triggered when the total number of Phe, Ile, Leu, Val, and Trp (FILVW score) in the SP´s hydrophobic region exceeds 6 (Fig. 2f and Supplementary Fig. 6e). This criterion is fulfilled in 97% of the first TMDs of membrane proteins, in line with SRP’s strong preference for binding them. Second, the presence of Ser and helix-breaking Gly or Pro residues penalizes SRP binding (PGS score, Supplementary Fig. 6f, g). Combining both criteria enhanced the prediction accuracy (Supplementary Fig. 6e), yet the distinction between SRP-dependent from SRP-facultative proteins remains unclear.

Previous work demonstrated that in yeast, SRP-independent proteins can be co- or posttranslationally translocated via Sec61 translocons associated with the SEC complex (Sec62, Sec63, Sec71, and Sec72)^36–38^, with the help of ER luminal Hsp70 Kar2 to ratchet the nascent chains into the ER^39^. Our findings align with earlier work on secretory proteins that rely on Sec71 for ER targeting^38^, revealing overlap with our SRP-independent classification (Fig. 2g).

In mammals, Sec62 and Sec63 are conserved and form a complex with Sec61 and luminal Hsp70^40^, whereas homologs of Sec71 and Sec72 are absent. Similar to yeast, human Sec62 and Sec63 can function downstream of both co- and posttranslational targeting pathways, and are particularly important for efficient gating of weak SPs^41–44^. These observations suggest conservation of the core mechanistic principles of the complex across species, especially regarding substrates with less hydrophobic targeting signals. However, it remains unclear whether mammalian Sec62–Sec63 acts downstream of an SRP-independent targeting pathway or assists signal peptide gating following SRP-mediated targeting. Analysis of FILVW-PGS scores, which predict SRP binding in yeast, reveals a clear separation between SPs and TMDs in human cells as well (Supplementary Fig. 6h). This suggests that cytosolic recognition of these targeting signals in human cells could also involve alternative pathways.

Curiously, also in mammalian cells, the binding of the SRP receptor displaces Sec62 from the translocon, suggesting these two components distinguish functionally different translocons, responsible for SRP-dependent versus SRP-independent translocation^45^. We hypothesized that SRP´s substrate preference directs ER-targeted proteins toward specific translocation pathways involving different translocons. These translocons may associate with specific auxiliary factors, linking targeting pathway usage to distinct downstream maturation processes, including N-glycosylation. Testing this hypothesis in yeast, we compared the frequency of N-glycosylation across different protein classes, and indeed found that glycosylation was most prevalent among SRP-independent SP-containing proteins, followed by SRP-facultative ones (Fig. 2g), while SRP-dependent SP- and TMD-containing proteins were much less frequently N-glycosylated.

In summary, our results uncover clear principles for SRP binding in *S. cerevisia*e. The observed correlations suggest a model in which the use of alternative targeting systems represents the first decision-making step guiding subsequent maturation processes within the ER.

### Targeting of SRP-independent and SRP-facultative SP-containing proteins

Intriguingly, soluble-RP revealed that targeting of SRP-independent secretory proteins often occurs with a significant delay of 100 amino acids or more after SP synthesis (Fig. 2e and Supplementary Fig. 5e), contrasting with the immediate SRP-mediated targeting right after signal exposure. For SRP-facultative Dse4, this delay was only evident in *SRP*Δ cells (Supplementary Fig. 6i), likely due to a switch from an SRP-dependent to an alternative targeting pathway. As suggested by previous studies, we evaluated the SND pathway, the GET pathway, and targeting via the Hsp70 homolog Ssb^10,37,46^ as potential mechanisms for targeting SRP-independent and SRP-facultative SP- containing proteins. However, soluble-RP in *snd1*Δ, *get5*Δ, and *ssb*Δ strains showed no major effects on cotranslational targeting of SRP-independent proteins, or other ER-targeted protein classes in general (Fig. 2h, i, I, Supplementary Fig. 6j, k), indicating that these pathways do not solely account for the targeting of these proteins. Still, it is possible that multiple systems operate redundantly, masking detection if only one pathway is inactivated.

### SRP binding causes translation slowdown and ribosome collisions

The translation arrest activity of eukaryotic SRP, proposed to enhance membrane targeting, was first observed in a heterologous system using wheat germ extracts and canine SRP^3^. Studies in homologous yeast^4^ and mammalian systems^47^ report a more moderate reduction in elongation rates, or no detectable effect^48^, suggesting the magnitude of arrest may depend on the experimental context. Previously published ribosome profiling data^12^ and 30 nt footprint data from our present study did not show an accumulation of ribosomes during SRP binding that would be expected if elongation were substantially slowed at this stage (Supplementary Fig. 7a). This discrepancy may be due to SRP binding causing translation pausing and ribosome collisions, which hinder nuclease digestion of mRNA segments between ribosomes. This generates longer footprints^49^ that are not captured in standard 30 nt footprint profiling (Supplementary Fig. 7b). We therefore analyzed 60 nt long footprints protected from collided ribosomes.

**Fig. 3:**
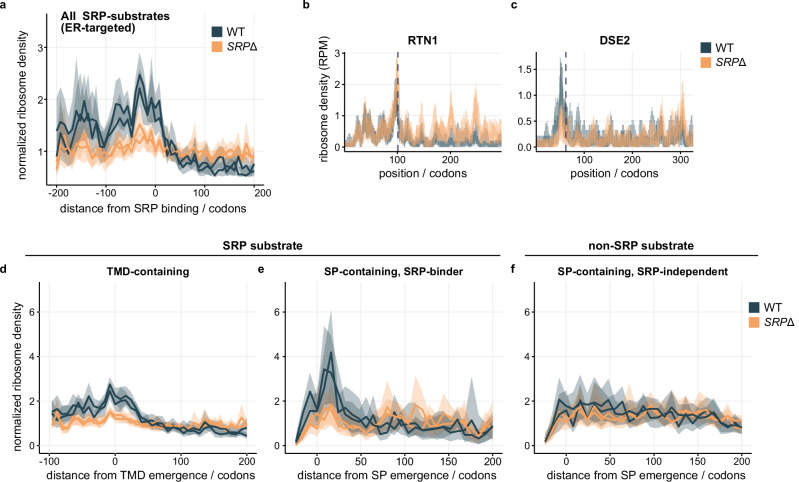
Translation slow-down during SRP-mediated targeting causes ribosome collisions. **a** Metagene profile of collided disomes on ER-targeted proteins in WT (dark blue) and *SRP*Δ (orange) yeast strains, showing the average distribution of the leading ribosome in collided disomes, aligned at the onset of SRP binding. The start of the first SRP binding period detected for each gene was used for alignment (*n* = 2). Shaded area indicates the 95% confidence interval. **b**, **c** Single gene profiles showing the distribution of collided disomes on RTN1 and DSE2 mRNAs. Dashed lines indicate the position of maximal SRP enrichment (see Fig. 1c). **d** Metagene profile showing the average distribution of the leading ribosome in collided disomes for TMD-containing proteins, **e** for SRP-bound SP-containing proteins, **f** for SRP-independent SP-containing proteins, aligned at the position when the first targeting signal starts to emerge from the exit tunnel. Shaded areas indicate the 95% confidence interval.

This approach revealed an accumulation of collided disomes at established stalling sites, such as polyproline stretches (Supplementary Fig. 7c), and, more modestly, during translation stages when SRP binds ER-destined proteins (Fig. 3a). Suggesting that SRP binding mediates a translation slowdown, collisions on SRP-substrates were generally reduced in *SRP*Δ cells, while polyproline-mediated collisions on cytonuclear transcripts were unaffected. Notably, SRP is not the sole factor influencing translation, as collisions were detectable, though on average less frequent, in the *SRP*Δ strain (Fig. 3b–e). Overall, among ER-destined proteins, translation of SRP substrates showed more pronounced ribosome stalling than non-substrates in WT and *SRP*Δ mutants (Fig. 3d–f). These findings support the model that intrinsic mRNA or nascent chain features slow translation, likely facilitating SRP binding^50^. Our data suggest that this slowdown, intensified by SRP binding, persists until ER membrane targeting is achieved.

### SRP skipping of N-terminal TMDs

**Fig. 4:**
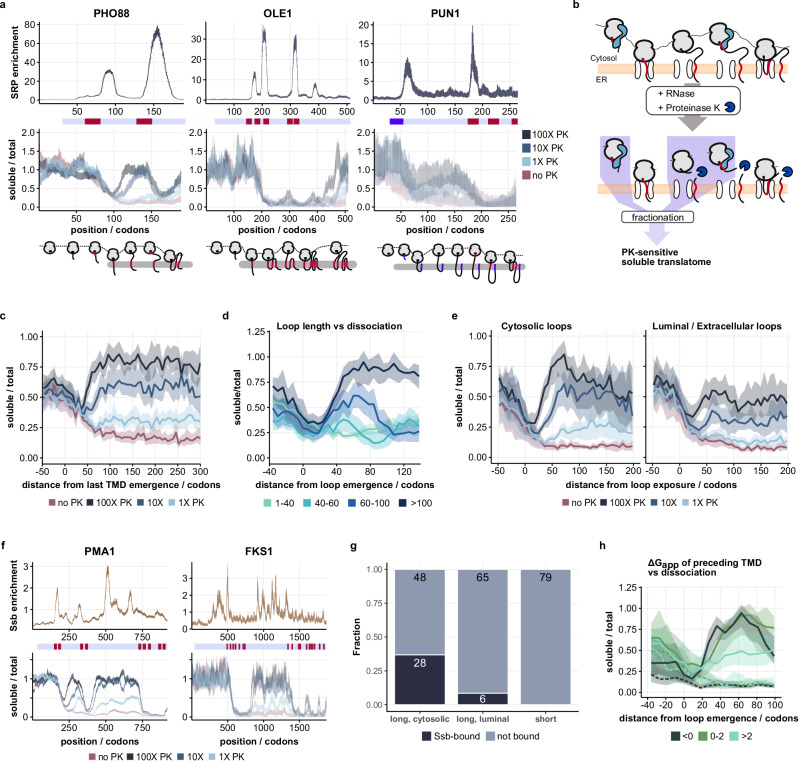
SRP engages multiple TMDs of a nascent chain and facilitates the retargeting of ribosomes that have dissociated from the ER membrane. **a** Single gene profiles showing SRP enrichment (top) and soluble-RP with PK treatment (bottom). **b** Experimental setup of soluble-RP with PK treatment. **c** Metagene profiles of soluble-RP with PK treatment of all TMD-containing proteins, aligned at the position where their most C-terminal TMD emerges from the exit tunnel (*n* = 1). Shaded area indicates the 95% confidence interval. **d** Metagene profile of soluble-RP with PK-treatment (100× PK) of TMD-containing proteins, aligned at the position where internal loops are starting to emerge from the exit tunnel, shown for indicated ranges of loop lengths. **e** Metagene profiles of soluble-RP with PK treatment. Compared are loops longer than 50 amino acids with a cytosolic (left) or luminal/extracellular (right) topology. Shaded area indicates the 95% confidence interval. **f** Single gene profiles of Pma1 and Fks1, top: Ssb enrichment measured by Ssb1 SeRP (Döring et al., 2017), bottom: soluble-RP with PK treatment. **g** Ssb binding preferences after cotranslational ER-targeting. Fraction of membrane proteins, categorized by the presence of long (> 100 amino acids) cytosolic loops, long luminal loops, or only short (< 30 amino acids) loops, that engage Ssb following cotranslational targeting to the ER are indicated. Ssb binding was assessed by identification of Ssb-binding periods measured by Ssb1 SeRP (Döring et al., 2017). **h** Metagene profile of soluble-RP with PK-treatment, for genes binned according to the indicated Δ*G*_app_ values of the preceding TMD (noPK: dashed lines, 100× PK: solid lines), aligned at the position of internal loop emergence. Shaded area indicates the 95% confidence interval. Ribosome illustrations have been adapted from Koubek J, Schmitt J, Galmozzi CV, and Kramer G (2021) Mechanisms of Cotranslational Protein Maturation in Bacteria. Front. Mol. Biosci. 8:689755. doi: 10.3389/fmolb.2021.689755 under a CC BY license: https://creativecommons.org/licenses/by/4.0/.

Remarkably, our SeRP data revealed that in yeast cells SRP engages not only the first exposed TMD of multipass membrane proteins but also downstream TMDs (Fig. 1d upper panel left, Fig. 4a, and Supplementary Fig. 8a, b). Interactions of SRP with multiple TMDs, also observed in *Escherichia coli*^7^, suggest a conserved but not fully understood mode of SRP action. To explore the underlying mechanisms, we examined alternative models. One model is that SRP may occasionally miss the initial TMD, targeting the nascent chains to the ER membrane only upon binding to downstream TMDs, resulting in a split SRP binding profile (S8C, top panel). Our comparison of SRP binding periods with membrane targeting data from soluble-RP supports this. For example, Pga3 nascent chains show incomplete targeting when SRP binds to the initial emerging TMD, which is completed only after SRP binding to the second TMD (Supplementary Fig. 8a). However, we also observed cases where SRP fully skipped the N-terminal TMD (Supplementary Fig. 8a, DGK1), leading to delayed membrane targeting. We reasoned that TMD skipping likely results from SRP’s limited affinity. To investigate this, we compared SRP binding with the predicted free energy differences of membrane insertion (Δ*G*_app_) for emerging TMDs^51^. TMDs with more positive Δ*G*_app_ values are shorter, less hydrophobic, and harder to insert. Testing whether this feature may also affect SRP recognition indeed showed that TMDs with higher Δ*G*_app_ values were more frequently skipped by SRP (Supplementary Fig. 8d).

A second model posits that SRP binding is influenced by the delayed emergence of N-terminal TMDs with an *N*_cyto_ topology from the ribosomal exit tunnel (Supplementary Fig. 8c, bottom panel). For *N*_cyto_ topologies, nascent chains must loop during insertion into the translocon, with the N-terminus facing the cytosol^52^. If looping occurred already within the ribosomal exit tunnel, nascent chain emergence would be delayed, resulting in delayed SRP binding and targeting. Ribosome internal looping has been shown by in vitro biochemical assays for the first *N*_cyto_ TMD of the *E. coli* inner membrane protein EmrD, while the *N*_exo_ TMD of the *E. coli* inner membrane protein LepB does not loop inside the ribosome^53^. Analyzing previously published *E. coli* SRP SeRP data^7^, we found that SRP’s interaction profile was consistent with looping of the nascent chain in the exit tunnel: While SRP binds timely to the 1st TMD of LepB (Supplementary Fig. 8f), the SRP binding to nascent EmrD is delayed until the second TMD emerges, suggesting that nascent chain looping within the ribosome exit tunnel may hinder early SRP recognition. Testing our SeRP dataset in yeast also showed delayed SRP binding for some *N*_cyto_ proteins (e.g., GUP1), even when the 1st TMD is highly hydrophobic (Supplementary Fig. 8b). We considered the postulated loop formation inside the tunnel may be stochastic, creating two different nascent chain populations that recruit SRP at different time points during translation, as observed for HXT2. In agreement with this possibility, metagene profiles of *N*_cyto_ or *N*_exo_ membrane proteins revealed that *N*_cyto_ membrane proteins more frequently exhibit additional SRP binding periods 25–50 codons after the 1st binding event (Supplementary Fig. 8e). Notably, *N*_cyto_ membrane proteins more frequently contain their first two N-terminal TMDs in close proximity compared to *N*_exo_ membrane proteins. This feature may help explain the differences observed under the skipping model (S8c, top panel). Alternatively, a proximally positioned second TMD may be critical for loop formation within the ribosomal exit tunnel through interactions between the two transmembrane segments. Overall, these findings suggest that the timing of SRP recruitment and membrane targeting is influenced by various features of the nascent chain, including the hydrophobicity of the N-terminal TMDs and possibly their propensity to form loops inside the exit tunnel.

### SRP repeatedly binds translocon-dissociated ribosomes for retargeting during insertion of multipass membrane proteins

Our data show that SRP binding can also occur after ER membrane targeting is completed and ribosomes are fully membrane-associated (Fig. 4a “no PK”), correlated with the emergence of downstream TMDs. We hypothesized that ribosomes may dissociate from the translocon before translation ends, remaining connected to the membrane via the nascent chain. For partially inserted multipass membrane proteins, this premature dissociation may require SRP retargeting to reestablish translocon docking when a downstream TMD emerges.

To differentiate between translocon-docked ribosomes and those only connected to the membrane via the nascent chain, we introduced limited proteolysis in our soluble-RP protocol. Limited amounts of protease will preferentially degrade unfolded nascent chains, releasing ribosomes from the membrane fraction that are not docked to the translocon. We treated RNase-digested cell lysates with increasing concentrations of Proteinase K (PK) prior to depletion of membrane-bound ribosomes by centrifugation and ribosome profiling of the soluble fraction (Fig. 4b). We found that after initial membrane targeting, ribosomes synthesizing multipass membrane proteins alternate between PK-resistant and PK-sensitive states. TMD emergence coincided with short periods of PK-insensitive membrane association, followed by PK-sensitive membrane association during synthesis of loops (Fig. 4a). Exposure of a subsequent TMD triggered SRP rebinding and membrane retargeting, detectable as another PK-insensitive period (Fig. 4e and Supplementary Fig. 8g). Over 75% of ribosomes dissociated from the membrane after the last TMD insertion but before translation termination (Fig. 4c), suggesting that most membrane proteins are synthesized by ribosomes not stably docked on the translocon.

To exclude the possibility that ribosome undocking may occur after cell lysis, we analyzed ribosome profiling data from the Weissman lab, which measured the in vivo proximity of ribosomes to the Ssh1-translocon, a homolog of the Sec61-translocon^38^. After initial ER-targeting, translation phases with reduced ribosome proximity to Ssh1 exist and coincide with periods of increased PK sensitivity (Supplementary Fig. 8h, j, k). Taken together, these results indicate that RNC-translocon interactions are dynamically lost during translation and reestablished with emergence of downstream TMDs. Therefore, many multipass membrane proteins require repeated SRP action for efficient insertion of all TMDs.

### Determinants of ribosome undocking

We investigated the nascent chain characteristics associated with ribosome undocking. Generally, translocon dock-off occurred 40–50 residues after synthesis of a loop, corresponding to the emergence of a 10–20 residue-long stretch from the exit tunnel (Fig. 4a, d). While shorter loops may still permit undocking, the minimal distance between ribosome and membrane likely hampers efficient PK digestion. Increasing loop lengths enhanced dissociation, before downstream TMDs emerge and initiate redocking (Fig. 4d). Notably, nearly all ribosomes synthesizing loops over 100 amino acids lengths were solubilized by PK (Fig. 4d), indicating the generality of this phenomenon.

Next, we examined the relationship between protein topology and ribosome dissociation. Undocking occurred more frequently during the synthesis of cytosolic loops compared to ER-luminal internal loops and SP-containing proteins (Fig. 4e and Supplementary Fig. 8l). We hypothesized that ribosome dock-off may preferentially facilitate cytosolic loop biogenesis for three primary reasons: (i) it eases their release into the cytoplasm compared to passing through the narrow opening between translocon and ribosome^54^, (ii) it provides spatial freedom for nascent chain compaction and native folding, and (iii) it creates room for cytosolic chaperones to assist in folding. To test the correlation between undocking during loop synthesis and chaperone engagement, we compared our data with cotranslational interaction profiles of Ssb^55^, an Hsp70 homolog in yeast that binds the ribosomal tunnel exit at the universal adaptor site also used by the translocon^56^. Supporting our hypothesis, Ssb specifically engages translocon-dissociated RNCs during loop synthesis (Fig. 4f). Overall, Ssb binds 37% of nascent ER membrane proteins harboring long cytosolic loops after their initial membrane targeting, compared with 8% of proteins containing long luminal loops and none of the membrane proteins composed exclusively of short loops (Fig. 4g). We propose that transient translocon dissociation is a fundamental aspect of multipass membrane protein biogenesis, facilitating chaperone-assisted cotranslational folding of cytosolic domains.

We noted instances where ribosome undocking was prevented during the synthesis of cytosolic loops longer than 50 amino acids (e.g., Ole1, Erg4 in Supplementary Fig. 8g, m). Analysis of these nascent chains revealed that such loops were generally preceded by TMDs with positive Δ*G*_app_ values, indicating lower hydrophobicity and reduced insertion efficiency into the ER membrane^51^. Additionally, at the metagene level, ribosome undocking was more common for loops following TMDs that are inserted more efficiently (Fig. 4h). The lateral diffusion of TMDs into the membrane was reported to exert a pulling force on the nascent chain that is proportional to TMD hydrophobicity and efficiency of membrane insertion^57^. We speculate that this pulling force, combined with the lateral movement of TMDs into the membrane, may destabilize ribosome interactions with the translocon, leading to ribosome undocking. Accordingly, we found that TMDs preceding long cytosolic loops are biased towards more negative Δ*G*_app_ values (Supplementary Fig. 8n, o). These patterns suggest an evolutionary selection for elevated hydrophobicity of TMDs preceding long cytosolic loops, which may promote efficient undocking and facilitate the engagement of ribosome-associated chaperones and the folding of cytosolic domains.

## Discussion

In this study, we employed three ribosome profiling methods—SRP-selective RP, soluble-RP, and proteinase solubilized-RP—to capture the cotranslational ER targeting of the yeast proteome. Our codon-resolved analysis revealed the nascent chain interactome of SRP and the translation states at which it binds its substrates. In support of the signal hypothesis^58^, we found that SRP recognizes nascent chain signals as they emerge at the tunnel exit. More unexpectedly, we also show that SRP binds TMDs with high specificity, while most SPs neither bind nor require SRP for targeting. This is distinct from the proposed ability of SP-containing proteins to utilize additional cotranslational targeting pathways, as these proteins are not engaged by SRP even under wild-type conditions. Redundancy in cotranslational targeting appears to be limited and to function primarily as a backup mechanism, as only a small subset of SRP-bound proteins continues to be targeted cotranslationally in the absence of SRP. This subset includes proteins such as Kar2 and Dap2, which were previously reported to regain ER-targeting capability following an adaptation process to SRP depletion^34^.

The strong substrate selection by SRP observed in our eukaryotic model appears evolutionary conserved, as *E. coli* SRP has a similar preference for TMDs^7^, and remains to be investigated in higher eukaryotes. Bioinformatic analysis of the binding data reveals that TMD hydrophobicity is crucial for SRP binding, allowing it to bind to most nascent ER membrane proteins. In contrast, the shorter and less hydrophobic SPs and TMDs of mitochondrial proteins^32^ preclude SRP binding. In agreement, the substrate binding site in Srp54 accommodates ~16 hydrophobic residues, whereas the hydrophobic regions of SPs must be shorter than 17 residues for efficient signal peptidase cleavage^35,59^, and in fact has a median length of 11 residues^60^. This length limitation may have driven the evolution of alternative ER targeting pathways. Our findings further suggest that SRP binding to only a subset of ER proteins has a functional significance.

The existence of distinct translocation hubs on the ER membrane has been previously proposed, each enriched in specific factors facilitating specialized modifications such as glycosylation or lipid anchoring and hence tailored for proteins with unique physicochemical properties^61^. Recent studies have identified additional types of translocation machineries in mammalian cells that may assemble dynamically in response to features of the nascent chain within the translocon, forming specialized channels for secretory versus multipass membrane proteins^62–64^. The correlations we observe in our yeast model suggest an alternative model that triaging of protein classes to different translocation hubs may already start at the ribosomal tunnel exit, determined by SRP binding to a subset of ER-destined proteins based on the information encoded in their targeting signal. According to our model, the proteins bound by SRP, primarily membrane proteins, are directed to SRP receptor-containing translocons, which may associate with the multipass translocon (MPT) and/or the ER membrane complex (EMC), which mediate processes specific for membrane proteins such as insertion of short luminal segments or chaperoning of TMDs^65,66^. In contrast, SRP receptor-free translocons can be associated with the glycan-conjugating complex OST, the SEC-complex with BIP, and the TRAP complex, to support membrane transport, glycosylation, and luminal folding of SP-containing luminal and secretory proteins^67,68^.

The SRP-independent cytosolic targeting routes used by SP-containing remain unclear, with previous studies showing conflicting evidence for both posttranslational^9^ and cotranslational^11^ targeting. Our findings indicate that many SP-containing proteins in yeast cells are cotranslationally targeted via SRP-independent pathways. Although we could not identify the specific alternative pathway used, pathways like SND, GET, and Ssb-mediated targeting may contribute to a redundant group of SRP-independent pathways. Of note, the significant delay between signal exposure and membrane engagement for these proteins distinguishes them from SRP substrates. Interestingly, some SP-containing proteins, such as Prc1 and Pep4, can be targeted to the ER with reduced efficiency even without their SPs^69,70^, suggesting a potential secondary C-terminal targeting signal.

Our study also addresses a controversial aspect of SRP-mediated targeting: the signal that first recruits SRP to its substrate RNCs. We found no evidence of substrate-specific SRP interactions before the targeting signal emerges from the tunnel exit. While we cannot formally exclude that weaker interactions escape detection in SeRP experiments, the apparent nascent chain-independent pre-recruitment of SRP^12^ may reflect inherent methodological challenges that can confound interpretation if not addressed in the data analysis. In particular, inhibition of translation elongation in vivo does not prevent ongoing membrane targeting. Subsequent removal of membrane fractions may therefore deplete SRP-RNC complexes from the soluble fraction. Because SeRP affinity purifications include nonspecific RNC binding to the resin, IP samples can instead be dominated by the elevated read density at the 5′ end of open reading frames from the soluble input sample, which decreases upon membrane targeting. Bona fide SRP binding events can be masked by this asymmetric RNC distribution, while the elevated 5′ read density resembles nascent chain-independent SRP recruitment if not corrected by normalization to the input.

Investigating the possible correlation of SRP engagement and translational slowdown, we directly compared proteome-wide data on ribosome collisions to our SRP binding profiles and indeed found enhanced ribosome collisions upon SRP binding. Interestingly, these collisions persisted, albeit at reduced levels, even in SRP’s absence, specifically for ER-targeted proteins that are SRP substrates. This suggests that the translation of these mRNA segments is inherently slow, facilitating SRP binding^50^, which further amplifies the slowdown. Of note, the binding of SRP to nascent proteins containing C-terminal TMD targeting sequences does not ensure complete membrane targeting prior to translation termination (Supplementary Fig. 1f), which may suggest that SRP binding does not entirely stall but rather slows down translation. Alternatively, only a subset of these very late signals may be recognized and targeted by SRP.

Based on our findings, we propose that SRP binding and subsequent ER membrane targeting require the exposure of a nascent chain-encoded targeting signal. Our model assigns SRP a role that extends beyond initial membrane targeting. Through iterative cycles of SRP binding, ER targeting, TMD insertion, ribosome undocking, and SRP rebinding, SRP effectively stitches nascent multipass membrane proteins onto the ER membrane. Our data do not exclude SRP-independent retargeting of specific undocked RNCs, as not all undocking events are followed by SRP rebinding. However, prior in vitro translocation experiments provide preliminary evidence that, for certain substrates, efficient retargeting may depend on SRP rebinding^71^. In this context, insertion of an internal TMD was reported to become SRP-dependent when the preceding loop is extended, consistent with our observation that long loops promote dissociation of ribosomes from the ER membrane. We further find that synthesis of long cytosolic loops, coinciding with a freed ribosomal tunnel exit, permits recruitment of the chaperone Ssb for folding assistance. Future studies may identify additional ribosome-associated chaperones that engage nascent chains during this time window, such as the ribosome-associated complex RAC, to facilitate maturation of cotranslationally targeted multispanning membrane proteins. The frequent SRP reassociation events detected by our high-throughput approach, together with prior in vitro evidence showing defective internal TMD insertion when these interactions are disrupted, support a model in which SRP functions beyond initial ER targeting: In this framework, SRP surveils and facilitates downstream integration events, thereby promoting correct membrane topology. Future biochemical and mutational analyses will be required to test this model and define the determinants governing SRP-dependent retargeting.

What causes translocating ribosomes to dissociate from the translocon? It is possible that ribosome dissociation occurs passively during longer periods of translocation, without the additional stabilization provided by TMD binding to the lateral gate. However, while this remains speculative, we favor a model where ribosome dissociation may not be passive but rather coordinated by an active mechanism, which could explain reduced dissociation rates observed after synthesizing marginally hydrophobic TMDs. The force exerted on the nascent chain during the lateral diffusion of more hydrophobic TMDs might dislocate the ribosome from the translocon, enhancing ribosome dissociation during synthesis of the proceeding loop. Luminal loops may trigger less dissociation since TMDs preceding these loops tend to be less hydrophobic. Additional factors influencing ribosome dissociation during cytosolic loop synthesis may include: (i) luminal chaperone binding, such as Kar2 (yeast BiP), and folding of luminal domains stabilizing ribosome proximity; (ii) interactions of cytosolic factors with cytosolic loops, potentially promoting dissociation; and (iii) conformational changes in the translocon during cytosolic loop synthesis, particularly those related to the relocalization of the plug helix, which seals the translocon during translation of cytosolic segments^72–74^.

It is intriguing to speculate that the plasticity of translocon interactions we report here may be critical for enabling a translocating nascent chain to transiently engage different membrane-embedded machineries for insertion or for chaperoning. In mammalian cells, the MPT can take over the insertion of TMDs with short intervening loops, concurrently blocking the lateral gate of the associated Sec61 translocon and restricting nascent chain access to it^22,62^. An unresolved aspect of this model is the translocation of long downstream luminal loops, which could be enabled by ribosome undocking from an MPT-associated translocon and transfer to an unblocked translocon. In *S. cerevisiae*, where the MPT is only partially conserved, the EMC may compensate for missing MPT components, participating in the insertion and chaperoning of nascent chains^22,66^. However, it may be sterically not possible for a nascent chain synthesized by a translocon-bound ribosome to engage the EMC unless the ribosome dissociates^75^. Supporting this, after the synthesis of the EMC-bound TMD clusters of top EMC substrates Fks1 and Pma1^66^, ribosomes were in a translocon-undocked state (Fig. 4f). As a picture emerges of unexpectedly dynamic ribosome interactions following initial targeting to the translocon, future studies will be needed to further elucidate how binding events are coordinated among the translocon, auxiliary membrane complexes, SRP, and cytosolic chaperones.

**Fig. 5:**
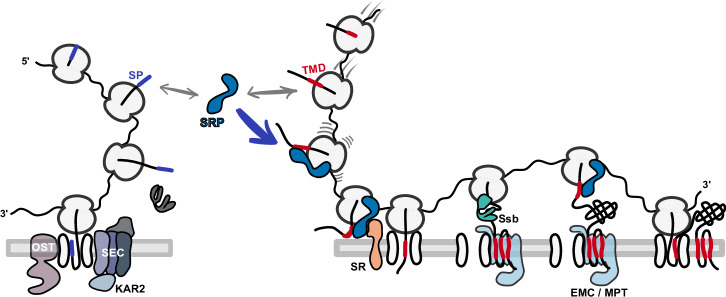
Model of triaging of ER-destined proteins in the cytosol, SRP mediated targeting, and retargeting of RNCs that have undocked from the translocon. SRP specifically binds targeting signals that contain a high number of hydrophobic amino acids after they emerge from the ribosome. SRP-bound signals include almost all N-terminal TMDs but only a fraction of SPs. The targeting signals rejected by SRP are targeted by an alternative cotranslational pathway to translocons associated with the SEC complex and OST. SRP may triage its substrates to translocons that are instead associated the SR and with complexes that assist the folding and insertion of membrane proteins, such as the EMC and/or the MPT. After translocon docking and TMD insertion, ribosomes often undock from the translocon, which allows chaperones like Ssb to assist the folding of cytosolic domains. In the case of multipass membrane proteins, SRP then rebinds the next emerging TMD and reestablishes the membrane contacts of the ribosome. Ribosome illustrations have been adapted from Koubek J, Schmitt J, Galmozzi CV and Kramer G (2021). Mechanisms of Cotranslational Protein Maturation in Bacteria. Front. Mol. Biosci. 8:689755. doi: 10.3389/fmolb.2021.689755 under a CC BY license: https://creativecommons.org/licenses/by/4.0/.

In summary, this study highlights the fundamental rules of SRP signal recognition in yeast cells and provides translatome-wide insights into targeting and translocation for proteins entering the secretory pathway (Fig. 5). Another key finding is the often overlooked extent of SRP-independent targeting of luminal and secreted proteins, which possibly extends to higher eukaryotes. This SRP-independent mechanism not only provides redundancy and alleviates SRP load but may also regulate downstream maturation steps by enabling early triaging decisions. Furthermore, the presented map of repetitive SRP interactions and alternating ribosome docking states at the translocon enhances our understanding of the interplay between the machineries that aid in the maturation of translocating nascent chains, particularly cytosolic chaperones and membrane-associated systems involved in enzymatic processing and maturation of nascent chains.

## Methods

### Strain construction

C-terminal GFP tagging was carried out as described previously^76^, whereby a monomeric GFP-KanMX4 cassette was inserted into the genomic locus via homologous recombination. The SRP54 gene was deleted by replacing the SRP54 gene with a NatNT2 cassette via homologous recombination. N-terminal seamless GFP-fusion strains were obtained from Maya Schuldiner^77^.

### Yeast growth analysis

1 mL of YPD media (1% yeast extract (BD), 2% peptone (BD), 2% glucose) was inoculated with overnight cultures at an OD600 of 0.2 in clear, flat-bottom 24-well plates. OD600 was measured through the course of 25 h using SPECTROStar Nano (BMG Labtech) with double orbital shaking at 30 °C.

### Yeast growth and cell harvest for ribosome profiling

Depending on the experiment, 200 mL or 1 L of YPD media was inoculated with overnight cultures at an OD600 of 0.03 and grown to early log phase (OD600 0.4–0.5) with shaking at 30 °C. Cells were harvested by rapid filtration through nitrocellulose membranes with a pore size of 0.4 µM, scraped with a metal spatula, and immediately frozen in liquid nitrogen. Frozen cell pellets were lysed together with frozen droplets of lysis buffer (600 µL for 200 mL culture, 900 µL for 1 L culture) using a mixer mill (2 min, 30 Hz, Retsch). The basic lysis buffer consisted of 20 mM Tris-HCl pH 8.0, 140 mM KCl, 10 mM MgCl_2_, 0.1% NP-40 (Sigma-Aldrich), 0.1 mg/mL Cycloheximide (Sigma-Aldrich), EDTA-free protease inhibitor tablet (Roche), 0.02 U/μl DNaseI (Roche), and 40 μg/mL Bestatin Hydrochloride (Roth). NP-40 was omitted for detergent-free lysis where indicated.

### Sample preparation for SeRP

For each SeRP replicate, 1 L yeast culture grown to early log phase (OD600 0.4–0.5) was harvested by rapid filtration as described before and lysed using 900 µL lysis buffer with detergent. Lysates were thawed for 2 min in a 30 °C water bath. Nucleic acid concentration was measured using NanoDrop and RNA digestion was carried out using 10 U RNase I (Ambion) per A_260 nm_ for 30 min at 4 °C. Digestion was stopped by chilling samples on ice and by adding 10 µL Superase-In (Thermo Fisher). Lysates were clarified by centrifugation at 500 × *g* for 5 min at 4 °C.

One fraction of the clarified lysate (corresponding to 800–1200 µg of total RNA) was used for SRP immunoprecipitation (IP). For the IP, 200 µL GFP-binder slurry^78^ was washed three times with 1 mL wash buffer (20 mM Tris-HCl pH 8.0, 140 mM KCl, 10 mM MgCl_2_, 10% glycerol, 0.01% NP-40, 0.1 mg/mL Cycloheximide, EDTA-free protease inhibitor tablet (Roche), 40 µg/mL Bestatin) by cycles of resuspending in wash buffer, centrifuging for 2 min at 450 × *g* at 4 °C, and discarding the supernatant. The lysate was mixed with GFP-binder coupled beads and rotated at 4 °C for 30 min. Afterwards, the mixture was centrifuged at 450 × *g* for 1 min and the supernatant was removed. Beads were washed 1× with 1 mL lysis buffer (5 min) and 3× with 1 mL wash buffer (2 × 5 min and 1 × 15 min). The sample volume was adjusted to 700 µL with the addition of lysis buffer.

The remaining clarified lysate (100–200 µg of total RNA) was used to prepare the total translatome. The lysate was layered on a sucrose cushion (25% w/v sucrose, 20 mM Tris-HCl pH 8.0, 140 mM KCl, 10 mM MgCl_2_, 0.1 mg/mL Cycloheximide, 1× Roche EDTA-free complete PI tablets, 40 µg/mL Bestatin) and centrifuged for 90 min at 253,838 × *g*, (75,000 rpm) at 4 °C in an S120-AT2 rotor (Thermo Scientific). The supernatant was removed and the pellet was resuspended in a final volume of 700 µL of lysis buffer. RNA was extracted via Phenol-chloroform extraction from both this sample and the IP sample.

For detergent-free SRP-SeRP the experiment was performed as above, but by omitting detergents in the lysis buffer and the wash buffer.

### Detergent-free SRP-SeRP with chemical crosslinking

Yeast cultures harvested and mixer milled as above, by using a lysis buffer lacking detergent and containing HEPES instead of Tris (20 mM HEPES pH 7.5, 140 mM KCl, 10 mM MgCl_2_, 0.1 mg/mL Cycloheximide, EDTA-free protease inhibitor tablet, 0.02 U/μl DNaseI, and 40 μg/mL Bestatin). A 6 mM stock of BS^3^ (Thermo Scientific) was prepared in lysis buffer just prior to the experiment. The lysates were rapidly thawed in a small glass beaker containing 6 mM BS^3^, reaching a final concentration of 2 mM BS^3^. After a 30 min incubation on ice, BS^3^ was quenched by adding Glycine to a final concentration of 62.5 mM. RNase digestion and preparation of IP and total translatomes samples were carried out as above, throughout using lysis buffer and wash buffer containing HEPES and lacking Tris and detergent (Wash buffer: 20 mM HEPES pH 7.5, 140 mM KCl, 10 mM MgCl_2_, 10% glycerol, 0.1 mg/mL Cycloheximide, EDTA-free protease inhibitor tablet, 40 µg/mL Bestatin).

To assess crosslinking of SRP to nascent chains, samples prepared as above with or without BS^3^ addition were subjected to sucrose cushion centrifugation. The resuspended ribosomal pellet was analyzed by western blotting to detect crosslinking of GFP-Srp54 to nascent chains using a polyclonal rabbit anti-YFP/GFP antibody^79^ (1:5000 dilution). Contrast was adjusted for visualization of the western blot without introducing signal saturation for the BS^3^ treated sample.

### Testing association of Srp54 with RNCs in RNase treated lysates

To test whether the RNase I treatment causes loss of RNC-SRP association, lysates of GFP-SRP54 yeast strains were prepared as above and were clarified by centrifugation at 500 × *g* for 5 min at 4 °C. The lysates were then split into two, treating one half with 10 U RNase I per A_260 nm_ and omitting RNase I for the other half. Samples were incubated for 30 min at 4 °C, then transferred to ice. 10 µL Superase-In was added to the digested sample. Afterwards, samples were loaded on a sucrose cushion and ribosomes were pelleted as described above. The resuspended ribosomal pellets were used to run a western blot, detecting GFP-Srp54 using a polyclonal rabbit YFP/GFP antibody^79^ (1:5000 dilution) and Rpl25 as a loading control using a polyclonal rabbit RPL25 antibody (1:10,000 dilution, laboratory stock). Contrast was adjusted for visualization of the western blot without introducing signal saturation.

Sample preparation for SeRP of soluble polysomes For SRP-SeRP of soluble polysomes, 1 L culture per replicate was harvested and lysed using 900 µL lysis buffer without detergent. The lysate was centrifuged twice at 16,000 × *g* for 15 min, both times collecting the supernatant. NP-40 was added to a final concentration of 0.1% and RNA digest was carried out as above. Next, the sample was split into two and an IP and total translatome sample were prepared as described above.

### Sample preparation for soluble ribosome profiling

For each soluble-RP replicate, 200 mL of yeast culture was harvested and lysed using 600 µL lysis buffer without detergent. RNA digest was carried out as described above. The lysate was then split into two halves to generate the soluble translatome and the total translatome. For the soluble translatome sample, the lysate was centrifuged twice at 16,000 × *g* for 15 min, both times collecting the supernatant. For the total translatome sample, NP-40 was added to the lysate to a final concentration of 0.1%, incubated for 5 min on ice, and then centrifuged at 500 × *g* for 5 min at 4 °C. The supernatant from this sample, as well as the final supernatant of the soluble translatome sample, were subjected to sucrose cushion centrifugation and pelleted ribosomes were resuspended as described before.

### Sample preparation for soluble-RP with PK treatment

Lyophilized PK from Tritirachium album was prepared as a 10 mg/mL stock in PK-storage buffer (50 mM Tris pH 7.5, 5 mM CaCl2, 40% glycerol). Aliquots were stored at −80 °C and thawed just prior to use.

1 L of yeast culture was harvested by filtration and lysed with 900 µL lysis buffer without detergent. The protease inhibitor tablet was omitted from the lysis buffer and instead E64 (1 µg/mL), Leupeptin (5 µg/mL), Pepstatin (8 µg/mL), and Bestatin (40 µg/mL) were added (PK-lysis buffer). After thawing, the protein concentration in the lysate was measured using the Bio-Rad protein assay. The RNA digest was carried out as above, and then the sample was split into four equal parts. Serial dilutions of PK in PK-lysis buffer were prepared and PK was added to each sample at a concentration ratio of 1:200, 1:2.000, or 1:20.000 (PK to total protein), or omitted. Samples were incubated at 4 °C for 30 min. PK was inhibited by addition of PMSF to a final concentration of 1 mM. From each sample, a soluble translatome and a total translatome sample were prepared as described above.

### Sucrose gradients and collection of collided disomes

Yeast cell lysates were prepared and RNA digestion was carried out as for SeRP. To form the sucrose gradients, two sucrose solutions were prepared containing 5% or 45% (wt/v) of sucrose, 20 mM Tris-HCl pH 8.0, 10 mM MgCl_2_, 140 mM KCl, 0.1 mg/mL Cycloheximide, and EDTA-free protease inhibitor tablet (Roche). Linear sucrose gradients were prepared by underlying the 5% sucrose solution with the 45% sucrose solution in SW40 centrifuge tubes (SETON), using short caps. Gradient Master (Biocomp) was used to mix the gradients with a custom 14-step program (M1: 09 s/83.0°/30 rpm, M2: 09 s/83.0°/0 rpm, M3: 01 s/86.0°/40 rpm, M4: 7 min/90.0°/0 rpm, with the sequence: 12121212121234). Gradients were cooled at 4 °C for at least one hour before overlaying them with the yeast cell lysates. For each replicate, two gradients were overlaid with the corresponding lysate containing 300 µg of RNA. Gradients were centrifuged in an SW40 Ti rotor for 2.5 h at 217,290 × *g* (35,000 rpm) at 4 °C. Gradients were fractionated using the Piston Gradient Fractionator (Biocomp) and disome fractions were collected using an automated fraction collector and combined before proceeding to phenol-chloroform extraction.

### RNA extraction and preparation of cDNA libraries

For all samples except for those from detergent-free SeRP experiments, phenol-chloroform extraction of RNA followed by cDNA library preparation was carried out as described previously^78^. For 30mer libraries, 2 and 5 nt long unique molecular identifiers (UMIs) were introduced to 5′ and 3′ ends of the reads, respectively as described. For 60mer libraries, 5 nt long UMIs were introduced to both 5′ and 3′ ends. For 30mer libraries, RNA fragments in the range of 20–40 nucleotides, and for 60mer libraries, RNA fragments in the range of 45–80 nucleotides were excised from 15% TBE-Urea gels as recovered as input for library preparation. Libraries were depleted for the most prevalent rRNA fragments using biotinylated custom oligos (developed in collaboration with siTOOLs Biotech) after the circularization step of library preparation, as previously described^29^. For detergent-free SeRP experiments, after excising RNA fragments in the range of 20–40 nucleotides, sequencing libraries were prepared using a protocol previously described^80^. For these samples, 10 and 5 nt long UMIs were introduced to 5′ and 3′ ends of the reads, respectively.

### Sequencing of cDNA libraries and data processing

The cDNA libraries were sequenced on a NextSeq550 (Illumina) according to the manufacturer’s instructions. Data was processed as described in ref. ^81^. More specifically, high-quality reads were trimmed using Cutadapt (v 2.8). A custom Julia script (Supplementary Software 1) was used to detect Unique Molecular Identifiers (Julia v 1.3.1). Noncoding RNA was removed using Bowtie (v 2.2.5) and genome alignment to *S. cerevisiae* S288C Genome (Assembly: GCA_000146045.2) was performed with STAR aligner (v 2.7.5a). Reads were assigned to the ribosomal P-site using Julia scripts provided as Supplementary Software 2 and 3 for 30 and 60 nt footprints, respectively. For analysis of 60 nt collided disome footprints, reads were assigned to the P-site of the leading ribosome. Majority of downstream analysis was carried out using the RP-data analysis package RiboSeqTools^82^ as well as custom R and Python Scripts.

### Annotation of protein classes

Proteins annotated to localize to plasma membrane, endoplasmic reticulum, extracellular, endoplasmic reticulum membrane, golgi membrane, or to golgi were classified as TMD, SP, or TMD and SP-containing based on UniProtKB^83^. The positions of TMDs or SPs as well as positions of loop regions and their topologies were obtained from UniProtKB for large-scale analysis. Targeting signals of individual genes used for single gene examples were annotated according to the consensus prediction from TOPCONS^84^. TMDs whose N-terminus falls within 50 amino acids distance to the stop codon were classified as tail-anchored (TA). Proteins annotated as mitochondrial either on UniProtKB or by Chartron et al., 2016 were classified as mitochondrial. Cytosolic proteins were classified as annotated previously^12^. All others were classified as “other”. Δ*G*_app_ values of TMDs annotated in UniProt were predicted using the web server https://dgpred.cbr.su.se/^51^. N-glycosylated proteins were categorized as annotated on UniProt. Annotation of Sec71-dependent SPs was obtained from ref. ^38^. The described annotations of the yeast proteome are provided in the Supplementary Data 1.

### Single gene profiles

All single gene profiles were generated using the R package RiboSeqTools. Single gene enrichment profiles are calculated by forming the ratio (IP/total translatome or soluble/total translatome) of the library size normalized P-site assigned read counts across codon positions. Values were smoothed with a 15 codon sliding window unless indicated otherwise. Position-wise enrichment confidence intervals (CI) were calculated because simply plotting position-wise enrichment can convey a false sense of precision, even though the value may have been calculated from only a few reads. At each position within the gene, read counts within a window size-wide neighborhood are summed up and used for CI calculation. The 95% CI according to Agresti and Coull^85^ of the enrichment ratio is calculated for each biological replicate separately. For single gene profiles showing the distribution of collided disomes, reads are assigned to the P-site of the leading ribosome and were library size normalized. Read counts within a window size-wide neighborhood are summed up and used for calculation of the 95% Poisson CI.

### Metagene profiles

Metagene profiles were generated using RiboSeqTools. They depict the averaged position-wise enrichment profiles (either IP/total translatome or soluble/total translatome) calculated after alignment of codon-resolved enrichment values from the indicated set of genes at a specified position. The shading indicates the 95% bootstrapping confidence interval. For metagene profiles showing the distribution of 30 nt total translatomes or 60 nt collided disome translatomes, the contribution of each gene was normalized to its expression level by dividing the read density at each codon position by the normalized read density of the gene (in RPKM).

### Statistical significance tests

For comparison of binding scores in Fig. 1e, f, Wilcoxon rank sum test was used with Holm–Bonferrini correction for multiple hypothesis testing. For comparison of N-glycosylation in Fig. 2F, pairwise Fisher test and to test for a significant difference in doubling times of different yeast strains, *T*-test was used. The adjusted p-values were indicated where significant, and those between 0.05 and 1 were indicated as not significant (ns).

### Determination of cotranslational targeting, targeting onsets, and targeting efficiencies

Cotranslationally targeted proteins were expected to form a sigmoidal total/soluble translatome profile. Proteins with the corresponding targeting profiles were detected using the sigmoid detection algorithm previously published^29^. Proteins with sufficient read coverage (read density > 0.4) and sigmoid detection results (sigmoidal or non-sigmoidal) for two replicates were used for downstream analysis. The targeting onsets were determined as the inflection point of the sigmoid. To calculate depletion efficiencies, ratios of library size normalized soluble translatome reads to total translatome reads along codon positions were calculated. The average of these values after the targeting onset was divided by that before the onset. This value was subtracted from 1 and multiplied by 100 to calculate the percent depletion efficiency.

Proteins detected as “non-sigmoidal” were annotated as “*not cotranslationally targeted”*. Furthermore, proteins detected as “sigmoidal” by the algorithm that had depletion efficiencies below 25% were excluded. Proteins with higher depletion efficiencies were annotated to be *cotranslationally targeted* (between 25 and 50%: low confidence, higher than 50%: high confidence).

To estimate the number of cotranslationally targeted proteins among all ER-targeted proteins in *S. cerevisiae*, the probability of cotranslational targeting was modeled as a function of protein length using logistic regression, with the R package brglm2^86^ using the *brglmfit* method. The fitted model was then applied to the full set of annotated ER proteins based on their length distribution, and predicted probabilities were averaged to estimate the fraction of those that are cotranslationally and posttranslationally targeted.

### SRP binding scores and annotation of SP-containing SRP substrates

SRP-binding scores were calculated using RiboSeqTools *binding_scores* function with a window size of 45 nucleotides. This score represents the lower end of the 95% confidence interval of SRP-enrichment (IP/total), at the codon position where this value is the highest for a given gene. The average binding score of two replicates were calculated. For more precise analysis of SRP binding to SP-containing proteins (used in Fig. 2C), proteins with a binding score higher than two, where the score position falls within the 150 codons, were bioinformatically filtered as potential SRP-substrates. The SRP-enrichment profiles of these genes were compared to the enrichment profiles detected by the mock-SeRP experiment^81^. The few genes where the SRP-enrichment profile was very similar to mock-SeRP were excluded and all others were annotated as SRP-binders. For comparison of depletion efficiencies for this analysis, three soluble-RP experiments performed in parallel were analyzed (WT replicate 2 and average of *SRP*Δ replicates 1 and 2). To calculate depletion efficiencies in *SRP*Δ strains, the average targeting onset detected in the WT strain was used (*n* = 2). Proteins that had more than 20% reduction in their targeting efficiency in the *SRP*Δ strain in comparison to WT were considered to require SRP for targeting. Finally, the 67 SP-containing proteins passing aforementioned read density and reproducibility requirements in experiments with WT and *SRP*Δ strains were annotated as *SRP-dependent* (SRP-binder and requires SRP for targeting), *SRP-facultative* (SRP-binder but does not require SRP for targeting), or *SRP-independent* (does not bind SRP and does not require SRP for targeting).

### Sequence logo analysis

For sequence logo analysis of nascent chain stretches recognized by SRP, statistically significant SRP binding positions were identified using the RiboSeqTools threshold-free peak detection approach using *fit_background_model, test_binding* (window_size = 45) and *get_binding_positions*. From the detected stretches of SRP-binding, the most N-terminal SRP-binding period for ER-targeted proteins that lasted longer than 2 codons were selected. Sequence stretches starting from the amino acid at the ribosomal P-site at the onset of SRP binding to 70 residues synthesized before were used to generate the sequence logo using the online tool: www.twosamplelogo.org^87^. Randomly picked sequence stretches of 70 amino acids from protein-coding sequences of *S. cerevisiae* were used as negative control for the analysis.

### Calculation of FILVW-PGS scores and comparison of predictive metrics of SRP binding

FILVW scores were calculated by summing up Phe, Ile, Leu, Val, and Trp residues in the hydrophobic region of SPs. Hydrophobic region positions for yeast and human SPs were obtained from the annotations of ref. ^10^ and ref. ^88^, respectively. PGS scores were calculated by summing up the number of Ser or Gly and Pro residues at the center of SP hydrophobic regions. For Ser, the central 4 and for Pro or Gly the central 6 residues were summed up. FILVW-PGS scores were calculated as the difference between FILVW and PGS scores.

The different metrics to predict SRP binding was were quantitatively compared using receiver operating characteristic analysis, in which sensitivity (true positive rate) and specificity (1—false positive rate) were evaluated across multiple thresholds and the resulting areas under the curve were compared.

### Analysis of Ssb interactions after cotranslational targeting

Statistically significant Ssb1 binding periods (> 9 codons) were detected as described for SRP above, using a previously published Ssb interactome dataset identified by SeRP^55^. To analyze Ssb interactions that occur after cotranslational targeting, the Ssb binding periods that start at least 50 codons after Soluble-RP detected cotranslational targeting onsets were used. To analyze Ssb interactions occurring after cotranslational targeting, only Ssb binding periods that began at least 50 codons downstream of the cotranslational targeting onset detected by Soluble-RP were considered.

The number of Ssb binding periods was compared among multipass membrane proteins with different topologies. These were categorized according to their soluble domains as follows:

Long, cytosolic: contain at least one cytosolic internal loop or C-terminal segment longer than 100 amino acids;

Long, luminal: contain at least one luminal internal loop or C-terminal segment longer than 100 amino acids and no long cytosolic loop;

Short: contain only short ( < 30 amino acids) internal loops and a short C-terminal segment.

### Reporting summary

Further information on research design is available in the Nature Portfolio Reporting Summary linked to this article.

## Supplementary information

Supplementary Information

Description of Additional Supplementary Files

Supplementary Data 1

Supplementary Data 2

Supplementary Data 3

Supplementary Data 4

Supplementary Software 1

Supplementary Software 2

Supplementary Software 3

Reporting Summary

Transparent Peer Review file

## Source data

Source data

## Data Availability

The ribosome profiling raw data and processed HDF5 files generated in this study have been deposited in the Gene Expression Omnibus (GEO) database under the accession code GSE295216 and are publicly available as of the date of publication. A minimum dataset, together with instructions for processing raw sequencing reads to obtain ribosomal P-site–assigned read counts (HDF5 format), is publicly available as Figshare repository entry 29542751. Source data are provided with this paper. Previously published data, which has been analyzed in this study, are available in the GEO database under the accession codes GSE74393, GSE93830, and GSE74393. Source data are provided with this paper.

## Code availability

Julia scripts used to identify UMIs and for the assignment of ribosomal P-site position on footprints is available in this paper’s supplemental information. Custom R scripts and any additional information required to reanalyze the data reported in this paper is available from G.K. upon request.
