# Supplementary material for: SRP orchestrates protein biogenesis beyond initial ER membrane targeting
Source: Nat Commun. 2026 Jun 16;17:5316. doi: 10.1038/s41467-026-74404-2 (PMC13272954; doi:10.1038/s41467-026-74404-2)
Supplement: Supplementary file 1 — Supplementary Information [file 41467_2026_74404_MOESM1_ESM.pdf]

# Supplementary Information

# SRP orchestrates protein biogenesis beyond initial ER membrane targeting

Ilgin Eser Kotan, Sabrina Sartori, Rudra Bose, Bernd Bukau*, Günter Kramer*

Supplementary Figures 1-8

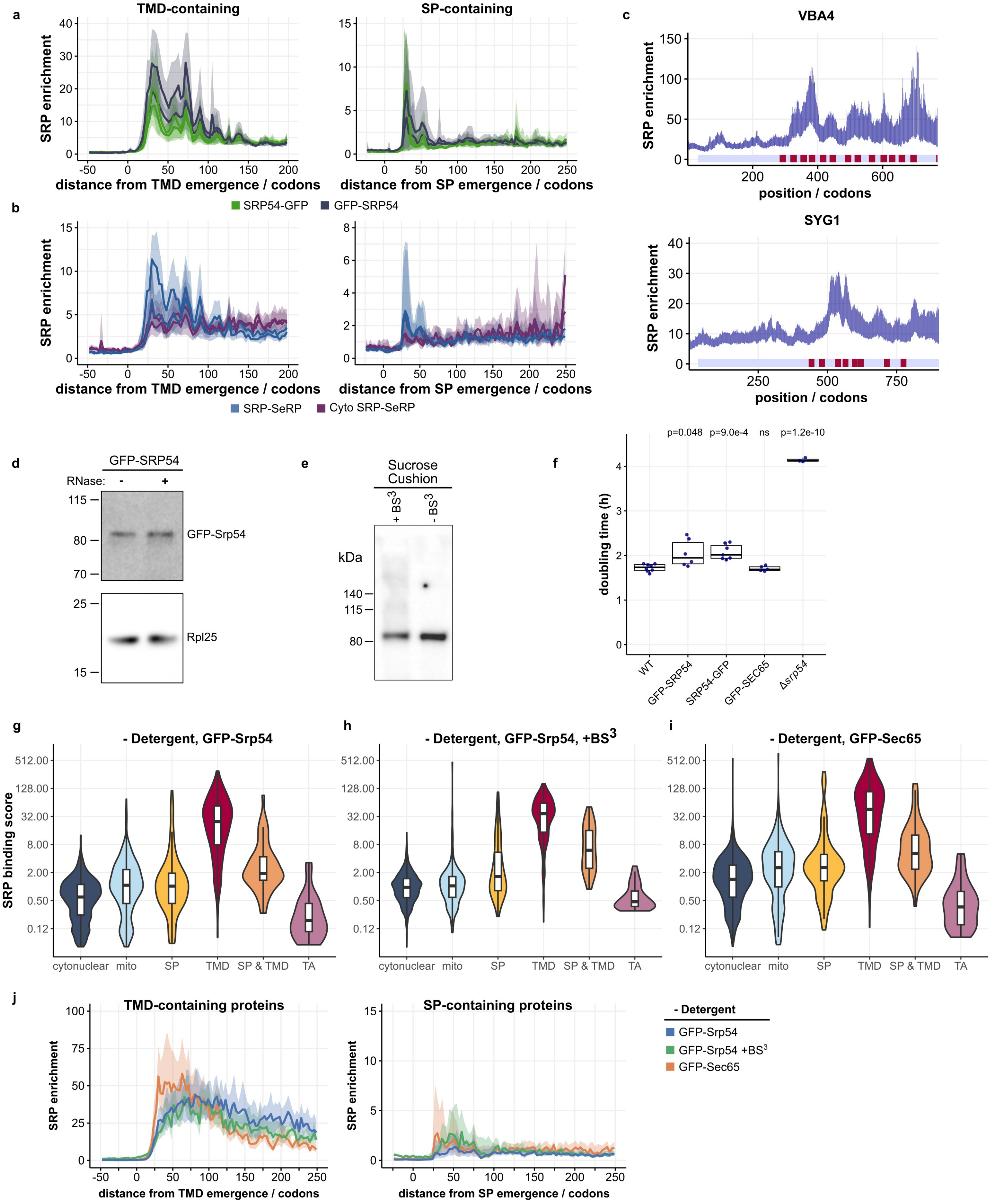

a
TMD-containing
SP-containing
SRP enrichment
distance from TMD emergence / codons
distance from SP emergence / codons
SRP54-GFP
GFP-SRP54
b
SRP-SeRP
Cyto SRP-SeRP
c
VBA4
SYG1
position / codons
d
GFP-SRP54
RNase:
GFP-Srp54
Rpl25
e
Sucrose Cushion
+ BS3
- BS3
kDa
f
p=0.048
p=9.0e-4
ns
p=1.2e-10
doubling time (h)
WT
GFP-SRP54
SRP54-GFP
GFP-SEC65
Δsrp54
g
- Detergent, GFP-Srp54
h
- Detergent, GFP-Srp54, +BS3
i
- Detergent, GFP-Sec65
SRP binding score
cytonuclear
mito
SP
TMD
SP & TMD
TA
j
TMD-containing proteins
SP-containing proteins
- Detergent
GFP-Srp54
GFP-Srp54 +BS3
GFP-Sec65

**Supplementary Figure 1. Binding preferences of SRP assessed using alternative SeRP approaches. a)** Metagene profiles of SRP enrichment determined by SRP-SeRP of strains encoding N- or C-terminally GFP-tagged Srp54 (blue and green respectively). Each line indicates a biological replicate (n=2). For this and all following metagene profiles, shaded areas indicate the 95% confidence interval derived by bootstrapping. **b)** Metagene profiles of SRP enrichment detected via SRP-SeRP using the cytosolic polysome fraction (purple) or a total lysate (blue). Each line indicates a biological replicate (n=2). **c)** SRP enrichment profiles of Vba4 and Syg1 calculated from SeRP datasets generated by Chartron et al., 2016. Ratio of normalized reads from SRP IP sample (from soluble polysomes without in vivo CHX treatment) to that in a total translatome sample (of soluble polysomes without in vivo CHX treatment) is plotted along the codon positions. **d)** To test the effect of RNase I treatment on the association of Srp54 with ribosomes, yeast lysates were incubated with or without RNaseI. The ribosome fraction was purified using a sucrose cushion and associated GFP-Srp54 was detected using a GFP antibody. Rpl25 was detected as a loading control. **e)** Lysates treated with or without the crosslinker $BS^3$ were used to perform a sucrose cushion centrifugation to purify RNCs. GFP-Srp54 crosslinks to nascent chains in these samples were assessed by western blotting using a GFP antibody. **f)** Doubling times of indicated yeast strains in log phase. Significant differences compared to the wild-type (WT) strain were assessed using a two-sided t-test. **g-i)** Comparison of SRP binding scores derived from detergent-free SeRP experiments using yeast strains encoding the indicated GFP-tagged SRP subunits (n=1). Experiments were performed either with or without $BS^3$ crosslinking. **j)** Metagene analysis of SRP interactions with TMD-containing or SP-containing proteins with or without detergent and $BS^3$ chemical crosslinking, based on SeRP using the indicated strains (orange: GFP-SEC65, blue: GFP-SRP54, green: GFP-SRP54 + $BS^3$, n=1).

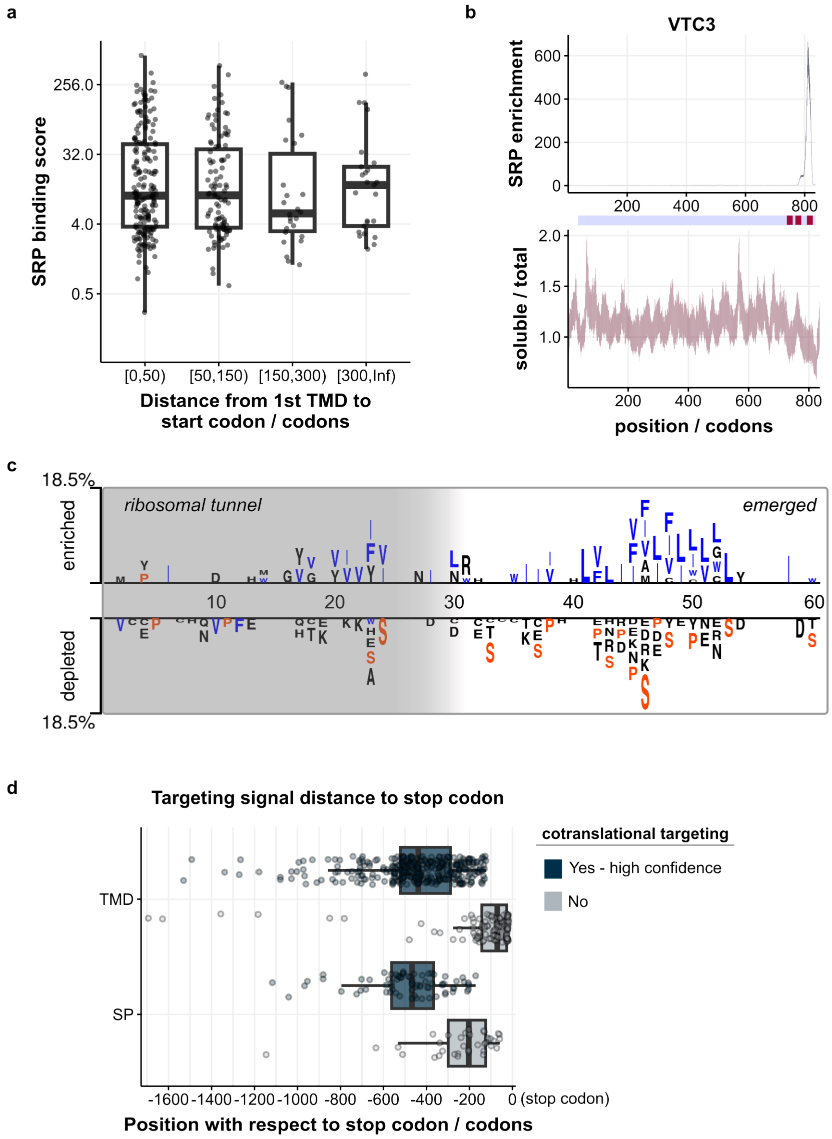


**Supplementary Figure 2. Determinants of SRP binding. a)** SRP binding scores of TMD-containing proteins grouped according to the distance of their first TMD-encoding sequence to the start codon. The average SRP binding scores derived from independent SeRP experiments are depicted (n=2). The median is indicated by the center line of the boxplot, the box represents the interquartile range (IQC), and whiskers extend to the most extreme values within 1.5X the IQC. **b)** SRP enrichment and targeting profiles of the ER membrane protein Vtc3. **c)** Sequence logo analysis of nascent chain segment synthesized shortly before SRP binding. Position 1 corresponds to the C-terminal amino acid at the start of SRP-binding. Hydrophobic residues are highlighted in blue, Serines and Prolines in red. **d)** The distance of the most N-terminal targeting signal (1st TMD or SP) to the stop codon. Proteins are binned according to their targeting signal (TMD or SP) and their targeting mode as detected by soluble-RP.

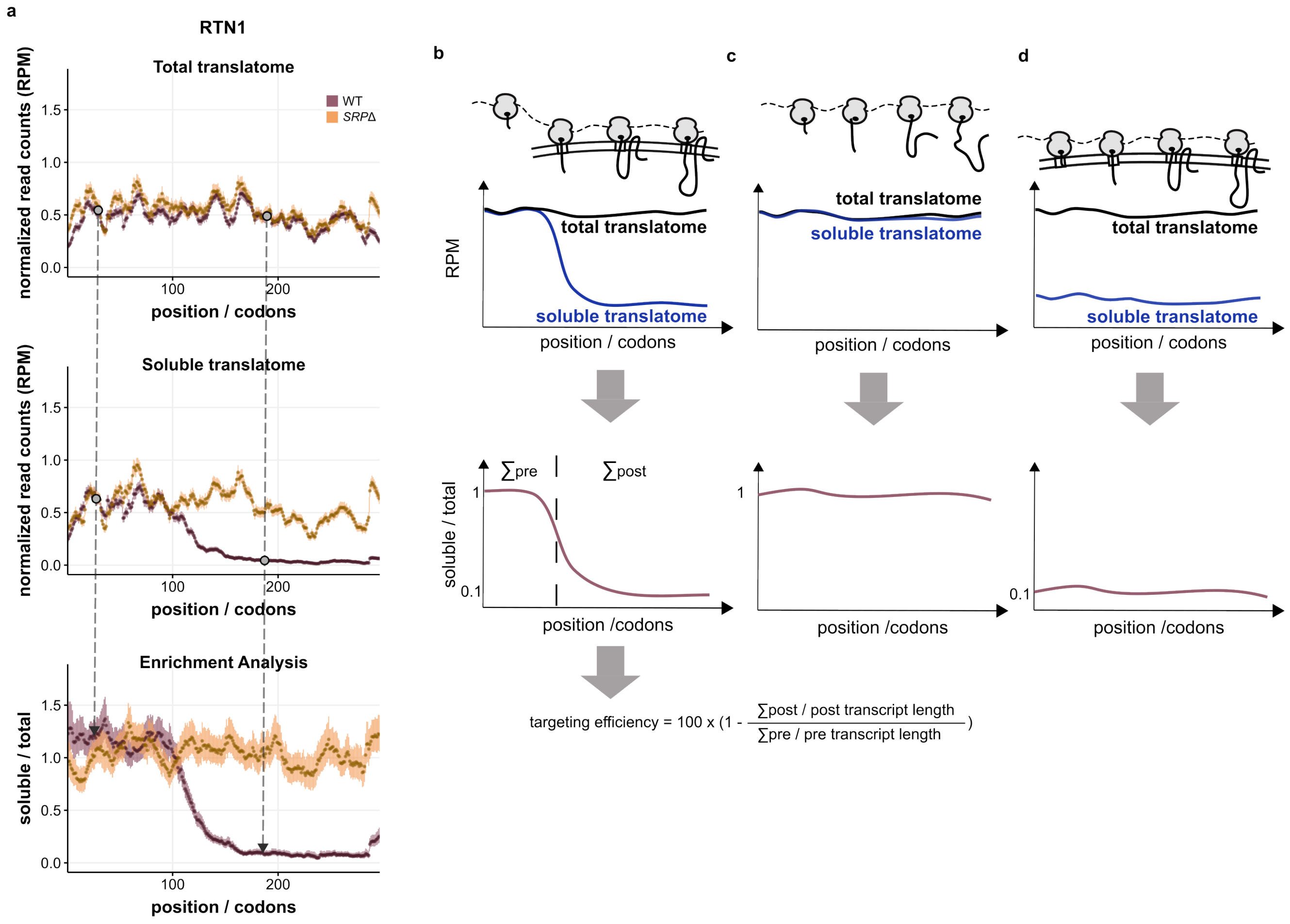


**Supplementary Figure 3. Data analysis of soluble-RP data. a)** Using RTN1 as an example, RPM values calculated at each position across this transcript in the total translatome (top panel) and the soluble translatome (middle panel) are depicted. The enrichment analysis (bottom panel) is performed by calculating the ratio of the RPM (Reads Per Million mapped reads) values in the soluble translatome over the total translatome for each codon position. The analysis was carried out for wild-type (WT, pink) and *SRP*Δ (orange) strains separately. **b)** The expected RPM distributions and soluble / total translatome profiles of a cotranslationally targeted protein, **c)** of a protein that is not cotranslationally targeted, or **d**) of a protein translated by ribosomes attached to the ER-membrane from the start of translation. Ribosome illustrations have been adapted from Koubek J, Schmitt J, Galmozzi CV and Kramer G (2021) Mechanisms of Cotranslational Protein Maturation in Bacteria. Front. Mol. Biosci. 8:689755. doi: 10.3389/fmolb.2021.689755 under a CC BY license: https://creativecommons.org/licenses/by/4.0/.

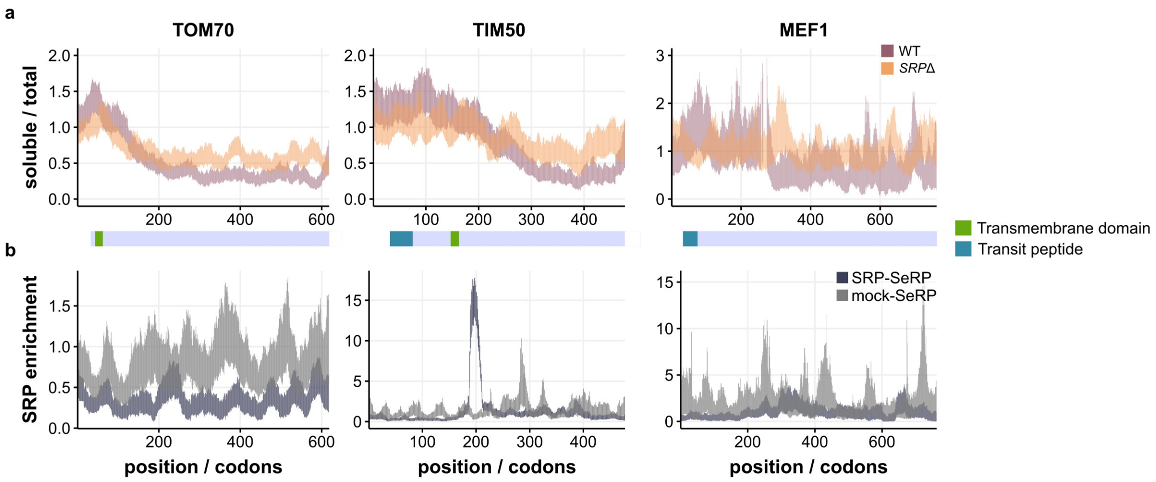


**Supplementary Figure 4. Cotranslationally targeted mitochondrial proteins identified using soluble-RP. a)** Targeting profiles of three proteins with mitochondrial localizations in WT (pink) and *SRP*Δ (orange) yeast strains. Transit peptide and TMD positions as annotated in Uniprot are depicted, shifted by 30 aa to account for their emergence from the exit tunnel **b)** SRP enrichment (blue) and mock-SeRP (gray) profiles of proteins in (a).

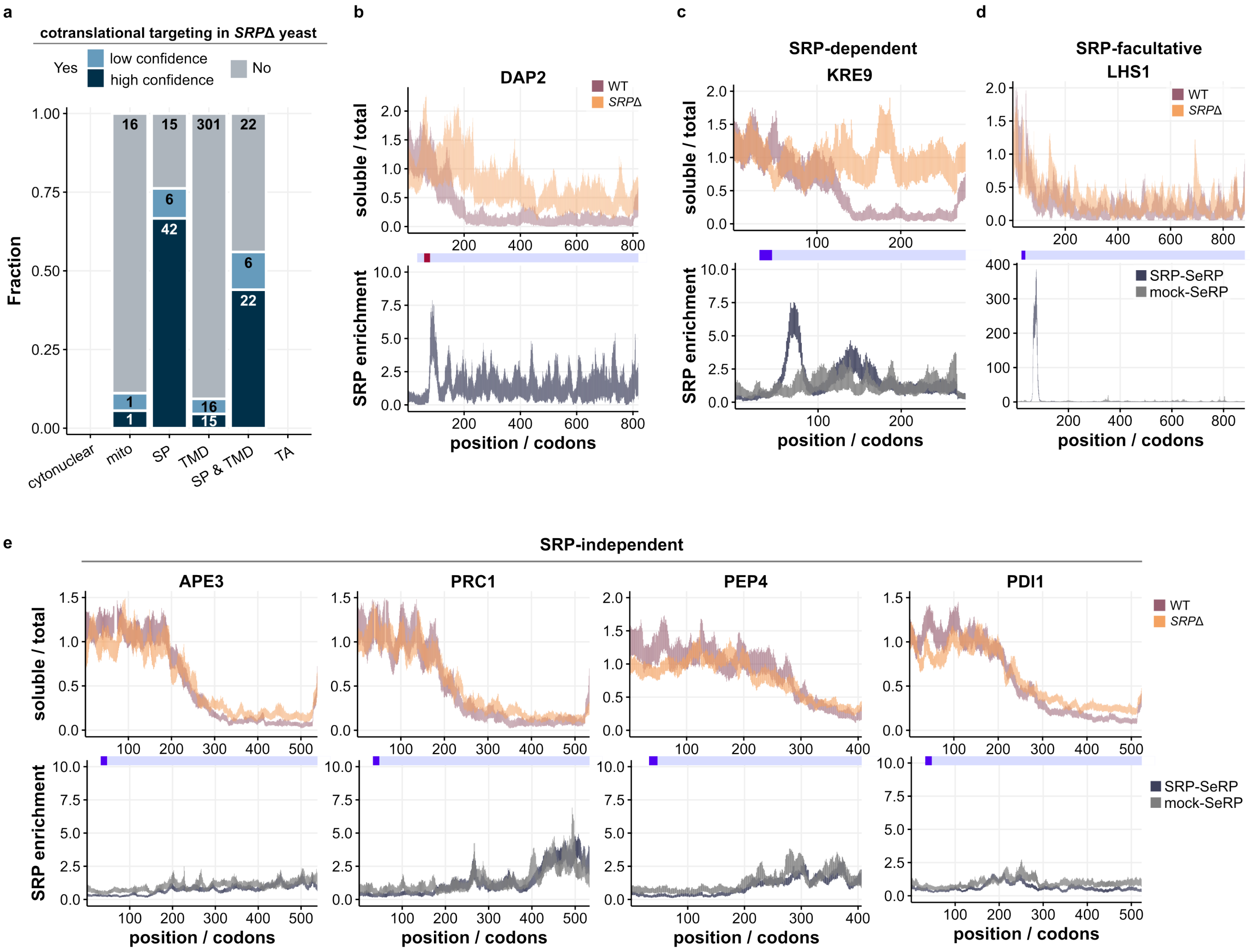


**Supplementary Figure 5. Cotranslational membrane targeting in an *SRP*Δ yeast strain**. **a)** Targeting modes of proteins from different localization categories that are cotranslationally targeted in wild-type (WT) yeast, assessed in *SRP*Δ strains by soluble-RP. Classification was based on sigmoid curve detection and percent reduction in normalized soluble reads after targeting onset: no (<25% or non-sigmoidal), yes-low confidence (25–50%), and yes-high confidence (≥50%). **b)** Targeting profile of the TMD-containing protein Dap2 in WT (pink) and *SRP*Δ (orange) strains (top) and its SRP enrichment profile (bottom). **c)** Single gene profiles of example SRP-dependent **d)** SRP-facultative **e)** SRP-independent proteins. Top panels: targeting in WT and *SRP*Δ yeast. Bottom: Enrichment values (IP/total translatome) from SRP-SeRP (blue) or mock-SeRP performed using an untagged strain (grey).

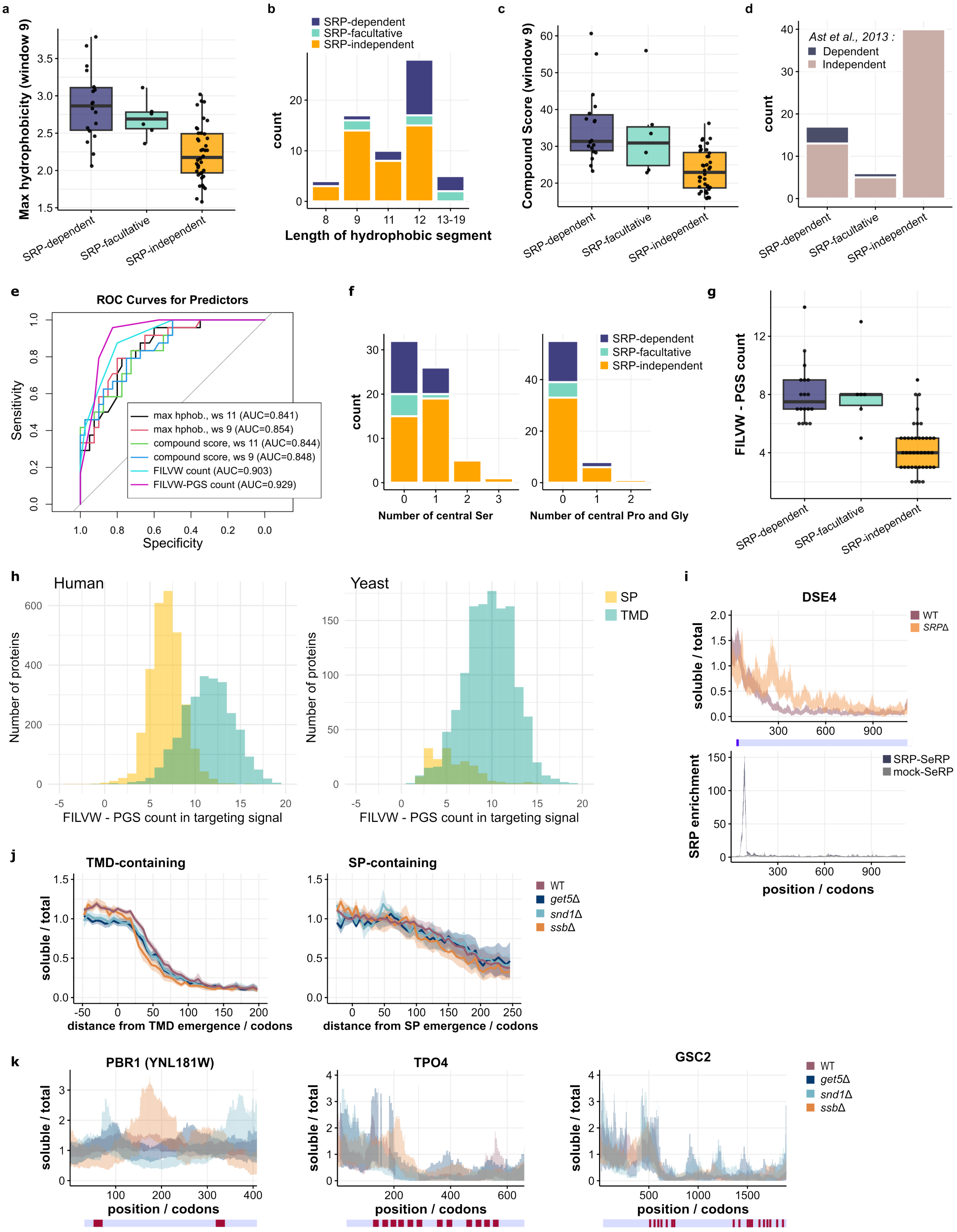

a
Max hydrophobicity (window 9)
SRP-dependent
SRP-facultative
SRP-independent
b
SRP-dependent
SRP-facultative
SRP-independent
count
Length of hydrophobic segment
c
Compound Score (window 9)
d
Ast et al., 2013 :
Dependent
Independent
count
e
ROC Curves for Predictors
Sensitivity
Specificity
max hphob., ws 11 (AUC=0.841)
max hphob., ws 9 (AUC=0.854)
compound score, ws 11 (AUC=0.844)
compound score, ws 9 (AUC=0.848)
FILVW count (AUC=0.903)
FILVW-PGS count (AUC=0.929)
f
count
Number of central Ser
Number of central Pro and Gly
g
FILVW - PGS count
h
Human
Yeast
SP
TMD
Number of proteins
FILVW - PGS count in targeting signal
i
DSE4
WT
SRPΔ
soluble / total
SRP-SeRP
mock-SeRP
SRP enrichment
position / codons
j
TMD-containing
SP-containing
WT
get5Δ
snd1Δ
ssbΔ
soluble / total
distance from TMD emergence / codons
distance from SP emergence / codons
k
PBR1 (YNL181W)
TPO4
GSC2
soluble / total
position / codons

**Supplementary Figure 6. Determinants SRP-dependency for targeting. a)** Maximum Kyte-Doolittle hydrophobicity of SPs (Ast et al., 2013) calculated using a sliding window of 9 aa in groups of proteins binned according to their SRP-pathway usage. **b)** Distribution of hydrophobic segment lengths of SPs compared to their SRP-pathway usage. **c)** Compound hydrophobicity scores of SPs (window size: 9) compared to their SRP-pathway usage, as calculated by Ast et al., 2013. **d)** SRP-dependence predictions of SP-containing proteins by Ast et al., 2013 compared to SRP-pathway usage identified in this study. **e)** Receiver operating characteristic (ROC) analysis comparing the predictive performance of each metric based on how well they classify experimentally determined SRP engagement across varying thresholds. AUC: Area Under the Curve. **f)** The number of Ser (left) or Gly and Pro (right) residues at the center of SP hydrophobic regions. For Ser, the central 4 and for Pro or Gly residues the central 6 residues were analyzed. **g)** Total count of Phe, Ile, Leu and Trp residues minus the number of central Pro, Gly, or Ser residues in hydrophobic region of SPs in each SRP-dependence category. **h)** The distribution of the FILVW-PGS scores of human and yeast TMDs (turquoise) and SPs (yellow). **i)** Targeting and SRP enrichment profiles of Dse4 (smoothing window=30 aa). **j)** Targeting metagene profiles of all SP- or TMD-containing proteins in WT (pink), *get5Δ* (dark blue), *snd1Δ* (light blue) and *ssbΔ* (orange) strains (n=1). **k)** Single gene targeting profiles of TMD-containing proteins, previously identified as SND substrates (Aviram et al., 2016).

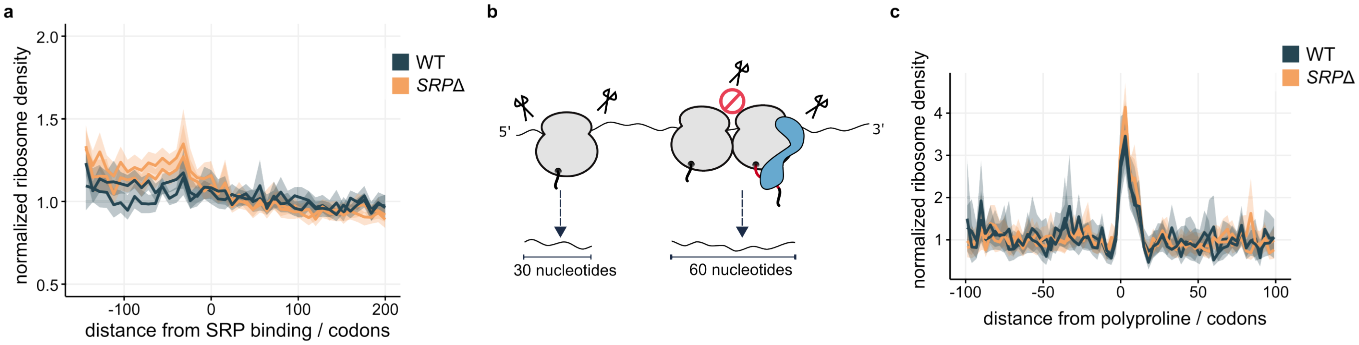


**Supplementary Figure 7. Profiling of collided disomes to study translation speed changes during SRP-mediated targeting. a)** Metagene profile showing the average distribution of 30 nt footprints on ER-targeted proteins in WT (dark blue) and *SRPΔ* (orange) yeast strains, aligned at the onset of the first SRP binding period detected for each gene. Each line indicates an independent biological replicate (n=2). **b)** RNase treatment of collided disomes generates ~60 nt footprint fragments instead of 30 nt fragments, which can be specifically size-selected and sequenced to study ribosome collisions via RP. **c)** Metagene analysis depicting the distribution of collided disomes along transcripts encoding polyproline stretches (≥3 prolines) in WT (dark blue) and *SRPΔ* (orange) yeast strains, aligned at the start of the polyproline stretch. Each line indicates an independent biological replicate (n=2). Ribosome illustrations have been adapted from Koubek J, Schmitt J, Galmozzi CV and Kramer G (2021) Mechanisms of Cotranslational Protein Maturation in Bacteria. Front. Mol. Biosci. 8:689755. doi: 10.3389/fmolb.2021.689755 under a CC BY license: https://creativecommons.org/licenses/by/4.0/.

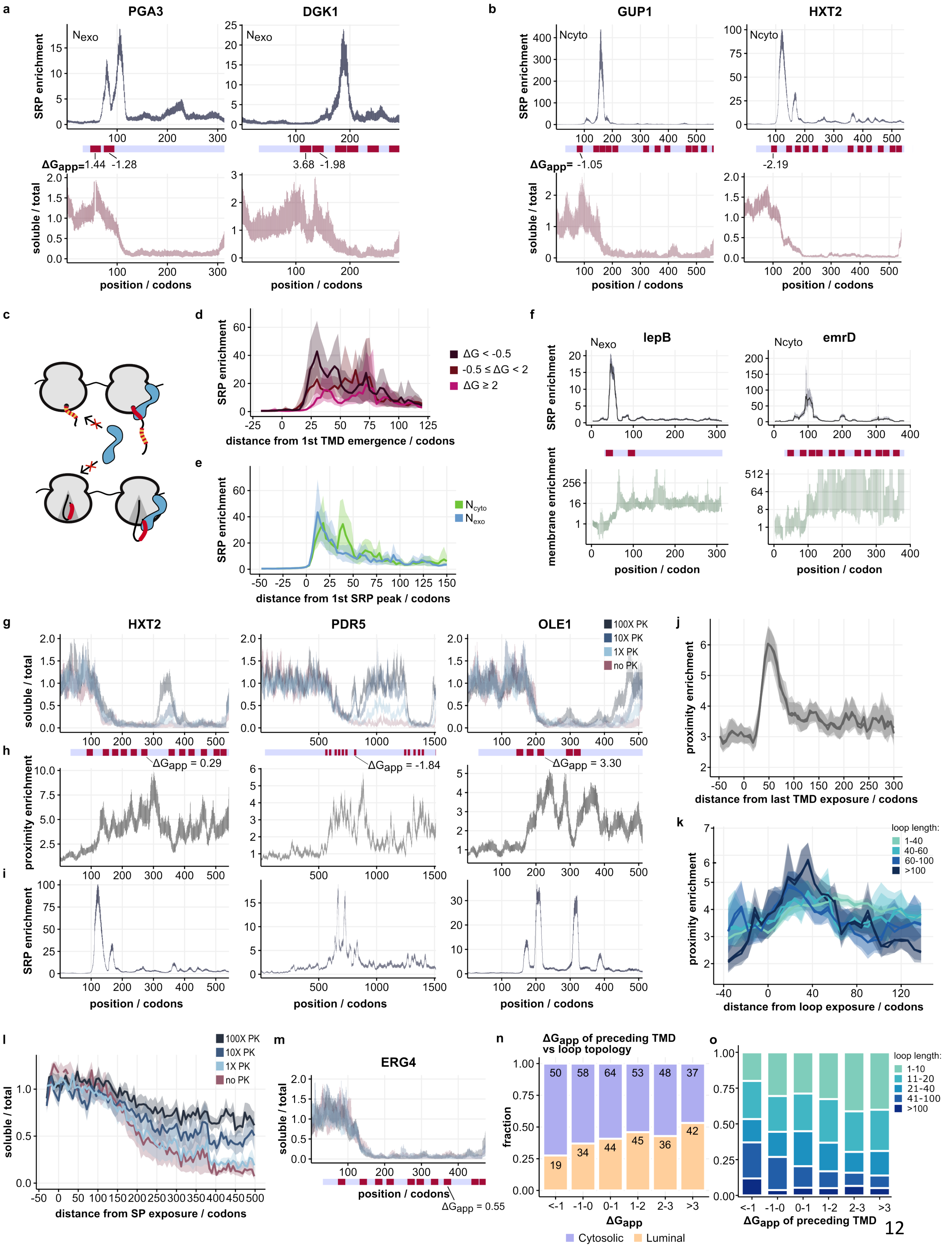
a
PGA3
DGK1
Nexo
SRP enrichment
ΔGapp=1.44 -1.28
3.68 -1.98
soluble / total
position / codons
b
GUP1
HXT2
Ncyto
ΔGapp= -1.05
-2.19
c
d
ΔG < -0.5
-0.5 ≤ ΔG < 2
ΔG ≥ 2
distance from 1st TMD emergence / codons
e
Ncyto
Nexo
distance from 1st SRP peak / codons
f
lepB
emrD
membrane enrichment
position / codon
g
HXT2
PDR5
OLE1
100X PK
10X PK
1X PK
no PK
h
ΔGapp = 0.29
ΔGapp = -1.84
ΔGapp = 3.30
proximity enrichment
i
j
distance from last TMD exposure / codons
k
loop length:
1-40
40-60
60-100
>100
distance from loop exposure / codons
l
distance from SP exposure / codons
m
ERG4
ΔGapp = 0.55
n
ΔGapp of preceding TMD vs loop topology
fraction
ΔGapp
Cytosolic
Luminal
o
loop length:
1-10
11-20
21-40
41-100
>100
ΔGapp of preceding TMD

**Supplementary Figure 8. Analysis of factors contributing to SRP binding to internal TMDs of a nascent chain. a-b)** SRP enrichment (top) and targeting (bottom) profiles of multipass membrane proteins. **c)** Models of delayed initial SRP engagement. Top: Marginally hydrophobic TMDs (yellow/red dashed) may escape SRP recognition during ongoing translation, allowing SRP to bind to a more C-terminal TMD (solid red). Bottom: Formation of a nascent chain loop in the ribosomal exit tunnel may delay TMD exposure to the cytoplasm and SRP binding. **d)** Metagene profiles comparing the average SRP enrichment of multipass membrane proteins based on the $\Delta G_{app}$ value of their first TMD, aligned at position where the first TMD emerges from the exit tunnel (n=1). **e)** Metagene profiles comparing the average SRP enrichment of multipass membrane proteins based on their topology, aligned at position of first detected SRP binding for each gene (n=1). **f)** SRP enrichment and membrane enrichment profiles of *E. coli* LepB and EmrD (Shibich et al., 2016, Eismann et al., 2022). Membrane enrichment profile depicts the relative enrichment of ribosome footprints in the membrane fraction in comparison to the total translatome. UniProt annotated targeting signals are depicted, their positions are shifted to account for the ribosomal exit tunnel. **g)** Single gene profiles of three multipass membrane proteins, based on soluble-RP with PK treatment. **h)** Ssh1-proximity specific ribosome profiling (IP/total translatome) (Jan et al., 2014), showing the RNC proximity to the ER-membrane protein Ssh1. **i)** SRP-SeRP enrichment profiles of indicated genes. **j)** Metagene profiles of Ssh1-proximity specific RP of all TMD-containing proteins, aligned at the position where the last TMD emerges from the exit tunnel (n=2). **k)** Metagene profiles of Ssh1-proximity specific RP of multipass membrane proteins, compared between internal loops of indicated lengths, aligned at the position where the loops start to emerge from the exit tunnel (n=2). **l)** Metagene profile of soluble-RP with PK treatment of SP-containing proteins, aligned at the position of SP-exposure (n=1). **m)** Soluble-RP with PK treatment profile of Erg4. **n)** Relative distribution of internal loop topology, compared to the $\Delta G_{app}$ values of the TMDs they proceed, for loops longer than 50 amino acids. **o)** Relative distribution of loop lengths compared to the $\Delta G_{app}$ values of the preceding TMD. Ribosome illustrations have been adapted from Koubek J, Schmitt J, Galmozzi CV and Kramer G (2021) Mechanisms of Cotranslational Protein Maturation in Bacteria. Front. Mol. Biosci. 8:689755. doi: 10.3389/fmolb.2021.689755 under a CC BY license: https://creativecommons.org/licenses/by/4.0/.
