# Supplementary material for: SRP orchestrates protein biogenesis beyond initial ER membrane targeting
Source: Nat Commun. 2026 Jun 16;17:5316. doi: 10.1038/s41467-026-74404-2 (PMC13272954; doi:10.1038/s41467-026-74404-2)
Supplement: Supplementary file 2 — Description of Additional Supplementary Files [file 41467_2026_74404_MOESM2_ESM.pdf]

Description of Additional Supplementary Files

File name: Supplementary Data 1
Description: General annotation file containing information about localization and targeting signals of each protein.

File name: Supplementary Data 2
Description: Targeting and SRP binding annotations generated in the study via SeRP and solubleRP. Includes all genes passing read coverage and reproducibility cutoffs.

File name: Supplementary Data 3
Description: SRP dependence classification of SP-containing proteins, respective SeRP and solubleRP measured values used for the analysis and analysed sequence properties.

File name: Supplementary Data 4
Description: Targeting and SRP binding annotations generated in the study via SeRP and solubleRP. Includes all genes.

File name: Supplementary Software 1
Description: Julia script that identifies UMIs on sequencing data following adaptor removal.

File name: Supplementary Software 2
Description: Julia script that assigns the position of ribosome's E-, P-, and A-sites on 30 nt footprints, generating HDF5 files for downstream analysis.

File name: Supplementary Software 3
Description: Julia script that assigns the position of the leading ribosome's E-, P-, and A-sites on 60 nt collided disome footprints, generating HDF5 files for downstream analysis.
