# Supplementary material for: SRP orchestrates protein biogenesis beyond initial ER membrane targeting
Source: Nat Commun. 2026 Jun 16;17:5316. doi: 10.1038/s41467-026-74404-2 (PMC13272954; doi:10.1038/s41467-026-74404-2)
Supplement: Supplementary file 10 — Reporting Summary [file 41467_2026_74404_MOESM10_ESM.pdf]

nature portfolio

Corresponding author(s): Günter Kramer
Bernd Bukau

Last updated by author(s): May 7, 2026

Reporting Summary

Nature Portfolio wishes to improve the reproducibility of the work that we publish. This form provides structure for consistency and transparency in reporting. For further information on Nature Portfolio policies, see our Editorial Policies and the Editorial Policy Checklist.

Statistics

For all statistical analyses, confirm that the following items are present in the figure legend, table legend, main text, or Methods section.

n/a	Confirmed	
☐	☒	The exact sample size (*n*) for each experimental group/condition, given as a discrete number and unit of measurement
☐	☒	A statement on whether measurements were taken from distinct samples or whether the same sample was measured repeatedly
☐	☒	The statistical test(s) used AND whether they are one- or two-sided<br>*Only common tests should be described solely by name; describe more complex techniques in the Methods section.*
☒	☐	A description of all covariates tested
☐	☒	A description of any assumptions or corrections, such as tests of normality and adjustment for multiple comparisons
☐	☒	A full description of the statistical parameters including central tendency (e.g. means) or other basic estimates (e.g. regression coefficient) AND variation (e.g. standard deviation) or associated estimates of uncertainty (e.g. confidence intervals)
☐	☒	For null hypothesis testing, the test statistic (e.g. *F*, *t*, *r*) with confidence intervals, effect sizes, degrees of freedom and *P* value noted<br>*Give P values as exact values whenever suitable.*
☒	☐	For Bayesian analysis, information on the choice of priors and Markov chain Monte Carlo settings
☒	☐	For hierarchical and complex designs, identification of the appropriate level for tests and full reporting of outcomes
☒	☐	Estimates of effect sizes (e.g. Cohen's *d*, Pearson's *r*), indicating how they were calculated

Our web collection on statistics for biologists contains articles on many of the points above.

Software and code

Policy information about availability of computer code

Data collection	Code was not used to collect data.
Data analysis	Data was processed as described (Minoia et al., 2024), trimming high quality reads using Cutadapt (v 2.8), identifying UMIs using custom Julia (v 1.3.1) scripts (Supplementary software 1), removing noncoding RNAs using Bowtie2 (v 2.2.5) and performing genome alignment with STAR aligner (v 2.7.5a). Reads were assigned to the ribosomal P site and PCR duplicates were collapsed based on UMIs using a custom Julia script (Supplementary software 2). For analysis of 60 nt collided disome footprints, reads were assigned to the P site of the leading ribosome (Supplementary software 3). Downstream analysis was carried out using the RP data analysis package RiboSeqTools (ilia kats, 2020) available on GitHub.

For manuscripts utilizing custom algorithms or software that are central to the research but not yet described in published literature, software must be made available to editors and reviewers. We strongly encourage code deposition in a community repository (e.g. GitHub). See the Nature Portfolio guidelines for submitting code & software for further information.

Data

Policy information about availability of data

All manuscripts must include a data availability statement. This statement should provide the following information, where applicable:

- Accession codes, unique identifiers, or web links for publicly available datasets
- A description of any restrictions on data availability
- For clinical datasets or third party data, please ensure that the statement adheres to our policy

The ribosome profiling raw data and processed HDF5 files generated in this study have been deposited in the Gene Expression Omnibus (GEO) database under the accession code GSE295216 [https://www.ncbi.nlm.nih.gov/geo/query/acc.cgi?acc=GSE295216] and are publicly available as of the date of publication. A minimum dataset, together with instructions for processing raw sequencing reads to obtain ribosomal P-site–assigned read counts (HDF5 format), is publicly available at https://doi.org/10.6084/m9.figshare.29542751. Source data are provided with this paper. Previously published data which has been analyzed in this study are available in the GEO database under the accession codes GSE74393 [https://www.ncbi.nlm.nih.gov/geo/query/acc.cgi?acc=GSE74393], GSE93830 [https://www.ncbi.nlm.nih.gov/geo/query/acc.cgi?acc=GSE93830] and GSE74393 [https://www.ncbi.nlm.nih.gov/geo/query/acc.cgi?acc=GSE74393].

Research involving human participants, their data, or biological material

Policy information about studies with human participants or human data. See also policy information about sex, gender (identity/presentation), and sexual orientation and race, ethnicity and racism.

Reporting on sex and gender	This study was performed on the eukaryotic model organism budding yeast and did not involve human participants. Therefore findings do not apply to one sex or gender.
Reporting on race, ethnicity, or other socially relevant groupings	This study was performed on the eukaryotic model organism budding yeast and did not involve human participants. Therefore race, ethnicity or other socially relevant groupings were not variables in this study.
Population characteristics	This study was performed on the eukaryotic model organism budding yeast and did not involve human participants.
Recruitment	This study was performed on the eukaryotic model organism budding yeast and did not involve human participants.
Ethics oversight	This study was performed on the eukaryotic model organism budding yeast and did not involve human participants.

Note that full information on the approval of the study protocol must also be provided in the manuscript.

Field-specific reporting

Please select the one below that is the best fit for your research. If you are not sure, read the appropriate sections before making your selection.

☒ Life sciences ☐ Behavioural & social sciences ☐ Ecological, evolutionary & environmental sciences

For a reference copy of the document with all sections, see nature.com/documents/nr-reporting-summary-flat.pdf

Life sciences study design

All studies must disclose on these points even when the disclosure is negative.

Sample size	Typically ribosome profiling libraries were sequenced obtaining roughly 10-20 million reads for total translatome samples, which provides sufficient read coverage of all moderately expressed genes in the yeast proteome.
Data exclusions	No data was excluded from the study
Replication	For all major ribosome profiling experiments, two replicates were prepared as indicated in the figure legends (n). Replicates were prepared starting from the growth of two independent yeast cell cultures. All attempts at replication were successful.
Randomization	Randomization was not carried out as the study is purely experimental, uses genetically defined yeast strains and the analysis is not based on a subjective assessment of the results.
Blinding	Blinding was not carried out as the study is purely experimental, uses genetically defined yeast strains and the analysis is not based on a subjective assessment of the results.

Reporting for specific materials, systems and methods

We require information from authors about some types of materials, experimental systems and methods used in many studies. Here, indicate whether each material, system or method listed is relevant to your study. If you are not sure if a list item applies to your research, read the appropriate section before selecting a response.

Materials & experimental systems

n/a	Involved in the study
☐	☒ Antibodies
☒	☐ Eukaryotic cell lines
☒	☐ Palaeontology and archaeology
☒	☐ Animals and other organisms
☒	☐ Clinical data
☒	☐ Dual use research of concern
☒	☐ Plants

Methods

n/a	Involved in the study
☒	☐ ChIP-seq
☒	☐ Flow cytometry
☒	☐ MRI-based neuroimaging

Antibodies

Antibodies used

Polyclonal rabbit GFP antibody (antiserum from rabbit raised against YFP) (Haslberger, T. NSMB 15(6):641-50 (2008). 1:5000 dilution.
Polyclonal rabbit RPL25 antibody (antiserum from rabbit raised against yeast RPL25, 1:10,000), from laboratory stock.

Validation

GFP antibody was validated in a previous publication by Haslberger et al., NSMB 15(6):641-50 (2008) and by detection of GFP tagged Srp54 protein by western blotting at the expected size using western blotting in this study.

Plants

Seed stocks

This study was performed on the eukaryotic model organism budding yeast and plants were not used.

Novel plant genotypes

See above.

Authentication

See above.
