# Supplementary material for: SRP orchestrates protein biogenesis beyond initial ER membrane targeting
Source: Nat Commun. 2026 Jun 16;17:5316. doi: 10.1038/s41467-026-74404-2 (PMC13272954; doi:10.1038/s41467-026-74404-2)
Supplement: Supplementary file 11 — Transparent Peer Review file [file 41467_2026_74404_MOESM11_ESM.pdf]

nature portfolio

Peer Review File

SRP orchestrates protein biogenesis beyond initial ER membrane targeting

Corresponding Author: Dr Günter Kramer

This file contains all reviewer reports in order by version, followed by all author rebuttals in order by version.

Version 0:

Reviewer comments:

Reviewer #1

(Remarks to the Author)
Kotan et al. employed selective ribosome profiling (SeRP) to examine SRP interactions with nascent chains in yeast. Through careful execution of their experiments, the authors clarify some contentious interpretations from earlier studies and provide new insights into SRP-mediated protein biogenesis. A surprising finding is that most signal peptide-containing secretory proteins are targeted to the ER via an SRP-independent but co-translational pathway, whereas transmembrane proteins strictly rely on SRP-mediated co-translational targeting. Another key observation is that SRP appears to participate in repeated targeting of transmembrane domains (TMDs) of membrane proteins at the ER membrane. A potential concern is that the study relies primarily on a single technique without biochemical validation for some key findings. Nevertheless, the dataset is rich and provides valuable new insights into SRP's role in protein targeting in yeast.

Major Comments:
The authors appear to assume that yeast SRP and mammalian SRP behave similarly. Previous studies show that mammalian SRP can target a broad range of signal peptide-containing substrates. By contrast, the Sec61 translocon in mammals is more discriminatory, preferentially recognizing strongly hydrophobic signal peptides and often requiring additional translocon-associated factors such as TRAP and Sec63. It is possible that yeast SRP preferentially recognizes highly hydrophobic signals, such as TMDs, which may explain why most secretory proteins in yeast are co-translationally targeted in an SRP-independent but Sec71-dependent manner. Since mammals lack Sec71 and Sec72, SRP may have evolved to capture both weak and strong hydrophobic targeting signals. The authors should discuss these evolutionary and mechanistic considerations.
It is possible that SRP interactions with signal peptides of secretory proteins are weaker and thus not preserved during SRP-selective enrichment. Incorporating chemical or photo-crosslinking approaches could help address this possibility.
While the ribosome profiling data are powerful, the manuscript could be strengthened by biochemical validation, particularly regarding the intriguing finding that SRP binds TMDs of membrane proteins even after ER targeting. The reviewer understands that biochemical validation may be technically challenging, but including such data or discussing potential approaches would improve the manuscript.

Minor Comments:
Line 258: The authors suggest that SRP skipping the first TMD in some transmembrane proteins may be due to TMD looping inside the exit tunnel. While this is plausible, there is no direct biochemical or structural evidence presented. It should be framed as one possible explanation rather than a definitive mechanism.

(Remarks on code availability)

Reviewer #2

(Remarks to the Author)
This study uses ribosome profiling of SRP-affinity-purified polysomes and polysomes generated by fractionation to investigate the role of SRP during secretory and membrane protein synthesis in yeast. The authors make a number of observations that lead them to several conclusions, some of which match current models and others that don't. Their main

conclusions are that SRP preferentially binds to signal peptides (SPs) and transmembrane domains (TMDs) of high hydrophobicity, that it is recruited only once the SP or TMD has emerged from the ribosome, that the first TMD of a multipass membrane protein is sometimes 'skipped' by SRP, and that ribosomes detach during loop synthesis, with SRP being used to re-target these ribosomes when the next TMD emerges. They further conclude that SPs that don't engage SRP can nonetheless mediate co-translational targeting.

The datasets and analyses of such ribosome profiling studies are interesting and valuable to the field because they provide a global view of biochemical processes using a range of endogenous substrates and complement more focused biochemical and structural studies. However, the study as currently presented suffers from three general problems that would need to be addressed before publication. First, it uses only profiling methods with no attempt at corroboration of the more provocative or new conclusions with any orthogonal method or with more directed experiments. Second, the impaired growth of tagged SRP strains and the use of detergent in the SRP pulldowns may confound the results, leaving open quite plausible alternative interpretations of some of the key data. Third, there are a number of over-interpretations that go beyond what is justified by the data. Rectifying these problems is likely to require major revisions.

Major points:

1) The use of detergent in most of the experiments and in all of the SRP pulldowns has the potential to disrupt interactions between the SRP54 M domain and hydrophobic peptides. Even very small amounts of detergent (0.01%) have been shown to bind the hydrophobic groove of the M domain and mimic a signal peptide (see PMID 19119234), density in the M domain groove in the SRP54 crystal structure might have been detergent, which was required to obtain well-diffracting crystals (PMID 9695947), and detergent disrupts the analogous interaction between GET3 and its TMD substrate (PMID 17382883). Because the signal-SRP interaction is a major contributor to SRP-ribosome interactions, perturbing this may well influence what is recovered. In fact, the preference of SRP for substrates of high hydrophobicity can be readily re-interpreted as these being the least sensitive to detergent. If so, then many of the major conclusions in the paper have the potential to be incorrect or incomplete. The authors should most certainly be clear in the paper about these caveats so a reader can properly judge the study, and should ideally demonstrate in more directed experiments that yeast SRP has a strong preference for high-hydrophobicity SPs and TMDs. This could be done by in vitro interaction or crosslinking analyses (very common in the field), or if they really wanted to probe the situation in cells, by site-specific photo-crosslinking via probes in the SP or TMD.

2) The authors seem unconcerned by the slower growth of their tagged SRP54 strains. However, the doubling time seems to increase by ~20 to 30 min, which is non-trivial and indicates a substantial fitness cost of tagging SRP54. Because the basis of this fitness cost is unknown, it is hard to determine whether it affects any of the results or not. For example, if steric issues partially obscure SRP54 access to substrates, it could wind up being stable only on the most hydrophobic ones. This too is an alternative explanation for its observed specificity in the profiling. Obviously, tagging a subunit that is not involved in substrate recognition, taking advantage of the available structural information, would have been preferable. The N-terminus of SRP19, for example, seems flexible and protrudes away from the important functional parts of SRP. Again, acknowledging these caveats at a minimum is needed, but ideally, one would verify the results using a tagging strategy that does not impair fitness and can be more confidently verified to be fully functional.

3) The conclusion that SRP skips some TMDs and instead binds downstream TMDs is intriguing, but not especially convincing given the above two caveats. It is supported modestly by the parallel timing of membrane association, but this is on individual mRNAs with somewhat noisy data and doesn't always fit the authors' interpratation. For example, membrane targeting seems almost complete before the onset of SRP binding in the case of DGK1, so it doesn't seem like the major SRP binding event is responsible for targeting. Furthermore, the example of ERJ5 seems to indicate that something odd is happening here and that downstream binding might be some odd artefact. In ERJ5, the signal would mediate targeting and initiate translocation of the N-terminus. The fractionation supports targeting of the majority of nascent chains shortly after the signal emerges but before the TMD is synthesized. So the ribosome would be docked on Sec61 and in the process of translocating the N-terminal soluble domain. According to pretty well-established principles, the TMD would then emerge from the ribosome into Sec61, then partition laterally into the bilayer. So when exactly in this scheme is SRP purported to bind the TMD? Even if the idea is that the signal fails to be recognised and targeting does not happen until the TMD emerges, how would SRP binding to the TMD and targeting to the ER result in a protein of the correct topology? Given this odd result, one must wonder whether the 'skipping' of TMDs and binding downstream TMDs in other examples is a valid result. This can be resolved by directed experiments that analyse SRP binding to different length nascent chains in vitro for a substrate where the profiling suggests TMD1 is skipped and TMD2 is engaged.

4) The loop formation inside the ribosome being the basis of late SRP binding is not well supported for a number of reasons. First, the example of EmrE might simply reflect the fact that TMD1 is not as hydrophobic as TMD2. Second, many Ncyto signal anchors are preceded by long segments of polypeptide, so cannot sterically form a loop. Third, the one situation where a hydrophobic domain is necessarily at the extreme N-terminus and is certainly inserted into the membrane as a loop is a cleavable SP. Yet considerable biochemical data using crosslinking with nascent chains of defined lengths indicate that SRP binds immediately after the SP emerges. In the authors own profiling data, binding of SRP to SPs is consistent with binding as it emerges, not delayed due to loop formation. Thus, the loop idea seems very unlikely to be generally true and is highly speculative. If the authors want to propose this, they would need a more convincing argument.

5) The authors conclude on the basis of the flat soluble:total ratio (Fig. 2A) that membrane proteins are targeted post-translationally in ΔSRP cells. This seems highly unlikely as there is really no good paradigm for post-translational membrane protein insertion at the ER under any circumstances, except for tail-anchored proteins. I am not sure how the soluble:total ratio is calculated or normalised to 1.0, but if the ratio is unchanged, there seem to be two explanations: either

the mRNA is mostly in the cytosol and stays there, or is mostly at the membrane and stays there. Of the two, the latter makes more biological sense. Is there a way to have a more quantitative and non-normalised assessment of mRNA location in WT versus ΔSRP cells to clarify this issue? In the authors' model, membrane protein mRNA should be mostly membrane associated in WT cells, but cytosolic in ΔSRP cells. This can be easily tested.

6) The conclusion that " many multipass membrane proteins require repeated SRP action for efficient insertion of all TMDs" is very speculative given that there is no functional data that SRP is required beyond the initial targeting step, or that in the absence of a putative SRP re-targeting event, downstream TMDs are not inserted. Furthermore, while the profiling data for some substrates like PHO88 and PUN1 seem to support the model, others do not. For example, HXT2 shows substantial undocking, but a barely discernible SRP re-binding (at least 10-fold lower than the initial binding). The same is true for PDR5. For OLE1, SRP re-binding is robust, but there is barely any ribosome undocking. One therefore wonders whether cherry-picked examples are used to propose an intriguing model that may not be generally applicable.

7) The claim that cytosolic loops result in preferential ribosome undocking is not very strongly supported given that luminal loops also result in substantial undocking (Fig. 4E). This is a bit worrisome concerning the interpretation of the undocking data because translocation of luminal domains is pretty well established to occur through Sec61 via a docked ribosome. Furthermore, even if ribosomes did detach occasionally, the nascent chain would not easily slip back due to a combination of folding and chaperone binding on the luminal side. Finally, if the idea is that ribosomes detach during luminal domain translocation, then detachment should be seen during secretory protein translocation, or during Ncyto single-pass protein translocation. For these reasons, the conclusion that ribosomes undock during cytosolic loop production, while attractive, is not especially strongly supported by the available data.

8) The Ssb interaction is an interesting alternative way to support the ribosome undocking idea, but is shown for two somewhat noisy examples. Is there any meta analysis of this to provide stronger support for the idea?

9) SRP RNA is known to be sensitive to nuclease digestion at the hinge between the S domain and Alu domain. Do the authors know whether their RNase digestion conditions split SRP into these two domains? This is important because it may impact the stability of SRP binding to the ribosome, especially in the presence of detergent.

Minor points:

1) It is important to be clear throughout that the data apply to yeast and that the degree to which it can be extrapolated to other systems (e.g., mammalian) is sometimes unclear. The SRP system is of course exceptionally well conserved, but there are nonetheless major differences in targeting. The main one is that as far as we know, the mammalian system is capable of post-translational translocation of only a small set of special substrates (namely very small secretory proteins - see PMID 22648169 & 22196732). Even substrates such as yeast alpha-factor, which can be post-translationally translocated efficiently across the yeast ER, is an obligate co-translational SRP-dependent substrate across the mammalian ER (PMID 2834400). Also, the binding preference of the SRP54 M domain may differ between organisms. These caveats are important to note rather than making sweeping conclusions.

2) Rather than calling something a "true" SRP substrate, perhaps "obligate" is better.

3) The correlation with glycosylation and SP type involve numbers that are are quite small, especially for SRP-facultative. Plus, this correlation may have nothing to do with going to different translocons. For example, one alternative explanation is that the rate of signal cleavage is influenced by hydrophobicity or length. Given that signal cleavage timing influences glycosylation, this might be the reason for the correlation. Thus, it is a substantial over-interpretation to claim that this correlation establishes that alternative targeting systems guide decision-making.

4) The observation that Ncyto membrane proteins more frequently exhibit additional SRP binding periods 25-50 codons after the 1st binding event could simply reflect the possibility that Ncyto multipass proteins more frequently have a second TMD close to the first. I believe this is the case, but the authors should check this.

5) The idea that TMD insertion mediated pulling force somehow impacts the ribosome-Sec61 interaction is completely speculative and it is hard to imagine a plausible mechanism by which this could occur given that any force on the nascent chain would be transmitted to the peptidyl-tRNA and not anywhere near the ribosome-Sec61 interaction site.

6) The data in Fig. 4H would benefit from indicating how many substrates are in each category.

7) If the authors' data are analysed using the method of Chartron et al., does one now see "pre-recruitment"? In other words, is the difference in conclusion simply a matter of analysis, or is the difference also due to methodological differences? If the difference is mainly down to analysis method, then it is worth explaining to the reader what the key differences are, and why their method is preferred.

(Remarks on code availability)

Reviewer #3

(Remarks to the Author)

The study from and Kotan colleagues revisits the association of the ER targeting factor SRP with nascent chains in the yeast Saccharomyces cerevisiae using the well-established selective ribosome profiling technique, in combination with membrane fractionation and SRP-depletion. The experiments confirm previous findings using complementary and related methods that the majority of yeast SRP substrates are membrane proteins and only a small subset of signal sequences engage with SRP. As has also been previously reported, translocation of signal sequence-containing also occurs co-translationally indicating this is not a unique property of the SRP-targeting pathway, but applies also to Sec71 substrates too.
The study uses an almost identical approach to that previously performed by the Frydman group (Charton et al, 2016), which indicated pre-loading of SRP to mRNA prior to the onset of translation and/or synthesis of the targeting signal. The precise mechanism for this mode of binding has so far proved elusive. Here, strong association of SRP was only observed after emergence of the signal sequence/anchor consistent with the canonical mode of signal peptide recognition. Reanalysis of the Frydman data suggests the pre-associated SRP sites were of low affinity/low confidence casting doubt on their significance. Finally, and perhaps most interestingly, the authors address largely for the first time the question as to whether multiple cycles of SRP binding and release are required for polytopic membrane protein biogenesis, and find that with these substrates ribosomes can dissociate from the translocon and re-engage SRP for targeting of later TM domains, particularly after longer cytosolic loop domains.
Overall, the experiments are convincing and provide important confirmation and novel insight into protein targeting and biogenesis at the ER and will be of great interest to the field. The authors are world-leaders in the use of selective-ribosome profiling.
My only substantive criticism is the use of the SRP54 deletion strain to assess targeting in the absence of functional SRP. This strain grows very slowly following a complex adaptation process and, of particular relevance here, has reduced levels of ribosomes and reduced translation rates (Mutka et al, 2001). Analogous studies have used the inducible sec65-1 temperature sensitive mutant or inducible-degron systems to rapidly deplete SRP and thereby circumvent this adaptation. It would be important to address whether any of the effects observed here are indirect consequences of the adaptation and associated slowed translation rather than directly due to loss of SRP.

Specific points:

The number of signal peptides analysed seems relatively low eg Costa et al (20) report ca. 200 soluble yeast SP protein and a further ca. 130 with SP and TMD. I assume this is due to those not making the read threshold. It would be prudent to state what fraction of the different classes are being sampled here. Perhaps more importantly, is there any bias in the substrates detected? In particular, those which are short in length, which might impact their targeting pathway such as short secretory proteins.

A technicality, but Δsrp54 is not a complete deletion of SRP as Δsrp might infer, all the other components are still present, just the functionally essential SRP54 is missing. This is unlike other SRP gene deletions where the particle is more fundamentally destabilised, it would be helpful to clarify this. GFP tagging of SRP54, doesn't appear to fully complement with either N or C-trm fusion, are the differences in doubling times between WT, N and C-tagged and deletion statistically significant? Was there a reason for not tagging Srp72 which does fully complement (Mason et al, 2000) and results would also have been directly comparable with Chartron et al (2016)?

As mentioned above, assessing SRP-dependence might be better addressed in a sec65-1 strain, or auxin-degron system allowing rapid inducible inhibition of the SRP pathway without the adaptation that occurs in the null. To circumvent repeating all the experiments with rapid depletion, can one or other approaches be used to confirm SRP-dependence (eg in direct translocation assays) for some of the novel substrates identified by profiling in the null strain?

The window size of 9 in the Arst et al study was only chosen over 11 or 19 based on the then available structure of bacterial Ffh. Earlier studies such as Ng et al (1996) had used the larger Kyte-Doolittle window size of 11, is this any better match for predicting SRP-dependency? It would also be helpful to assess how the FILVW-PGS scores matches to SRP-dependency determined in the Costa at al study upon rapid depletion of SRP.

The legend of figure 2 appears truncated.

As already mentioned above, the Δsrp54 strain has lower rates of translation due to reduced levels of ribosomes, could this not contribute to the reduced ribosome collisions observed? Could the impact of reduced translation on frequency of collisions be in part addressed by looking at the profile data for known SRP-independent ribosome collision events in the dataset?

Ribosome profiling of the ribosome collision marker protein Hel1 (Matsuo and Inada, 2021) also indicated enrichment on secretome mRNAs before onset of SRP recognition, consistent with the findings here. However, it is interesting in this study loss of ribosome binding increased Hel1 association. How would these observations be rationalised eg by different pools of stalled ribosomes? The authors might wish to comment on these observations.

In figure S3 can the %age of targeting that is co-translational be shown in WT (SRP54) for comparison, or is this only those substrates that are co-translational in WT (i.e. WT is 100%), if the latter, can that be made clearer?

Figures S3C and D are lacking the schematic showing position of TMD/SP

In figure S3M can traces be shown for previously identified Snd TM substrates (eg Ynl181w) in the WT v Δsnd1 strain as was shown for Gas1.

It was perhaps slightly surprising that luminal loops also led to ribosome dissociation from the translocon (Fig. 4), is this still dependent upon the presence of the preceding TM? ie is this effect seen with soluble secretory proteins once the signal sequence is cleaved or where Nexo membrane proteins are initially targeted by a Sec71-dependent signal sequence? The SRP binding profile for Erj5 (Fig. 1H) suggests that is possibly the case for the latter.

There is a correlation between Ssb binding and ribosome dissociation for the examplars shown in the figure 4, can this also by shown in a meta-plot with Ssb binding against loop length.

In figure 5, there is a mix of mammalian and yeast terms for chaperones eg BiP and Ssb. Would be better to be consistent eg using Kar2 instead of BiP.

(Remarks on code availability)

Version 1:

Reviewer comments:

Reviewer #1

(Remarks to the Author)
The authors have addressed my concerns, and I have no further comments.

(Remarks on code availability)

Reviewer #2

(Remarks to the Author)
The authors have provided substantial additional work, analysis, and explanations in response to the referee comments, and this effort is greatly appreciated. The proteome-wide analysis is valuable for the field and will stimulate new directions of research. I am supportive of publication and do not need to review this study again. I would suggest the following minor text revisions before publication.

1) The further profiling analysis without detergent and using a tagged strain that has a normal growth phenotype is helpful and reassuring. Notably however, the difference in the SRP binding score between the two conditions for TMD containing proteins is pretty striking (mean around 10 for the sample with detergent and using the GFP-SRP54 strain, but around 40 for the sample without detergent using the GFP-Sec65 strain). This seems to be mostly down to detergent. The authors should comment about this in the paper because the difference is non-trivial (i.e., similar to the 4-fold difference seen between SP and TMD).

2) In discussing SRP-mediated arrest, it is probably worth noting that the strong arrest originally described (PMID 7309797) was an artefact due to the mixing of plant translation extracts with mammalian SRP. In homologous systems, no such arrest was seen (see PMID 2415357, 3027096, 3087745). Even in the mixed system, arrest was not seen for other substrates (PMID 3027096). Only when excess SRP is added is a modest slowdown seen (PMID 2556403). The structural explanation for the lack of arrest is that the Alu domain can simply move out of the way when elongation factors bind, allowing simultaneous binding of SRP and elongation factors (see Fig. 7 of PMID 26158507).

3) The authors cite PMID 8626442 as prior evidence that SRP mediates insertion of an internal TMD when the loop preceding it is long. I would suggest that this be removed because the primary purpose of that paper is to investigate features that promote re-initiation, with the role of SRP being a minor part. But more importantly, inspection of the data in that paper does not support the conclusion about SRP. The key data is in Fig. 4B of that study. When the loop is extended from 58 to 100, there is a very small increase in re-initiation, but the error bars are huge and this cannot be considered robust or statistically significant. More notably however, when the loop is further extended to 200, the effect is completely lost. So I would just omit lines 465-467.

(Remarks on code availability)

Reviewer #3

(Remarks to the Author)
The reviewers have now satisfactorily addressed the concerns I had raised adding addition data and correcting the minor errors.

(Remarks on code availability)

Open Access This Peer Review File is licensed under a Creative Commons Attribution 4.0 International License, which permits use, sharing, adaptation, distribution and reproduction in any medium or format, as long as you give appropriate credit to the original author(s) and the source, provide a link to the Creative Commons license, and indicate if changes were made.
In cases where reviewers are anonymous, credit should be given to 'Anonymous Referee' and the source.
The images or other third party material in this Peer Review File are included in the article's Creative Commons license, unless indicated otherwise in a credit line to the material. If material is not included in the article's Creative Commons license and your intended use is not permitted by statutory regulation or exceeds the permitted use, you will need to obtain permission directly from the copyright holder.
To view a copy of this license, visit https://creativecommons.org/licenses/by/4.0/

Reviewer #1 (Remarks to the Author):

Kotan et al. employed selective ribosome profiling (SeRP) to examine SRP interactions with nascent chains in yeast. Through careful execution of their experiments, the authors clarify some contentious interpretations from earlier studies and provide new insights into SRP-mediated protein biogenesis. A surprising finding is that most signal peptide-containing secretory proteins are targeted to the ER via an SRP-independent but co-translational pathway, whereas transmembrane proteins strictly rely on SRP-mediated co-translational targeting. Another key observation is that SRP appears to participate in repeated targeting of transmembrane domains (TMDs) of membrane proteins at the ER membrane. A potential concern is that the study relies primarily on a single technique without biochemical validation for some key findings. Nevertheless, the dataset is rich and provides valuable new insights into SRP's role in protein targeting in yeast.

Major Comments:

1) The authors appear to assume that yeast SRP and mammalian SRP behave similarly. Previous studies show that mammalian SRP can target a broad range of signal peptide-containing substrates. By contrast, the Sec61 translocon in mammals is more discriminatory, preferentially recognizing strongly hydrophobic signal peptides and often requiring additional translocon-associated factors such as TRAP and Sec63. It is possible that yeast SRP preferentially recognizes highly hydrophobic signals, such as TMDs, which may explain why most secretory proteins in yeast are co-translationally targeted in an SRP-independent but Sec71-dependent manner. Since mammals lack Sec71 and Sec72, SRP may have evolved to capture both weak and strong hydrophobic targeting signals. The authors should discuss these evolutionary and mechanistic considerations.

We agree with the reviewer that the extent to which yeast and mammalian SRP share substrate preferences is not yet fully understood, and we have revised the manuscript to more clearly distinguish between observations made in *S. cerevisiae* and what is currently known in mammalian systems.

At present, there is no high-throughput or comprehensive *in vivo* analysis of the mammalian SRP substrate pool. While mammalian SRP is capable of recognizing and targeting signal peptide-containing substrates in *in vitro* translation systems, these experimental setups, while very valuable to gain mechanistic insights, cannot capture the competitive environment present in cells, where substrates must compete for SRP engagement. Thus, the breadth of mammalian SRP substrate specificity *in vivo* remains unresolved.

In the revised manuscript, we interpret the findings in yeast and mammalian cells to point to a conserved alternative pathway in both organisms that can insert proteins into the ER both co and posttranslationally with the help of a Sec62-Sec63 complex. The evolutionary and mechanistic considerations are discussed as follows:

Previous work demonstrated that in yeast, SRP-independent proteins can be co- or posttranslationally translocated via Sec61 translocons associated with the SEC complex (Sec62, Sec63, Sec71, and Sec72)[37–39], with the help of ER luminal Hsp70 Kar2 to ratchet the nascent chains into the ER[40]. Our findings align with earlier work on secretory proteins that rely on Sec71 for ER targeting[39], revealing overlap with our SRP-independent classification (Fig. 2G).
In mammals, Sec62 and Sec63 are conserved and form a complex with Sec61 and luminal Hsp70[41], whereas homologs of Sec71 and Sec72 are absent. Similar to yeast, human Sec62 and Sec63 can function downstream of both co- and posttranslational targeting pathways, and are particularly important for efficient gating of weak signal peptides[42–45]. These observations suggest conservation of the core mechanistic principles of the complex across species, especially regarding substrates with less hydrophobic targeting signals. However, it remains unclear whether mammalian Sec62-Sec63 acts downstream of an SRP-independent targeting pathway or assists signal peptide gating following SRP-mediated targeting. Analysis of FILGW-PGS scores, which predict SRP binding in yeast, reveals a clear separation between signal peptides and TMDs in humans as well (Fig. S5H, and Fig. R1 below). This suggests that cytosolic recognition of these targeting signals in human cells may also involve alternative pathways.

We further note that SRP54 contains a conserved helix in both mammals and yeast that has been proposed to define a hydrophobic threshold for substrate engagement, with substrate displacement from the binding pocket only by sufficiently hydrophobic sequences (Voorhees et al., 2015). The human SRP54 helix contains more hydrophobic residues in comparison to the yeast homolog, which agrees with the overall shift to higher hydrophobicity values of targeting signals in human cells.

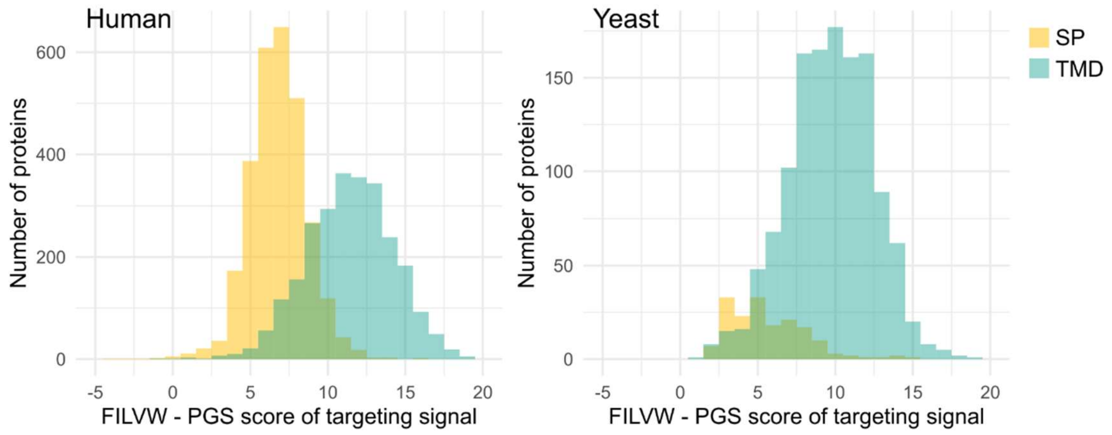

Figure R 1. Comparison of human and S. cerevisiae targeting signals. The sum of F, I, L, V and W residues minus that of central P, G or S residues in transmembrane domains (TMDs, turquoise) or hydrophobic regions of signal peptides (SP, yellow) were calculated. The distribution of the FILVW-PGS scores are compared side by side for the two organisms.

2) It is possible that SRP interactions with signal peptides of secretory proteins are weaker and thus not preserved during SRP-selective enrichment. Incorporating chemical or photo-crosslinking approaches could help address this possibility.

To address the reviewer's concern, we performed additional SeRP experiments with or without immediate BS^3 crosslinking upon lysate thawing. The results are included in the revised version of the manuscript (Fig. S1E,H,J). Immediate BS^3 crosslinking revealed essentially the same SRP

substrate spectrum, demonstrating that SRP avoids binding the majority of SP-containing proteins.

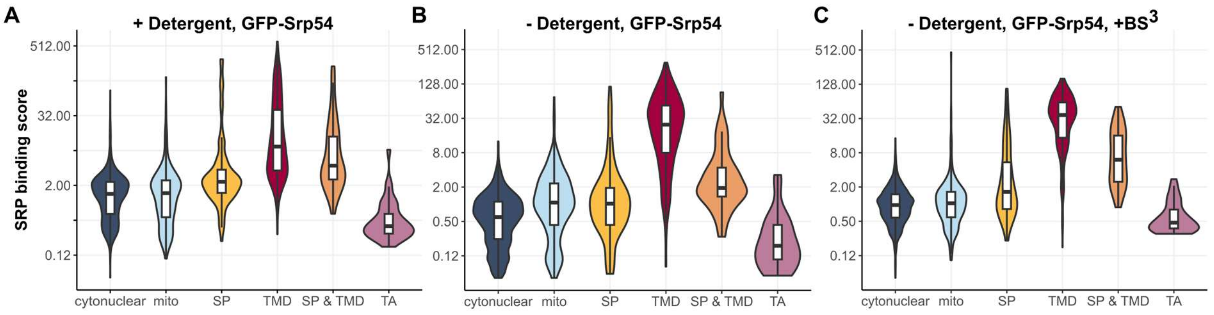

Figure R 2. Comparison of SRP binding scores derived from SeRP experiments performed with or without chemical crosslinking. SRP binding scores, defined as the maximum SRP enrichment (IP/total translatome) measured for each transcript, are compared across different substrate classes. Shown are results from standard SRP-SeRP experiments performed in the presence of detergent and without crosslinking, as well as from two additional SeRP experiments performed in the absence of detergent, either with or without BS^3 *crosslinking.*

3) While the ribosome profiling data are powerful, the manuscript could be strengthened by biochemical validation, particularly regarding the intriguing finding that SRP binds TMDs of membrane proteins even after ER targeting. The reviewer understands that biochemical validation may be technically challenging, but including such data or discussing potential approaches would improve the manuscript.

We agree with the reviewer that biochemical validation of SRP rebinding together with mutational analyses to dissect the features that promote SRP recruitment would be highly valuable. However, while we appreciate the value of complementary *in vitro* assays, establishing experimental setups that recapitulate our *in vivo* analyses is highly time consuming and must be pursued in a future, dedicated study. Importantly, we note that prior work using a wheat germ *in vitro* translation system provides biochemical support for our conclusions. In particular, Kuroiwa et al. (1996) examined insertion of the second TMD of a type I model membrane protein and found that insertion efficiency became dependent on SRP concentration when the luminal loop between the two TMDs was extended from 58 to 100 amino acids, consistent with a role for SRP in assisting TMD 2 insertion after initial ER targeting. In the revised manuscript, we have further highlighted these findings in connection to ours (Discussion section, page 21).

Minor Comments:

Line 258: The authors suggest that SRP skipping the first TMD in some transmembrane proteins may be due to TMD looping inside the exit tunnel. While this is plausible, there is no direct biochemical or structural evidence presented. It should be framed as one possible explanation rather than a definitive mechanism.

Although we do not provide new structural or biochemical evidence in this study, our reasoning is supported by prior biochemical observations from the Rodnina lab (Mercier et al., 2020), which are in strong agreement with SRP-specific ribosome profiling experiments in *E. coli* (Schibich et al., 2016). Nonetheless, we agree with the reviewer that this model is still preliminary, especially

with respect to yeast, and we have revised the text to clarify that TMD looping represents a plausible, but not exclusive, explanation.

Reviewer #2 (Remarks to the Author):

This study uses ribosome profiling of SRP-affinity-purified polysomes and polysomes generated by fractionation to investigate the role of SRP during secretory and membrane protein synthesis in yeast. The authors make a number of observations that lead them to several conclusions, some of which match current models and others that don't. Their main conclusions are that SRP preferentially binds to signal peptides (SPs) and transmembrane domains (TMDs) of high hydrophobicity, that it is recruited only once the SP or TMD has emerged from the ribosome, that the first TMD of a multipass membrane protein is sometimes 'skipped' by SRP, and that ribosomes detach during loop synthesis, with SRP being used to re-target these ribosomes when the next TMD emerges. They further conclude that SPs that don't engage SRP can nonetheless mediate co-translational targeting.

The datasets and analyses of such ribosome profiling studies are interesting and valuable to the field because they provide a global view of biochemical processes using a range of endogenous substrates and complement more focused biochemical and structural studies. However, the study as currently presented suffers from three general problems that would need to be addressed before publication. First, it uses only profiling methods with no attempt at corroboration of the more provocative or new conclusions with any orthogonal method or with more directed experiments. Second, the impaired growth of tagged SRP strains and the use of detergent in the SRP pulldowns may confound the results, leaving open quite plausible alternative interpretations of some of the key data. Third, there are a number of over-interpretations that go beyond what is justified by the data. Rectifying these problems is likely to require major revisions.

Major points:

1) The use of detergent in most of the experiments and in all of the SRP pulldowns has the potential to disrupt interactions between the SRP54 M domain and hydrophobic peptides. Even very small amounts of detergent (0.01%) have been shown to bind the hydrophobic groove of the M domain and mimic a signal peptide (see PMID 19119234), density in the M domain groove in the SRP54 crystal structure might have been detergent, which was required to obtain well-diffracting crystals (PMID 9695947), and detergent disrupts the analogous interaction between GET3 and its TMD substrate (PMID 17382883). Because the signal-SRP interaction is a major contributor to SRP-ribosome interactions, perturbing this may well influence what is recovered. In fact, the preference of SRP for substrates of high hydrophobicity can be readily re-interpreted as these being the least sensitive to detergent. If so, then many of the major conclusions in the paper have the potential to be incorrect or incomplete. The authors should most certainly be clear in the paper about these caveats so a reader can properly judge the study, and should ideally demonstrate in more directed experiments that yeast SRP has a strong preference for high-

hydrophobicity SPs and TMDs. This could be done by in vitro interaction or crosslinking analyses (very common in the field), or if they really wanted to probe the situation in cells, by site-specific photo-crosslinking via probes in the SP or TMD.

To address the reviewer's concern that detergents may perturb SRP-substrate interactions we have performed additional SRP-SeRP omitting detergents. As described below, these experiments reveal that the addition of detergent does not cause a specific destabilization of SRP-SP interactions and in the absence of detergent SRP still does not engage SPs.

First, we would like to note that our central conclusion that many SP-containing proteins are not SRP substrates *in vivo*, does not rely solely on our detection of preserved SRP-RNC interactions after cell lysis. In addition, we used soluble ribosome profiling that directly measures cotranslational targeting of ribosomes to the ER membrane. While both readouts are sequencing-based, they provide orthogonal information and converge on the same interpretation. In wild-type and Δ*srp54* strains, we identified proteins whose ER targeting is completely unaffected by SRP54 deletion; these show strong overlap with proteins classified as non-SRP substrates in our SeRP experiments, providing independent support for our conclusions.

As mentioned above, we have now performed additional SeRP experiments in the complete absence of detergent, with or without immediate crosslinking upon lysate thawing. The results are included in the revised version of the manuscript (Fig. S1G-J). Modifying these experimental conditions did not change the SRP substrate spectrum (See Fig R3. A-C, below), demonstrating that the lack of SRP association with many SP-containing proteins is not a consequence of detergent interference. Importantly, omitting detergent further pronounced the SRP preference for TMDs, as reflected by the enhanced binding scores of this substrate class. However, SRP-SeRP performed without detergents has significant technical limitations. One limitation is that even mild clarification of cell lysates removes most membrane-bound ribosomes and their associated footprints. Another is that the remaining membrane fragments can harbor multiple translocons and be bound by multiple ribosomes. This facilitates the copurification of ribosomes that are not directly engaged by SRP, and reduces both, the resolution and the specificity of SeRP. Therefore these datasets are not ideal to resolve SRP interactions that occur especially after initial ER-membrane targeting (i.e. retargeting type interactions), which lead us to use primarily our original SRP-SeRP dataset for the majority of the analyses.

We would like to further point out that although the previous probing of SRP-substrate interactions *in vitro* provided valuable mechanistic insight, such experiments generally do not simulate the complex environment present in cells, where nascent chains are constantly sampled by multiple factors and must compete for limited SRP. Furthermore, *in vitro* experiments employing purified components often use unphysiological factor concentrations and ratios, as well as saturating crosslinker concentrations. Together, these features very likely bias the system towards the formation of SRP crosslinks to include proteins that are not *bona fide* SRP substrates. Our primary goal in the present study was therefore to define the physiological substrate spectrum of the yeast SRP in its native cellular context, an issue that has remained largely

unresolved. The genome-wide analysis of factor-nascent chain substrates by SeRP is specifically designed to address this gap.

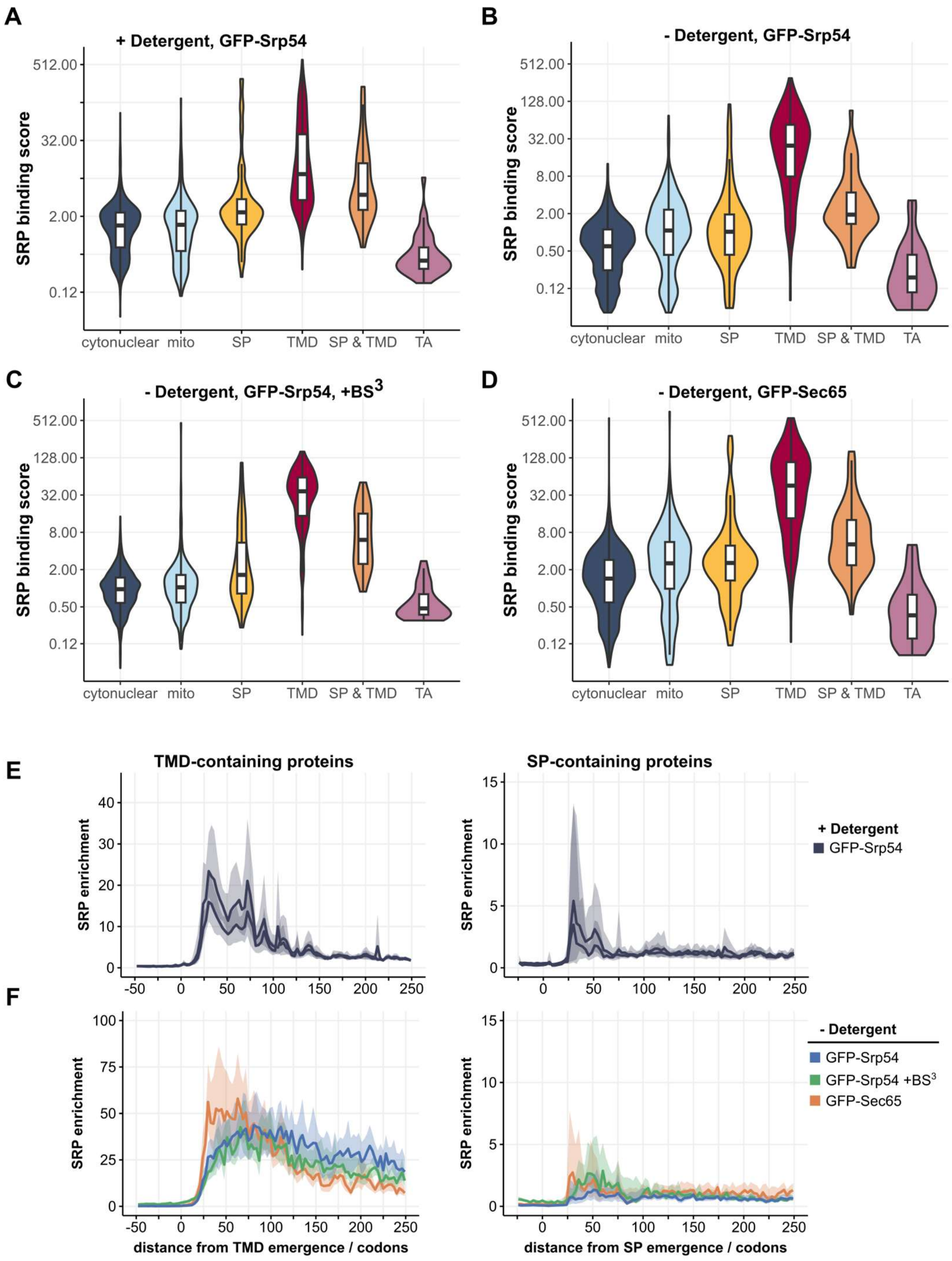

Figure R 3. A-D) Comparison of SRP binding scores derived from SeRP experiments performed with or without detergent and chemical crosslinking. SRP binding scores, defined as the maximum SRP enrichment (IP/total translatome) measured for each

transcript, are compared across different substrate classes. E-F) Metagene analysis of SRP interactions with TMD-containing or SP containing proteins with or without detergent and chemical crosslinking, based on SeRP using the indicated strains.

2) The authors seem unconcerned by the slower growth of their tagged SRP54 strains. However, the doubling time seems to increase by ~20 to 30 min, which is non-trivial and indicates a substantial fitness cost of tagging SRP54. Because the basis of this fitness cost is unknown, it is hard to determine whether it affects any of the results or not. For example, if steric issues partially obscure SRP54 access to substrates, it could wind up being stable only on the most hydrophobic ones. This too is an alternative explanation for its observed specificity in the profiling. Obviously, tagging a subunit that is not involved in substrate recognition, taking advantage of the available structural information, would have been preferable. The N-terminus of SRP19, for example, seems flexible and protrudes away from the important functional parts of SRP. Again, acknowledging these caveats at a minimum is needed, but ideally, one would verify the results using a tagging strategy that does not impair fitness and can be more confidently verified to be fully functional.

To address the reviewer's comment, we have performed another series of SeRP experiments analyzing nascent chain interactions of an N-terminally GFP-tagged Sec65, the yeast homolog of SRP19. Determination of doubling times revealed that tagging Sec65 does not affect cell growth (Fig. R4, below and S1F). We performed SeRP of the strain encoding tagged Sec65, in the absence of detergents. The results support our finding that SRP does not bind the vast majority of SPs (Fig R3D), as well as our findings that SRP is recruited only after the emergence of targeting signals (mainly TMDs). We believe that these results strengthen our findings and are now part of the revised manuscript (Fig. S1I-J).

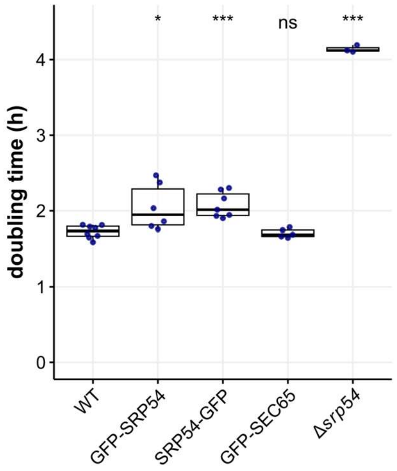

Figure R 4. Doubling times of yeast strains during logarithmic growth. Doubling times of each strain were compared to that of wild-type yeast, and statistical significance of the differences was assessed using a t-test (ns, not significant).

3) The conclusion that SRP skips some TMDs and instead binds downstream TMDs is intriguing, but not especially convincing given the above two caveats. It is supported modestly by the parallel timing of membrane association, but this is on individual mRNAs with somewhat noisy data and

doesn't always fit the authors' interpratation. For example, membrane targeting seems almost complete before the onset of SRP binding in the case of DGK1, so it doesn't seem like the major SRP binding event is responsible for targeting. Furthermore, the example of ERJ5 seems to indicate that something odd is happening here and that downstream binding might be some odd artefact. In ERJ5, the signal would mediate targeting and initiate translocation of the N-terminus. The fractionation supports targeting of the majority of nascent chains shortly after the signal emerges but before the TMD is synthesized. So the ribosome would be docked on Sec61 and in the process of translocating the N-terminal soluble domain. According to pretty well-established principles, the TMD would then emerge from the ribosome into Sec61, then partition laterally into the bilayer. So when exactly in this scheme is SRP purported to bind the TMD? Even if the idea is that the signal fails to be recognised and targeting does not happen until the TMD emerges, how would SRP binding to the TMD and targeting to the ER result in a protein of the correct topology? Given this odd result, one must wonder whether the 'skipping' of TMDs and binding downstream TMDs in other examples is a valid result. This can be resolved by directed experiments that analyse SRP binding to different length nascent chains in vitro for a substrate where the profiling suggests TMD1 is skipped and TMD2 is engaged.

First, we want to point out that the model of SRP skipping low hydrophobicity TMDs and binding an internal TMD to mediate ER-targeting has previously been explored in *E. coli*, supported by mutational analysis (Schibich et al., 2016). Our study extends this concept to the yeast SRP system, while not extensively focusing on it. We do not agree with the reviewers comments that our data is only supported by individual cases, or that the case of Dgk1 does not fit our interpretation as further detailed below. Rather, our model of SRP skipping TMDs is supported by meta-analysis as well as multiple examples of TMD-containing proteins in both yeast (this study) and *E. coli* (Schibich et al., 2016). In the revised manuscript, we also clarify our interpretation of the Erj5 data. We don't agree that skipping of the N-terminal TMD is inherently problematic for acquisition of correct topology as TMDs don't always insert into the membrane in the order of their synthesis, which we have further discussed below.

We agree that individual gene profiles can appear noisy, an inherent consequence of the high variability in expression levels *in vivo* and the high positional resolution of ribosome profiling. However, as noted by the reviewer, our model is robustly supported by two independent lines of evidence: (i) the measurement of SRP association by IP of SRP-RNC complexes and (ii) the measurement of RNC-membrane engagement by ribosome fractionation, in both cases followed by the identification of nascent chains by deep-sequencing of ribosome footprints. Contrasting previous studies, our interpretations do not rely on single examples but are also supported by metagene profiles. The data show a general delay of SRP binding onsets for less hydrophobic TMDs (Fig. S5D), consistent with selective engagement of downstream hydrophobic elements. To address the reviewer's comment, we here provide additional representative examples (Fig. R5A) to better illustrate the general behavior. These examples can be added to the manuscript if requested by the reviewer.

Regarding DGK1, SRP binding initiates before position ~150, shortly before the TMD2 is fully exposed (see Fig. R5B below for clarity). Importantly, at this stage membrane targeting is far from

being complete: the soluble/total ribosome ratio remains at 1, indicating that 100% of ribosomes are still cytosolic. Accordingly, the enrichment level during the first, rather weak SRP binding period is low and efficient targeting is detected only after a second, much more prominent SRP-binding phase. Contrary to the reviewer's suggestion that GFP tagging of SRP54 or the use of detergent might artificially generate this effect, SRP-selective ribosome profiling performed in the GFP-SEC65 yeast strain, in the absence of detergent, likewise reveals SRP interactions only after TMD2 emergence.

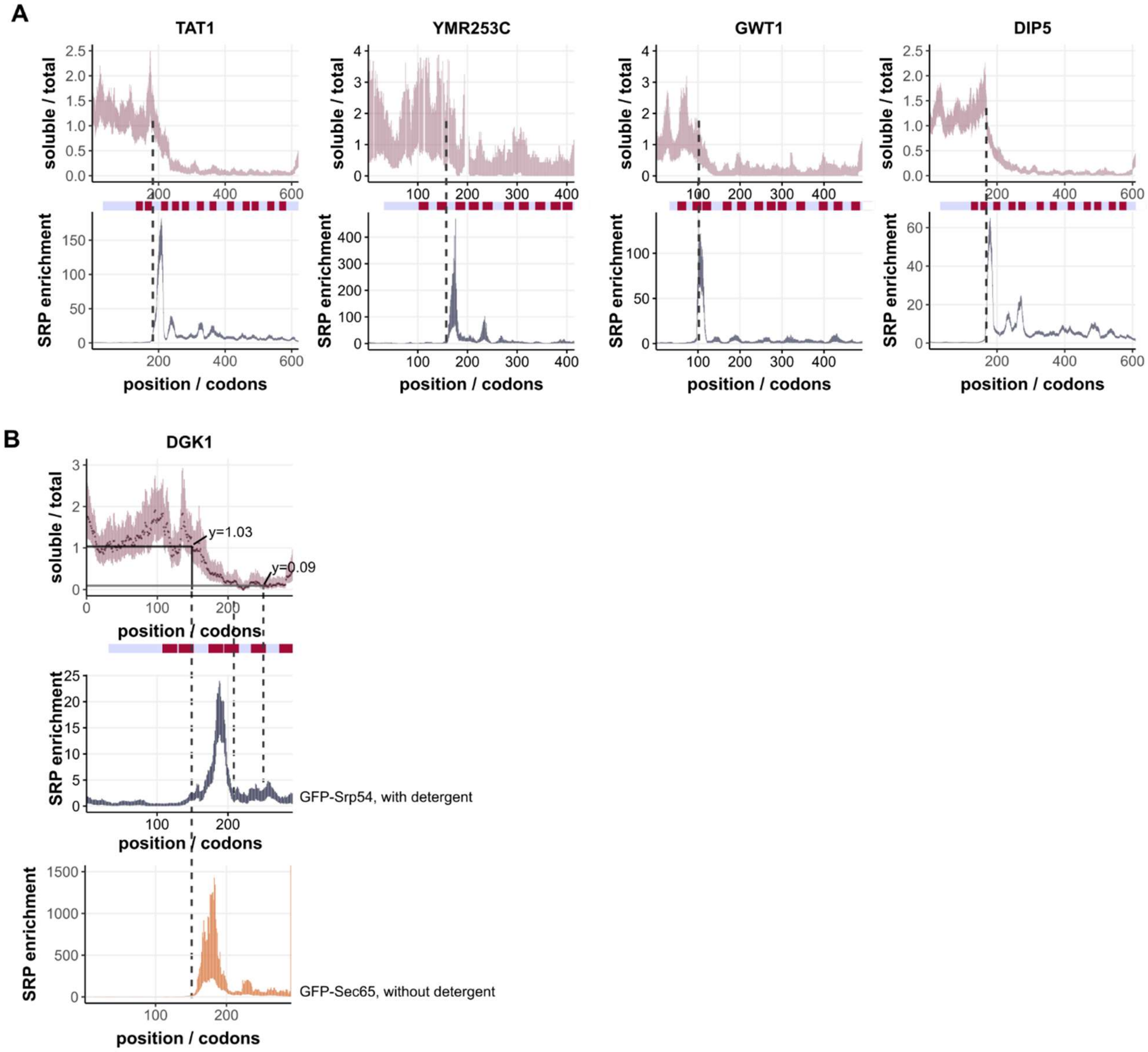

Figure R 5. Examples of SRP skipping of the most N-terminal TMD by SRP. A) Targeting profiles based on soluble-RP and SRP interactions based on SeRP are depicted. TMD positions, corrected for the exit tunnel are depicted in red. B) Targeting and SRP-binding profiles of DGK1 are compared. The values of soluble/total translatome ratios are depicted. Shadows indicate the 95% confidence interval. SRP binding profiles are derived from SRP-selective ribosome profiling experiments performed in strains encoding GFP-SRP54 (blue), or GFP-SEC65 (orange), performed with or without detergent, respectively.

Erj5 is certainly an interesting example, where fractionation data indicate that only a small subset of ribosomes synthesizing Erj5 is delivered to the membrane right after its SP emerges, likely via an SRP-independent pathway, consistent with the absence of detectable SRP binding to its signal peptide. At this translation period, the majority of Erj5 ribosomes remain soluble and is efficiently targeted after SRP binding to the emerging downstream TMD. Thus, the SRP-engaged ribosomes are not pre-docked on Sec61 but represent a distinct population that is targeted late, via SRP binding to the internal TMD.

While the reviewers question regarding how the correct topology is achieved is definitely an important one, we don't believe that argues against TMD skipping. After TMD insertion in an Nout topology, different mechanisms may assist the insertion of the SP and the translocation of the luminal segment, located in-between SP and TMD: The luminal domain may be pulled into the ER by folding or assisted by chaperones. Analogous to the posttranslational insertion of SP-containing proteins described in yeast, alternative systems may exist that help inserting SPs and translocating luminal segments. Supporting this model, non-sequential insertion of membrane segments (i. e. not in the order of their synthesis) has been observed in several studies before (reviewed in Shao and Hegde, 2015). We consider it plausible that skipping of the most N-terminal TMD plays an important role in topology determination, allowing the properties of the second TMD to dictate the overall topology of the protein.

While *in vitro* analyses of stalled ribonucleoprotein complexes (RNCs) can provide insights into SRP's binding capabilities, they have limitations when it comes to addressing our core question: which substrates are productively engaged by SRP under physiological conditions *in vivo*, where multiple factors compete for interaction. The artificial environment of *in vitro* assays, often characterized by non-physiological component concentrations, prolonged exposure times, and crosslinking stabilization, can reveal interactions that may have limited biological relevance or even be unproductive in cellular contexts. To overcome these limitations, our approach was specifically designed to discern authentic, physiological SRP substrates from those that can bind SRP only under permissive, non-physiological *in vitro* conditions.

4) The loop formation inside the ribosome being the basis of late SRP binding is not well supported for a number of reasons. First, the example of EmrE might simply reflect the fact that TMD1 is not as hydrophobic as TMD2. Second, many Ncyto signal anchors are preceded by long segments of polypeptide, so cannot sterically form a loop. Third, the one situation where a hydrophobic domain is necessarily at the extreme N-terminus and is certainly inserted into the membrane as a loop is a cleavable SP. Yet considerable biochemical data using crosslinking with nascent chains of defined lengths indicate that SRP binds immediately after the SP emerges. In the authors own profiling data, binding of SRP to SPs is consistent with binding as it emerges, not delayed due to loop formation. Thus, the loop idea seems very unlikely to be generally true and is highly speculative. If the authors want to propose this, they would need a more convincing argument.

We agree with the reviewer that the looping model is at a preliminary stage at the moment and very likely affects only a subset of nascent chains. We have now further emphasized this in the

manuscript. However, studying translatome-wide SRP interactions and finding delayed initial binding to certain proteins, we find it highly logical and necessary to follow up on evidence provided by the Rodnina lab, which had reported that nascent chain looping delays ER-targeting (Mercier et al., 2020). As our previously published *E. coli* SRP-interactome is in clear agreement with their biochemical findings, we believe it would be misleading to not discuss this aspect with respect to our yeast data.

More specifically, we would like to clarify first that our manuscript discusses EmrD, not EmrE. The reason we chose this example is that EmrD has been studied extensively by Rodnina and colleagues, using a combination of real-time FRET measurements and protease protection assays (Mercier et al., 2020). Their data provides strong experimental evidence that the nascent EmrD chain adopts a looped conformation within the ribosomal exit tunnel, prior to membrane insertion. Furthermore, the EmrD example cannot be explained by a weaker hydrophobicity of the first TMD (TMD1), as it is substantially more hydrophobic than TMD2, consistent with preferential membrane insertion of TMD1. Using ΔG predictions for membrane insertion (dgpred.cbr.su.se), TMD1 has a predicted ΔG of 0.367, whereas TMD2 has a ΔG of 3.916.

We agree that open questions remain regarding the precise topology of nascent chains in the looping model. In particular, it is not excluded that portions of the N-terminal cytosolic segment may also be transiently retained within the exit tunnel.

With respect to signal peptides it is more difficult to assess a delay in SRP binding to these signals as our profiling data reveal overall weak SRP association with SPs. That said, we do observe delayed SRP binding to specific SPs, such as the SP of DSE2, suggesting a fraction of these nascent chains may also form a loop within the exit tunnel (Fig. 2E, see second minor peak).

5) The authors conclude on the basis of the flat soluble:total ratio (Fig. 2A) that membrane proteins are targeted post-translationally in ΔSRP cells. This seems highly unlikely as there is really no good paradigm for post-translational membrane protein insertion at the ER under any circumstances, except for tail-anchored proteins. I am not sure how the soluble:total ratio is calculated or normalised to 1.0, but if the ratio is unchanged, there seem to be two explanations: either the mRNA is mostly in the cytosol and stays there, or is mostly at the membrane and stays there. Of the two, the latter makes more biological sense. Is there a way to have a more quantitative and non-normalised assessment of mRNA location in WT versus ΔSRP cells to clarify this issue? In the authors' model, membrane protein mRNA should be mostly membrane associated in WT cells, but cytosolic in ΔSRP cells. This can be easily tested.

We thank the reviewer for this thoughtful remark offering two alternative explanations for our data. However, we are convinced that our data clearly support our previous interpretation for the following reason:

Our method is in fact quantitative and we can rule out the option that mRNAs are membrane associated all the time, because this would result in a major depletion of these mRNAs in our soluble translatome sample, but not in the total translatome sample. The ratio plot of these two samples would result in flat lines, centered at a value way below 1 and close to 0.

The issue raised by the reviewer comes from a misunderstanding of our data analysis scheme which we now explain in more detail below and in the main text of the manuscript:

To derive the soluble/total ratio, first, normalized read counts on each codon position are calculated by dividing read counts by the total number of genome-aligned reads of the corresponding sequencing library (i.e. total or soluble translatome). To explain the data analysis better, we use the example of RTN1 (shown as Fig. R6A below and Fig. S3 in the manuscript). The distribution of these values on RTN1 are depicted in terms of Reads Per Million mapped reads (RPM) (Fig. R6A, top two panels).

For the total and soluble translatome samples, the ribosome distribution (revealed by the RPM value) along an open reading frame is expected to be roughly the same, unless membrane targeting occurs, leading to ribosome docking onto the translocon. Membrane targeting can be quantitatively assessed by calculating the ratio of reads in the soluble and total translatome samples at each codon positions. A soluble/total ratio which centers around 1 indicates that translating ribosomes are not connected to membranes. Importantly, the ratio of ~1 arises from the fact that RPM values in the soluble and total translatomes are close to equal at that position, it is not artificially set (or normalized) to equal 1. Upon membrane targeting, the ratio is reduced to a value close to 0. Thus, this ratio directly allows to quantify what fraction of translating ribosomes remains soluble at each codon position.

If ribosomes translating specific transcripts were associated with membranes right from the initiation of translation, we would expect a significant depletion of reads in the soluble translatome sample compared to the total translatome throughout translation (Fig. R6 below, C). This would result in soluble/total ratios substantially below 1. However, our data contradict this model. Instead, we observe that the profiles of cotranslationally targeted proteins initially exhibit enrichment levels around 1, which then decrease as membrane targeting occurs, consistent with engagement of the ribosome with the membrane. In the *SRP*Δ background, these profiles are flat at the value one, indicating absence of cotranslational targeting.

Two possibilities remain for proteins with flat profiles: either they are not targeted to the membrane or they undergo posttranslational targeting. Given that it is unlikely that cells could survive without inserting many of these proteins into the membrane, we find it likely that at least a fraction of them are posttranslationally targeted. However in our manuscript we have not made

such a statement, but stated that proteins reaching the ER membrane in *SRP*Δ cells, for which we observe no cotranslational targeting, must be inserted posttranslationally."

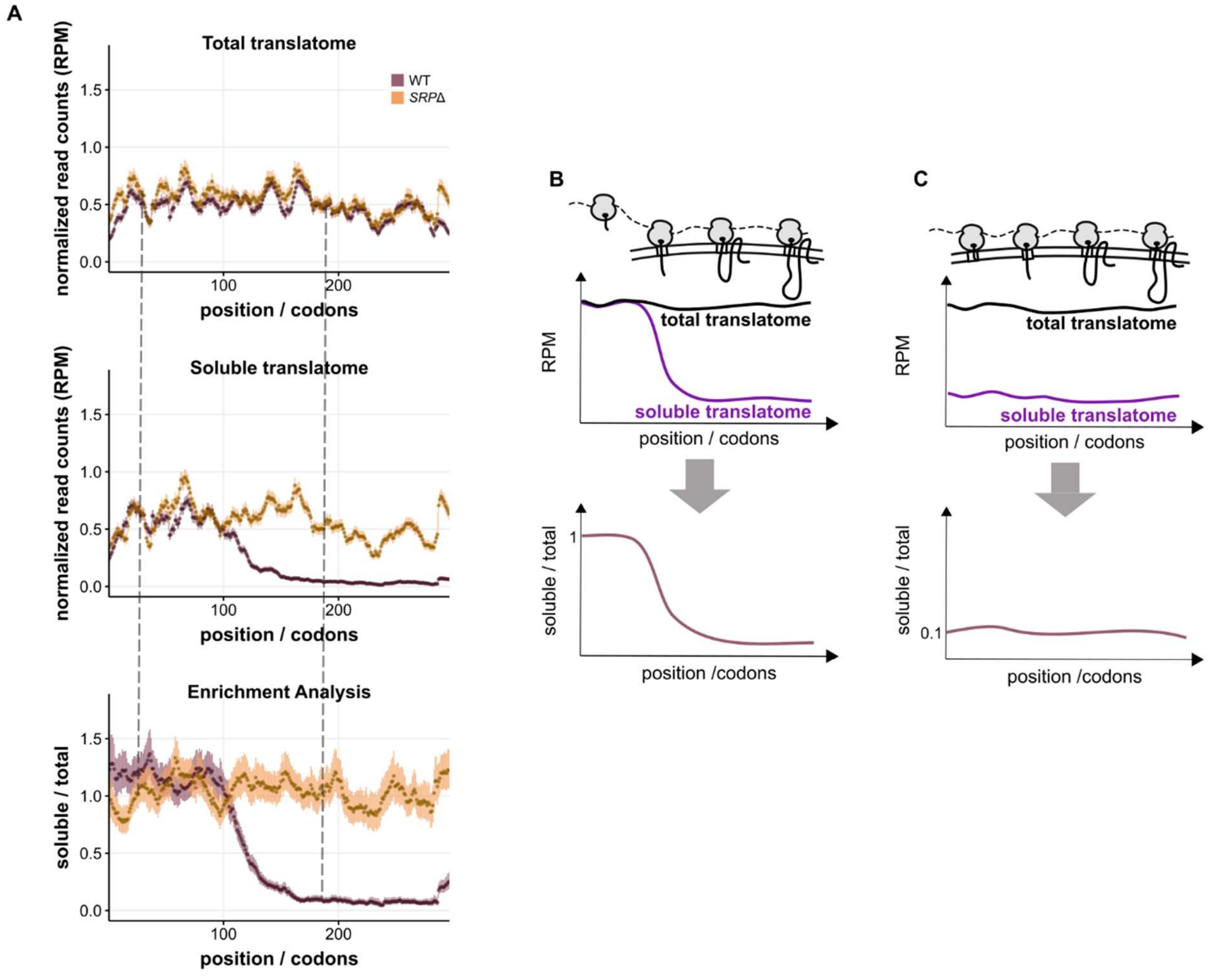

Figure R 6. Data analysis of soluble-RP data. A) Using RTN1 as an example, RPM values calculated at each position across this transcript in the total translatome (top panel) and the soluble translatome (middle panel) are depicted. The enrichment analysis (bottom panel) is performed by calculating the ratio of the RPM values in the soluble translatome over that in the total translatome for each codon position. B) The expected RPM distributions and soluble / total translatome profiles of a cotranslationally targeted protein, or C) of a protein translated by ribosomes attached to the ER-membrane from the start of translation. Ribosome illustrations have been adapted from Koubek J, Schmitt J, Galmozzi CV and Kramer G (2021) Mechanisms of Cotranslational Protein Maturation in Bacteria. Front. Mol. Biosci. 8:689755. doi: 10.3389/fmolb.2021.689755 under a CC BY license: https://creativecommons.org/licenses/by/4.0/.

6) The conclusion that "many multipass membrane proteins require repeated SRP action for efficient insertion of all TMDs" is very speculative given that there is no functional data that SRP is required beyond the initial targeting step, or that in the absence of a putative SRP re-targeting event, downstream TMDs are not inserted. Furthermore, while the profiling data for some substrates like PHO88 and PUN1 seem to support the model, others do not. For example, HXT2 shows substantial undocking, but a barely discernible SRP re-binding (at least 10-fold lower than the initial binding). The same is true for PDR5. For OLE1, SRP re-binding is robust, but there is barely any ribosome undocking. One therefore wonders whether cherry-picked examples are used to propose an intriguing model that may not be generally applicable.

We agree that this data alone does not suffice to claim that retargeting cannot occur without SRP. However, our hypothesis is supported by previous *in vitro* work, showing that SRP is required for proper TMD insertion when loops have been extended. In particular, Kuroiwa et al. (1996) examined insertion of the second TMD of a type I model membrane protein and found that insertion efficiency became dependent on SRP concentration when the luminal loop between the two TMDs was extended from 58 to 100 amino acids, consistent with a role for SRP in assisting TMD 2 insertion after initial ER targeting. We have further emphasized the connection of our findings to this previous study in the revised version of the manuscript.

Notably, the second SRP binding period on HXT2 exhibits a prominent enrichment of approximately 10-fold. Similar to many other membrane proteins, the enrichment value of the second peak is lower than that of the first peak. This decrease of enrichment is likely due to the more transient nature of the retargeting-type interactions, which occur when RNCs are in closer proximity to the membrane compared to initial ER targeting. Consistent with this, we observe that TMD1 targeting of Hxt2 is completed upon translation of about 60 codons, while retargeting of TMD2 is completed within a much shorter time window of only 20 codons. We propose that this transient nature of the retargeting is one reason why we capture fewer of these events.

The generality of our findings is underscored by the metagene profiles of TMD-containing proteins (Fig. 1D), which reveal SRP enrichment that persists beyond the emergence of the first TMD. To further illustrate this point, we provide below additional single-gene profiles for several representative genes.

In summary, we consider our evidence, combined with the already published data, compelling enough to justify our statements that SRP often reengages ribosomes that have docked off from the translocon, and that this is required for efficient retargeting of at the very least a subset of multipass membrane proteins.

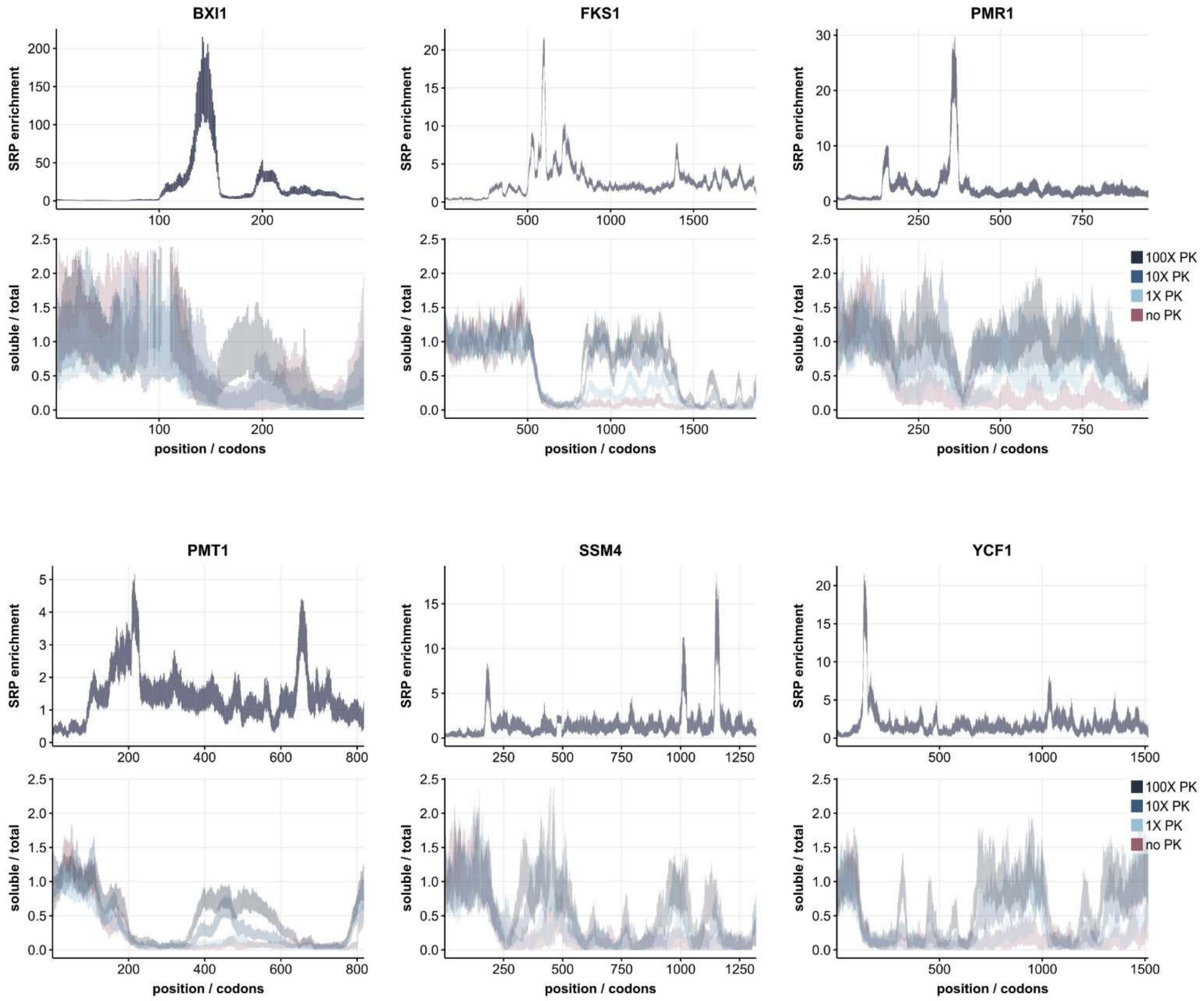

Figure R 7. Additional examples of SRP binding to undocked ribosomes.

7) The claim that cytosolic loops result in preferential ribosome undocking is not very strongly supported given that luminal loops also result in substantial undocking (Fig. 4E). This is a bit worrisome concerning the interpretation of the undocking data because translocation of luminal domains is pretty well established to occur through Sec61 via a docked ribosome. Furthermore, even if ribosomes did detach occasionally, the nascent chain would not easily slip back due to a combination of folding and chaperone binding on the luminal side. Finally, if the idea is that ribosomes detach during luminal domain translocation, then detachment should be seen during secretory protein translocation, or during Ncyto single-pass protein translocation. For these reasons, the conclusion that ribosomes undock during cytosolic loop production, while attractive, is not especially strongly supported by the available data.

We disagree for the following reasons:

First, our data show that cytosolic loop synthesis is associated with a substantially higher frequency of ribosome undocking as compared to luminal loop synthesis, consistent with our hypothesis that undocking is more important for synthesis and folding of soluble cytosolic domains. Quantitatively, we observe that >75% of RNCs are undocked during synthesis of long cytosolic loops, compared to ~50% during synthesis of luminal loops (Fig. 4E).

Second, we disagree with the reviewer´s statement that translocation of luminal domains is pretty well established to occur through Sec61 via a docked ribosome. To our knowledge, the docking state of the ribosome on the translocon during different translocation stages is not well explored, although it is typically assumed to occur on docked ribosomes.

Third, our data does not contradict the reviewer's suggestion that the nascent chain would not slip back out. Our proteinase sensitivity assay reports on ribosome undocking from the translocon that allows proteinase K to access the membrane-associated nascent chain, and not on the complete dissociation of the RNC from the membrane.

Finally, as the reviewer notes, folding and chaperone binding on the luminal side may retain ribosomes close to the translocon although ribosome-Sec61 contacts are lost. Because of these reasons, ribosome undocking during secretory protein translocation and Ncyto protein translocation is plausible, and is observed in our data (Secretory proteins: Figure S6L, Nycto single-pass proteins: Pho8, Spc3 below). We have further revised our discussion, mentioning the potential relevance of folding of luminal domains, in addition to binding binding of ER luminal factors previously discussed (Page 22).

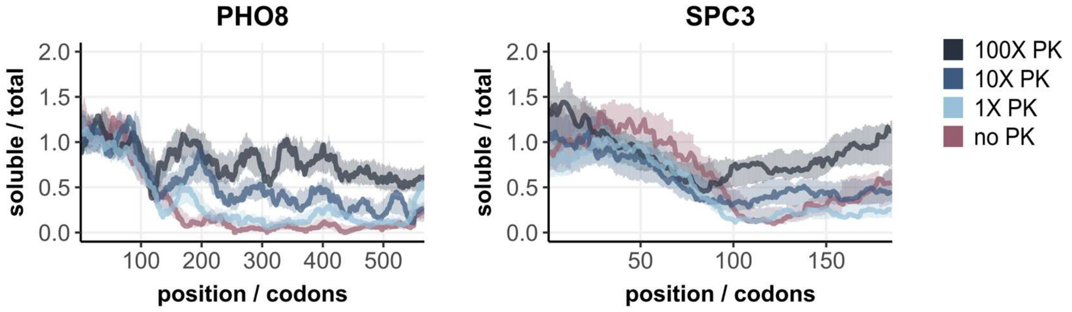

Figure R 8. Ribosome undocking during translocation of secretory proteins.

8) The Ssb interaction is an interesting alternative way to support the ribosome undocking idea, but is shown for two somewhat noisy examples. Is there any meta analysis of this to provide stronger support for the idea?

We disagree with the reviewer that these examples are noisy. Ssb engages the nascent chain in multiple cycles, resulting in multiple interaction periods observed prior to targeting and during the membrane-undocked stages.

To directly address the reviewer's question, we used an unbiased Ssb binding-detection analysis, and compared the number of Ssb binding periods across proteins with long cytosolic loops, long luminal loops, and short loops. To specifically exclude interactions that may occur prior to membrane targeting, we only included Ssb binding periods that occur after the onset of co-

translational targeting. This analysis revealed a clear Ssb preference for membrane targeted proteins harboring long cytosolic loops. These results are now presented below and in Figure 4G.

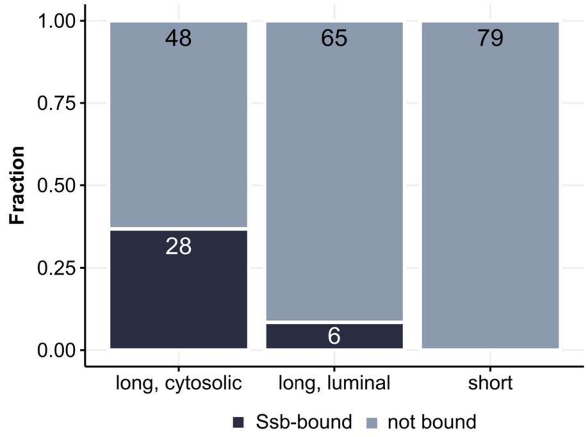

Figure R 9. Ssb binding preferences after cotranslational ER-targeting. Fraction of membrane proteins, categorized by the presence of long cytosolic loops, long luminal loops, or only short loops, that engage Ssb following cotranslational targeting to the ER.

9) SRP RNA is known to be sensitive to nuclease digestion at the hinge between the S domain and Alu domain. Do the authors know whether their RNase digestion conditions split SRP into these two domains? This is important because it may impact the stability of SRP binding to the ribosome, especially in the presence of detergent.

To address this valid concern, we have tested whether RNase treatment negatively affects SRP-RNC interactions. Specifically, we performed co-sedimentation experiments using sucrose cushion centrifugation to assess the effect of RNase treatment on co-sedimentation of SRP with ribosomes. The data suggest that RNAse treatment does not significantly reduce the SRP co-sedimentation (Figure R11, below, and Fig. S1D).

In addition, we have now performed SRP-SeRP with chemical crosslinking prior to RNase treatment, and in the absence of detergents. Although the absence of detergents reduces the resolution of our profiling, the results clearly reproduce the SRP substrate spectrum in terms of when SRP first binds its substrate nascent chains and which protein classes it engages. The data are included in the revised manuscript (Fig. S1H,J).

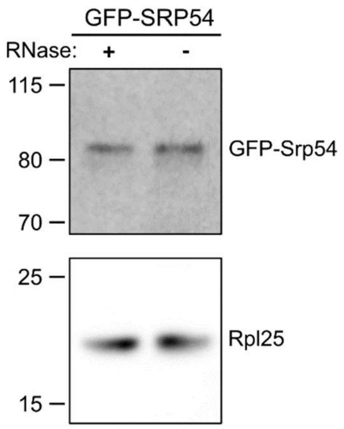

Figure R 10. Testing effect of RNase I treatment on the association of Srp54 with ribosomes. Yeast lysates were incubated with or without RNaseI (10U/A260, 30 min, 4 °C). The ribosome fraction was purified using a sucrose cushion and associated GFP-Srp54 was detected using a GFP antibody. Rpl25 was detected as a loading control.

Minor points:

1) It is important to be clear throughout that the data apply to yeast and that the degree to which it can be extrapolated to other systems (e.g., mammalian) is sometimes unclear. The SRP system is of course exceptionally well conserved, but there are nonetheless major differences in targeting. The main one is that as far as we know, the mammalian system is capable of post-translational translocation of only a small set of special substrates (namely very small secretory proteins - see PMID 22648169 & 22196732). Even substrates such as yeast alpha-factor, which can be post-translationally translocated efficiently across the yeast ER, is an obligate co-translational SRP-dependent substrate across the mammalian ER (PMID 2834400). Also, the binding preference of the SRP54 M domain may differ between organisms. These caveats are important to note rather than making sweeping conclusions.

We agree with the reviewer that it is important to clearly state that our conclusions are based on data obtained in *S. cerevisiae* and to avoid overextending these findings to mammalian systems. We have revised the manuscript accordingly (page 11).

The reviewer also highlights a potentially important distinction: In yeast translation extracts, longer SRP-independent substrates can be posttranslationally translocated, whereas mammalian microsomes appear to lack this capacity. The differences may stem from different chaperone composition or different substrates (e.g., α-factor that are not native to mammalian systems). Importantly, our data demonstrate that SRP-independent (and SRP-dependent) membrane targeting in yeast occurs predominantly cotranslationally, similar to mammalian systems.

While SRP-independent targeting in yeast cells relies on the SEC translocon (Sec62, Sec63, Sec71, Sec72), in human cells Sec62/Sec63 are conserved and, Sec71 and Sec72 are absent. A Sec62-dependent pathway mediates posttranslational targeting of small secretory proteins (Lakkaraju et al., 2012). At the same time, additional evidence suggests that it can also function cotranslationally, as Sec62 associates with ribosomes (Müller et al., 2010) and Sec63 has been

implicated in supporting cotranslational insertion of selected substrates (Lang et al., 2012; Conti et al., 2015). Together, these findings suggest that SRP-independent targeting in mammalian cells can operate in both co- and posttranslational modes and converges at the Sec62/Sec63 complex. We have revised the manuscript to include the mentioned evidences regarding evolutionary conservation of membrane targeting pathways.

2) Rather than calling something a "true" SRP substrate, perhaps "obligate" is better.

Our intention in using the term "true" was to emphasize that these proteins are not only capable of being targeted in the absence of SRP, but also that they are not detectably bound by SRP in wild type yeast cells. We believe that referring to them simply as "non-obligate" SRP substrates does not fully capture this latter aspect. Nevertheless, to avoid potential ambiguity, we have removed the adjective "true" from the text.

3) The correlation with glycosylation and SP type involve numbers that are are quite small, especially for SRP-facultative. Plus, this correlation may have nothing to do with going to different translocons. For example, one alternative explanation is that the rate of signal cleavage is influenced by hydrophobicity or length. Given that signal cleavage timing influences glycosylation, this might be the reason for the correlation. Thus, it is a substantial over-interpretation to claim that this correlation establishes that alternative targeting systems guide decision-making.

We agree with the reviewer that the absolute numbers underlying this analysis are small, particularly for the SRP-facultative group. Moreover, alternative explanations are possible.

That said, the difference between SRP-dependent and SRP-independent classes is statistically significant, and the SRP-facultative proteins, despite their small number, follow the same directional trend. Importantly, this correlation may address a central question raised by our data: Why is a subset of ER-destined proteins excluded from the SRP substrate pool? One attractive possibility is that early triaging in the cytosol ensures high efficiency at minimal cost and reduces the need for downstream sorting and quality control mechanisms.

This interpretation is further supported by the mutually exclusive interaction modes of the factors implicated in the biogenesis of the different classes. However, we understand the authors concerns and have therefore further emphasized in the revised text in the results and discussion sections that this is a preliminary model**,** explicitly acknowledging that it is currently supported primarily by correlative evidence.

4) The observation that Ncyto membrane proteins more frequently exhibit additional SRP binding periods 25-50 codons after the 1st binding event could simply reflect the possibility that Ncyto multipass proteins more frequently have a second TMD close to the first. I believe this is the case, but the authors should check this.

We thank the reviewer for raising this interesting point. Performing the suggested analysis, we indeed found that Ncyto proteins more frequently have a second TMD close to the first (i. e. within 30 amino acids of the 1st TMD). Performing a metagene analysis on Ncyto and Nexo proteins that have no TMD close by, we saw that the second SRP interaction period observed in our metagene analysis (Fig. S5E) is still detectable but less pronounced. However, this does not refute our model, as the presence of the second TMD might be crucial for looping, stabilizing the loop formation by interacting with the first TMD within the exit tunnel. We have now discussed this in our manuscript.

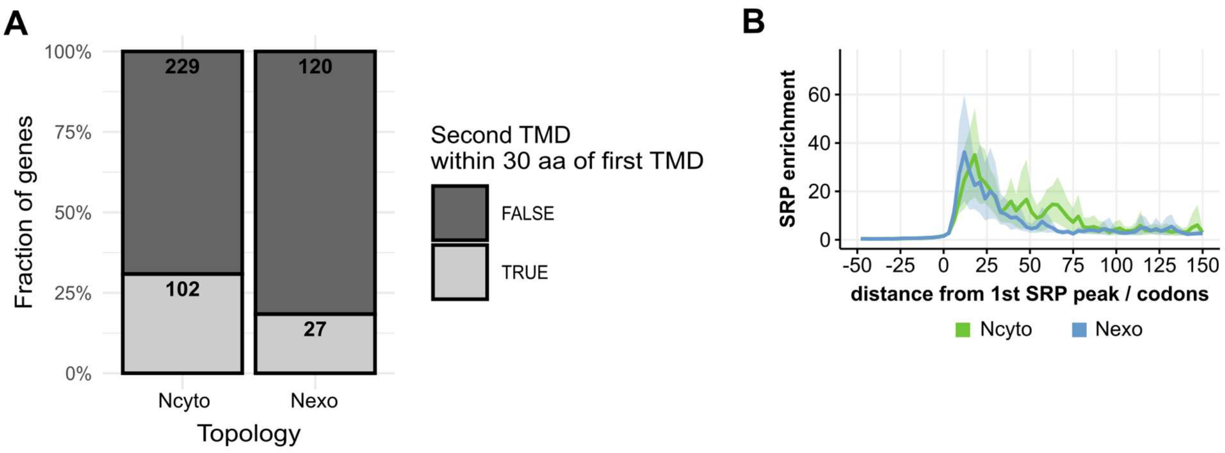

Figure R 11. Analysis of the effect of a second TMD on the proposed delay of SRP binding due to loop formation in the tunnel.

5) The idea that TMD insertion mediated pulling force somehow impacts the ribosome-Sec61 interaction is completely speculative and it is hard to imagine a plausible mechanism by which this could occur given that any force on the nascent chain would be transmitted to the peptidyl-tRNA and not anywhere near the ribosome-Sec61 interaction site.

We agree and have modified the revised manuscript to more clearly indicate that this interpretation is speculative. We nevertheless think that the clear correlation between high dissociation probability and high hydrophobicity of the inserted TMD is striking and warrants presentation. As mentioned above, the model is framed in the revised paper as one possible explanation among others that we discuss.

6) The data in Fig. 4H would benefit from indicating how many substrates are in each category.

We have now indicated the number of proteins in each category.

7) If the authors' data are analysed using the method of Chartron et al., does one now see "pre-recruitment"? In other words, is the difference in conclusion simply a matter of analysis, or is the difference also due to methodological differences? If the difference is mainly down to analysis

method, then it is worth explaining to the reader what the key differences are, and why their method is preferred.

There are two major differences between our method and the method of Chartron et al. that we expect for the Chartron study to have reduced the abundance of SRP-RNC complexes in the analyzed cells and lysate, prevented the detection of existing ones and resulted in the interpretation of background binding as "pre-recruitment" of SRP.

1) The pre-recruitment examples presented in the Chartron study are based on samples prepared using an *in vivo* cycloheximide treatment. We expect that SRP-RNC complexes will be highly depleted in CHX-treated cells, as existing translocation-competent RNCs will be targeted to the membrane and disengage SRP. Even membrane-targeted RNCs that have not yet disengaged SRP would be absent from the input sample as SRP-RNC complexes were purified from the soluble fraction of the cell lysate. Due to the scarcity of SRP-bound RNCs in this sample, the SRP-IP sample is likely dominated by background, i.e. RNCs that are unspecifically bound and purified during IP. Because SRP-SeRP was performed from the soluble fraction of cell lysates, the distribution of ribosomes used as input for SRP-IP was not uniform along mRNAs, with much higher read density until membrane targeting signals emerge, followed by massive depletion upon membrane association. In our experiments cycloheximide was only added post-lysis, which circumvents this problem.
2) Chartron et al. did not normalize the read distribution in the IP sample by the read distribution in the input, in their case the soluble translatome. This ratio-based data normalization is an absolutely critical step as it corrects for uneven ribosome distribution patterns in the input translatome. This analysis is particularly relevant for the approach of Chartron et al., as their total input was heavily biased towards soluble ribosomes and translating mRNA regions upstream of targeting signals. Without this normalization of the IP reads to the input, the IP sample dominated by background mimics the bias of the input translatome: a high ribosome density that drops following cotranslational targeting. In our approach, we perform the IP on total cell lysates, and perform codon-wise normalization of the IP reads over the total translatome reads.

We believe that the combination of these parameters led to the false interpretation that SRP binds already before the emergence of the first signal. When the normalization is performed as described in point 2 above, interaction peaks that are otherwise masked by background binding become detectable, as we have demonstrated in S1D.

Since we do not have an input interactome prepared using an *in vivo* cycloheximide treatment, pulling on soluble polysomes, we cannot fully recapitulate the "pre-recruitment" type of IP profiles. As an alternative, here we present the example of cytosolic RPL2A, which is not an SRP substrate, to demonstrate how non-uniform ribosome distributions in the total translatome are reflected in the IP (Fig. R12): Looking at the read distribution in the IP profile

alone suggests that SRP binds the nascent RPL2A, most prominently near codon 200. However, applying ratio-based analysis corrects for this effect and is therefore a generally accepted procedure, also used in SeRP studies from other laboratories. As omitting the input correction leads to massive biases, we prefer to not include this type of analysis in the manuscript.

Following up on the reviewer´s suggestion, we have now included a brief discussion of the differences between the approaches and their consequences in our discussion section (Page 20).

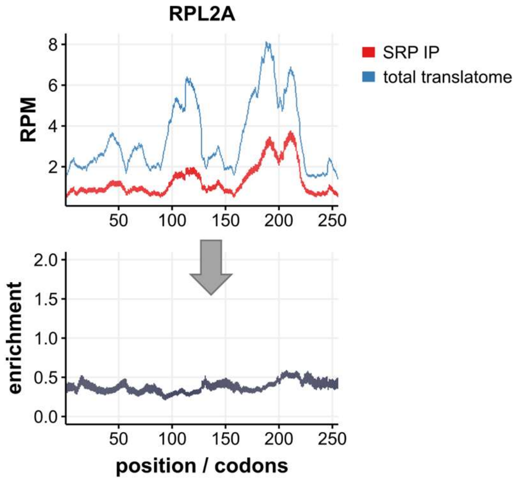

Figure R 12. Ratio based enrichment analysis (IP / total translatome) is crucial to correct for non-uniform ribosome distribution in the input translatome samples.

Reviewer #3 (Remarks to the Author):

The study from and Kotan colleagues revisits the association of the ER targeting factor SRP with nascent chains in the yeast Saccharomyces cerevisiae using the well-established selective ribosome profiling technique, in combination with membrane fractionation and SRP-depletion. The experiments confirm previous findings using complementary and related methods that the majority of yeast SRP substrates are membrane proteins and only a small subset of signal sequences engage with SRP. As has also been previously reported, translocation of signal sequence-containing also occurs co-translationally indicating this is not a unique property of the SRP-targeting pathway, but applies also to Sec71 substrates too. The study uses an almost identical approach to that previously performed by the Frydman group (Charton et al, 2016), which indicated pre-loading of SRP to mRNA prior to the onset of translation and/or synthesis of the targeting signal. The precise mechanism for this mode of binding has so far proved elusive. Here, strong association of SRP was only observed after emergence of the signal sequence/anchor consistent with the canonical mode of signal peptide recognition. Reanalysis of the Frydman data suggests the pre-associated SRP sites were of low affinity/low

confidence casting doubt on their significance. Finally, and perhaps most interestingly, the authors address largely for the first time the question as to whether multiple cycles of SRP binding and release are required for polytopic membrane protein biogenesis, and find that with these substrates ribosomes can dissociate from the translocon and re-engage SRP for targeting of later TM domains, particularly after longer cytosolic loop domains. Overall, the experiments are convincing and provide important confirmation and novel insight into protein targeting and biogenesis at the ER and will be of great interest to the field. The authors are world-leaders in the use of selective-ribosome profiling.

My only substantive criticism is the use of the SRP54 deletion strain to assess targeting in the absence of functional SRP. This strain grows very slowly following a complex adaptation process and, of particular relevance here, has reduced levels of ribosomes and reduced translation rates (Mutka et al, 2001). Analogous studies have used the inducible sec65-1 temperature sensitive mutant or inducible-degron systems to rapidly deplete SRP and thereby circumvent this adaptation. It would be important to address whether any of the effects observed here are indirect consequences of the adaptation and associated slowed translation rather than directly due to loss of SRP.

Specific points:

1) The number of signal peptides analysed seems relatively low eg Costa et al (20) report ca. 200 soluble yeast SP protein and a further ca. 130 with SP and TMD. I assume this is due to those not making the read threshold. It would be prudent to state what fraction of the different classes are being sampled here. Perhaps more importantly, is there any bias in the substrates detected? In particular, those which are short in length, which might impact their targeting pathway such as short secretory proteins.

We thank the reviewer for bringing up this potential bias. For both the calculation of SRP binding scores in Figure 1E and the analysis of co-translational targeting in Figure 1G, we apply a read coverage threshold (employing RPKMs or read density values in total translatomes, respectively). Excluding genes with limited coverage was required to robustly annotate the SRP-binding and the co-translational targeting pathway using computational tools. This type of analysis was not performed by Costa et al. In response to the comment, we now compared the distribution of lengths of transcripts that are included in targeting pathway analysis (shown in Figure 1G). As proposed by the reviewer, the analysis shows that shorter transcripts are more likely to be excluded by these thresholds. Considering this, we now indicate the fraction of ER-targeted substrate classes that we analyze. Furthermore, using a statistical model that captures the relationship between protein length and targeting mode identified by our study, we estimate the expected fraction of proteins that undergo co- versus post-translational targeting among all those annotated..

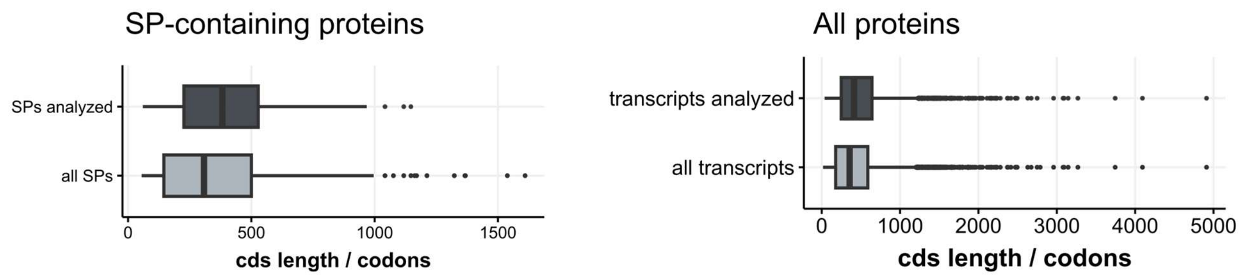

Figure R 13. Distribution of coding sequence (CDS) lengths of annotated versus analyzed yeast transcripts. The CDS lengths of signal peptide-containing transcripts that passed the read coverage threshold used for targeting pathway analysis (Fig. 1G) were compared with all annotated signal peptide-containing proteins. The same comparison was additionally performed for the full set of proteins, applying the identical read coverage threshold.

2) A technicality, but Δsrp54 is not a complete deletion of SRP as Δsrp might infer, all the other components are still present, just the functionally essential SRP54 is missing. This is unlike other SRP gene deletions where the particle is more fundamentally destabilised, it would be helpful to clarify this. GFP tagging of SRP54, doesn't appear to fully complement with either N or C-trm fusion, are the differences in doubling times between WT, N and C-tagged and deletion statistically significant? Was there a reason for not tagging Srp72 which does fully complement (Mason et al, 2000) and results would also have been directly comparable with Chartron et al (2016)?

We have revised the text to more clearly state that we are not using a strain that lacks all SRP components. In response to the reviewer's concern regarding GFP-tagging of SRP54, we have additionally performed SeRP experiments using a strain encoding an N-terminally GFP-tagged Sec65 (as suggested by Reviewer 2). This strain did not display any detectable growth defect (Figure R4), and SeRP experiments performed in this background revealed an SRP substrate distribution across the different protein classes that is consistent with our original findings (Figure R3).

3) As mentioned above, assessing SRP-dependence might be better addressed in a sec65-1 strain, or auxin-degron system allowing rapid inducible inhibition of the SRP pathway without the adaptation that occurs in the null. To circumvent repeating all the experiments with rapid depletion, can one or other approaches be used to confirm SRP-dependence (eg in direct translocation assays) for some of the novel substrates identified by profiling in the null strain?

We agree with the reviewer that distinguishing SRP-independent substrates from proteins whose targeting behavior changes only after adaptation to chronic SRP loss is important. Our approach of combining SRP-SeRP profiling in wild-type cells with targeting analysis in the Δ*srp54* strain has allowed us to resolve these classes.

Importantly, the proteins we classify as "SRP-independent" are not bound by SRP in wild type cells yet are cotranslationally targeted to the ER membrane both in wild type and in the Δsrp54 strain. These observations strongly suggest that their cotranslational targeting is independent of SRP also in wild-type cells, rather than arising from the adaptation.

In contrast, the “SRP-facultative” class comprises bona fide SRP substrates in WT cells that nevertheless are cotranslationally targeted in the deletion strain. It is plausible that these proteins only start using alternative targeting pathways as a result of the adaptation to the SRP loss. This interpretation is consistent with the behavior of SRP-facultative Kar2 and Dap2, previously analysed by Mutka et al. (2001), which show progressively increased ER targeting hours after SRP depletion. Our results further show that the adaptation process only confers cotranslational targeting to a limited subset of proteins. Most TMD-containing proteins do not switch to cotranslational targeting in the Δ*srp54* strain, and most cotranslationally targeted SP-containing proteins are not SRP substrates in the first place. We have now expanded the discussion to emphasize these points more clearly.

4) The window size of 9 in the Arst et al study was only chosen over 11 or 19 based on the then available structure of bacterial Ffh. Earlier studies such as Ng et al (1996) had used the larger Kyte-Doolittle window size of 11, is this any better match for predicting SRP-dependency? It would also be helpful to assess how the FILVW-PGS scores matches to SRP-dependency determined in the Costa at al study upon rapid depletion of SRP.

We have compared how well our experimental data match (i) the maximum Kyte-Doolittle hydrophobicity using a window size of 11, (ii) the compound score calculated using a window size 11 (See figure below, A-B), and (iii) maximum Kyte-Doolittle hydrophobicity or compound score using a window size of 9 (Fig. S3F and H). To quantitatively compare the different methods, we performed a ROC (Receiver Operating Characteristic) analysis. Among all metrics tested, the FILVW–PGS score (hp_count–SPG) provided the highest accuracy for predicting SRP engagement. The use of a window size of 11 did not improve predictive performance relative to a window size of 9 for either maximum hydrophobicity or the compound score.

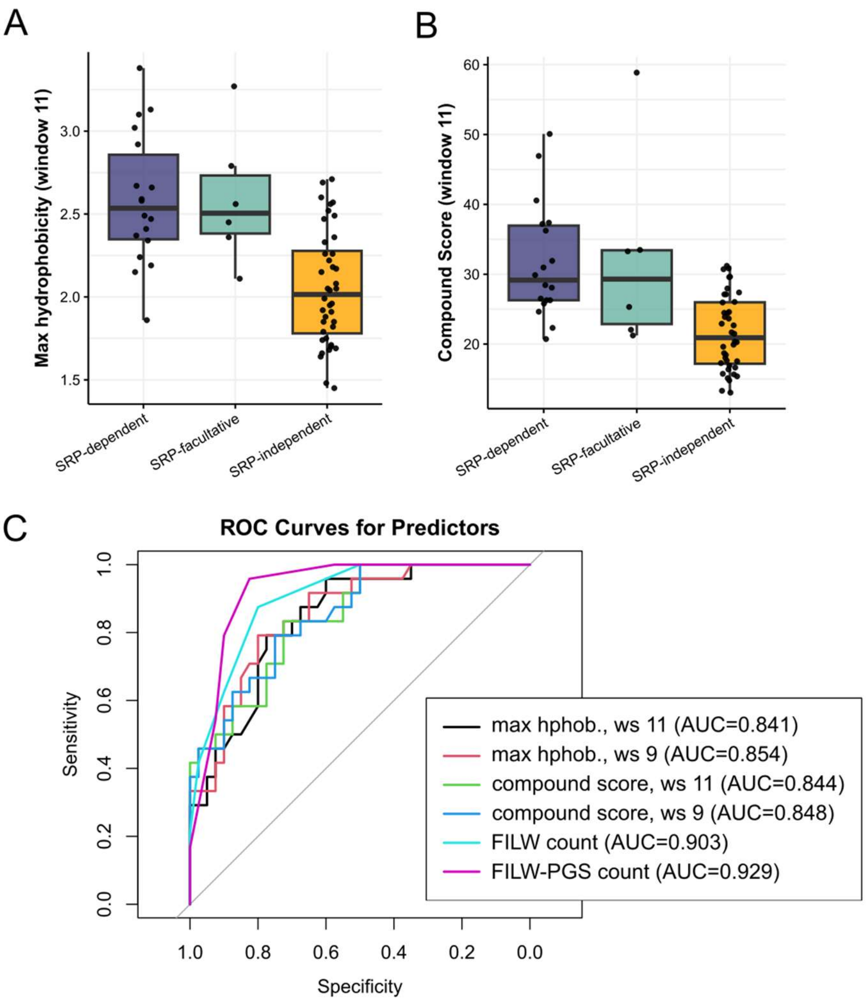

Figure R 14. Comparison of signal peptide sequence features with experimental SRP binding and dependency data. A) Maximum Kyte-Doolittle hydrophobicity of SPs calculated using a sliding window of 11 amino acids (Ast et al., 2013) in groups of proteins binned according to their SRP-pathway usage. B) Compound hydrophobicity scores of SPs (window size: 11) compared to their SRP-pathway usage, as calculated by Ast et al., 2013. C) Receiver operating characteristic (ROC) analysis comparing the predictive performance of each metric based on how well they classify experimentally determined SRP engagement across varying thresholds.

As suggested, we now also assessed how both our experimental classification and the FILVW–PGS score relate to SRP-dependency determined in the Costa et al. study. Of note, Costa et al. did not annotate SRP-dependent versus SRP-independent substrates based solely on their experimental data, but instead benchmarked their results against predictions from Ast et al.

While Ast et al. classify the majority of signal peptide-containing proteins as SRP-independent, many of these proteins clearly exhibit reduced ER proximity upon SRP depletion in the Costa et al. study (Fig. R15A,C). In contrast, our experimentally defined classes determined in the Δsrp54 strain agree very well with the Costa et al. data determined upon rapid SRP depletion: proteins classified as SRP-dependent show a clear loss of membrane proximity, whereas SRP-independent proteins are largely unaffected (Fig. R15B,D).

While the Costa et al. data measure defects in cotranslational targeting upon SRP depletion, the FILVW–PGS score instead predicts the likelihood that a targeting signal is bound by SRP, rather than whether targeting is strictly SRP-dependent. Because some proteins engage SRP in addition to alternative cotranslational targeting pathways (i.e. SRP-facultative substrates), it is expected that a subset of proteins with high FILVW–PGS scores will not display severe targeting defects upon SRP depletion. In contrast, proteins falling below the FILVW–PGS threshold, which are predicted to not bind SRP, would be expected to be largely unaffected by loss of SRP. Consistent with this, these proteins show little to no loss of ER proximity upon SRP depletion in the Costa et al. dataset (Fig. R15E).

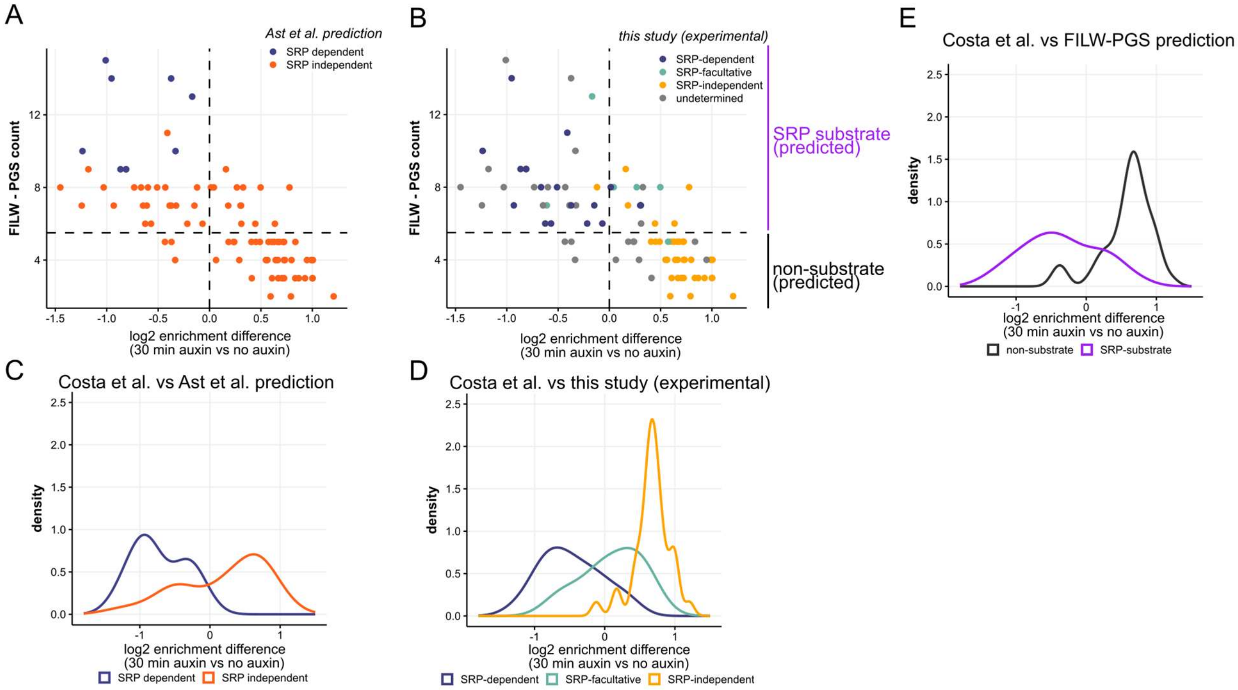

Figure R 15. Comparison of our findings with data from Costa et al. Data from Costa et al. were reanalyzed to calculate membrane-enrichment differences of signal peptide–containing transcripts with or without 30 min of auxin-mediated SRP depletion. A) Membrane-enrichment differences plotted against FILW-PGS scores, with SRP dependence predictions according to Ast et al. indicated. B) Membrane-enrichment differences plotted against FILW-PGS scores, with experimentally determined SRP pathway usage according to our study indicated. C–D) Density plots corresponding to panels A and B, respectively. E) Density plot showing membrane-enrichment differences for proteins predicted to be SRP substrates or non-substrates based on FILW-PGS scores.

5) The legend of figure 2 appears truncated.

We apologize for the mistake. The legend is now fixed in the revised version of the manuscript.

6) As already mentioned above, the Δsrp54 strain has lower rates of translation due to reduced levels of ribosomes, could this not contribute to the reduced ribosome collisions observed? Could the impact of reduced translation on frequency of collisions be in part addressed by looking at the profile data for known SRP-independent ribosome collision events in the dataset?

We agree with the reviewer´s concern that reduced translation rates and ribosome abundance in the Δsrp54 strain could lead to fewer ribosome collisions. To assess whether collisions are globally reduced, we therefore analyzed the prevalence of SRP-independent collision events in wild-type and Δsrp54 cells. Specifically, we performed metagene analysis of collided disomes on 340 cytonuclear transcripts containing 470 polyproline stretches (≥3 prolines), which are established SRP-independent ribosome stalling sites. Normalized reads from collided disomes were aligned at the position where the first proline codon is at the ribosomal P-site, revealing an accumulation of these reads at this position both in wild-type and Δsrp54 cells (Fig. R16). Notably, polyproline-induced collisions were not reduced in the Δ*srp54* strain. This argues against a global decrease in collision frequency and supports a specific defect in SRP-dependent cotranslational membrane targeting. We have now included this result in the revised manuscript (Fig. S3C).

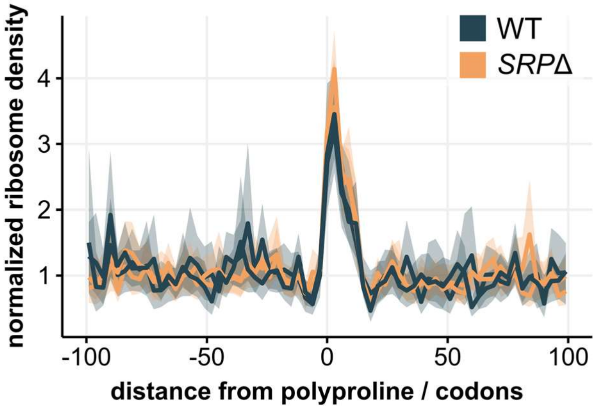

Figure R 16. Metagene analysis depicting distribution of collided disomes along transcripts encoding polyproline stretches (≥3 prolines), aligned at the start of the polyproline stretch.

7) Ribosome profiling of the ribosome collision marker protein Hel1 (Matsuo and Inada, 2021) also indicated enrichment on secretome mRNAs before onset of SRP recognition, consistent with the findings here. However, it is interesting in this study loss of ribosome binding increased Hel1 association. How would these observations be rationalised eg by different pools of stalled ribosomes? The authors might wish to comment on these observations.

While both studies (Matsuo et al. and our present study) analyze collided ribosomes, we believe the results are not directly comparable due to substantial differences in experimental design and in the ribosome populations captured. Matsuo et al. employ a mild RNase digestion followed by Hel2 immunoprecipitation and a subsequent harsher digestion intended to convert all collided, Hel2 interacting species into monosomes, followed by sequencing of 30 nucleotide footprints. In this study, neither the input nor the Hel2-associated footprints accumulate at positions corresponding to the emergence of ER-targeting signals, but instead are enriched at very early stages of translation, prior to targeting signal exposure. We therefore speculate that the Matsuo et al. approach preferentially captures a population of more persistent or severe ribosome

collisions that engage the ribosome quality control pathway, consistent with the presence of trisomes and higher-order collided species in the Hel2-immunoprecipitated material. Hel2 binding to this quality control-associated collision pool may increase upon SRP depletion.

In contrast, our analysis focuses on disomes (protecting 60 nt footprints) that arise transiently during cotranslational targeting, likely as a consequence of SRP-mediated pausing. These collisions occur at defined positions coincident with targeting signal emergence and may resolve rapidly when targeting has been completed, without recruiting Hel2. Thus, SRP depletion would be expected to reduce this targeting-associated collision population, while potentially increasing Hel2 engagement with a distinct class of aberrant stalled ribosomes.

8) In figure S3 can the %age of targeting that is co-translational be shown in WT (SRP54) for comparison, or is this only those substrates that are co-translational in WT (i.e. WT is 100%), if the latter, can that be made clearer?

In the revised manuscript, we now include a plot (Fig S5A) showing the number of proteins that are co-translationally targeted in the *srp54*Δ strain, out of those that are cotranslationally targeted in WT yeast.

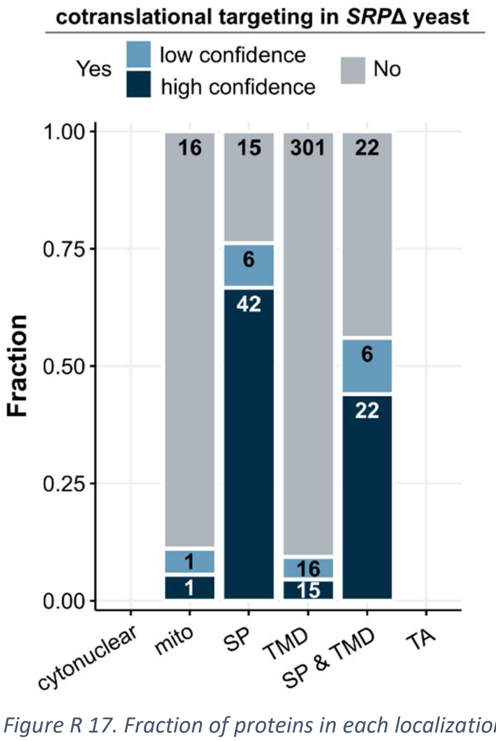

Figure R 17. Fraction of proteins in each localization category that is cotranslationally targeted in Δsrp54 yeast, out of those that are cotranslationally targeted in wild-type yeast.

9) Figures S3C and D are lacking the schematic showing position of TMD/SP

We have added the TMD and SP positions to the figures in the revised version of the manuscript.

10) In figure S3M can traces be shown for previously identified Snd TM substrates (eg Ynl181w) in the WT v Δsnd1 strain as was shown for Gas1.

We have included three example traces for membrane proteins reported to be SND substrates by Aviram et al., 2016 in the revised manuscript (Fig. S6K). Although these genes are poorly expressed and read coverage is limited, we can clearly distinguish between co- and posttranslational targeting routes: For Ynl181w (Pbr1), soluble-RP does not indicate a cotranslational targeting in wild-type or mutant yeast strains, as the soluble/total ratio remains centered around 1 throughout translation. Tpo4 and Gsc2 (reported to specifically lose ER-proximity in the Δsnd1 strain) are cotranslationally targeted, however, no targeting defects are observed in the absence of Snd1.

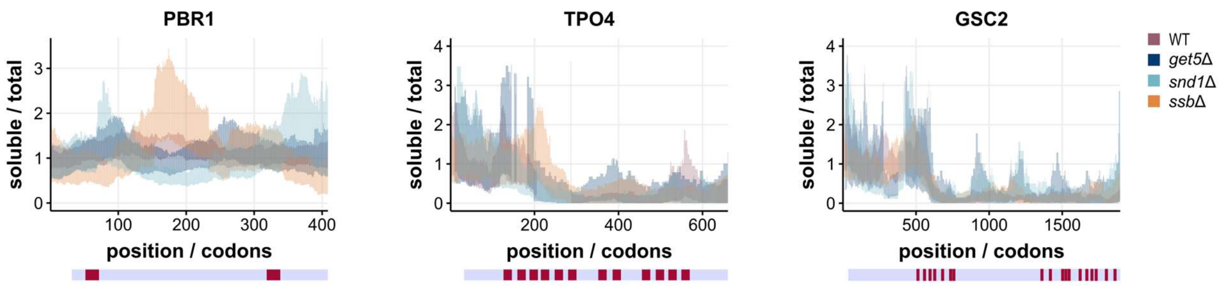

Figure R 18. Single gene targeting profiles based on soluble-RP performed in the indicated strains.

11) It was perhaps slightly surprising that luminal loops also led to ribosome dissociation from the translocon (Fig. 4), is this still dependent upon the presence of the preceding TM? ie is this effect seen with soluble secretory proteins once the signal sequence is cleaved or where Nexo membrane proteins are initially targeted by a Sec71-dependent signal sequence? The SRP binding profile for Erj5 (Fig. 1H) suggests that is possibly the case for the latter.

We agree, the observation that luminal loops can coincide with ribosome dissociation from the translocon was initially surprising to us as well. Ribosome dissociation is also observed after ER targeting mediated by signal peptides, for soluble secretory proteins (Fig. S5L, Fig R19A-B), as well as for some type I membrane proteins containing an N-terminal signal peptide followed by a transmembrane domain (e.g. Pun1; Fig. 4A, Fig. R19C). In the case of Erj5 however, the SP emergence does not mediate efficient cotranslational targeting (Fig. 1H), which instead occurs upon emergence of the TMD encoded near codon 180, followed by ribosome dissociation. Unfortunately, the coverage of this gene in the soluble-RP experiment with PK treatment is not sufficient to assess the PK-sensitivity prior to the TMD-mediated targeting (Fig. R19D).

These findings raise the question of how back-sliding of nascent chain segments with luminal localization is prevented during ribosome dissociation. We consider that several factors may contribute to this. Dependent on the specific features of the luminal segment it could be retained in the lumen by (i) the uncleaved signal peptide, (ii) folding, or (iii) binding of ER luminal factors, for example chaperones. We have revised our discussion, mentioning the potential relevance of

folding of luminal domains, in addition to binding binding of ER luminal factors previously discussed (Page 22).

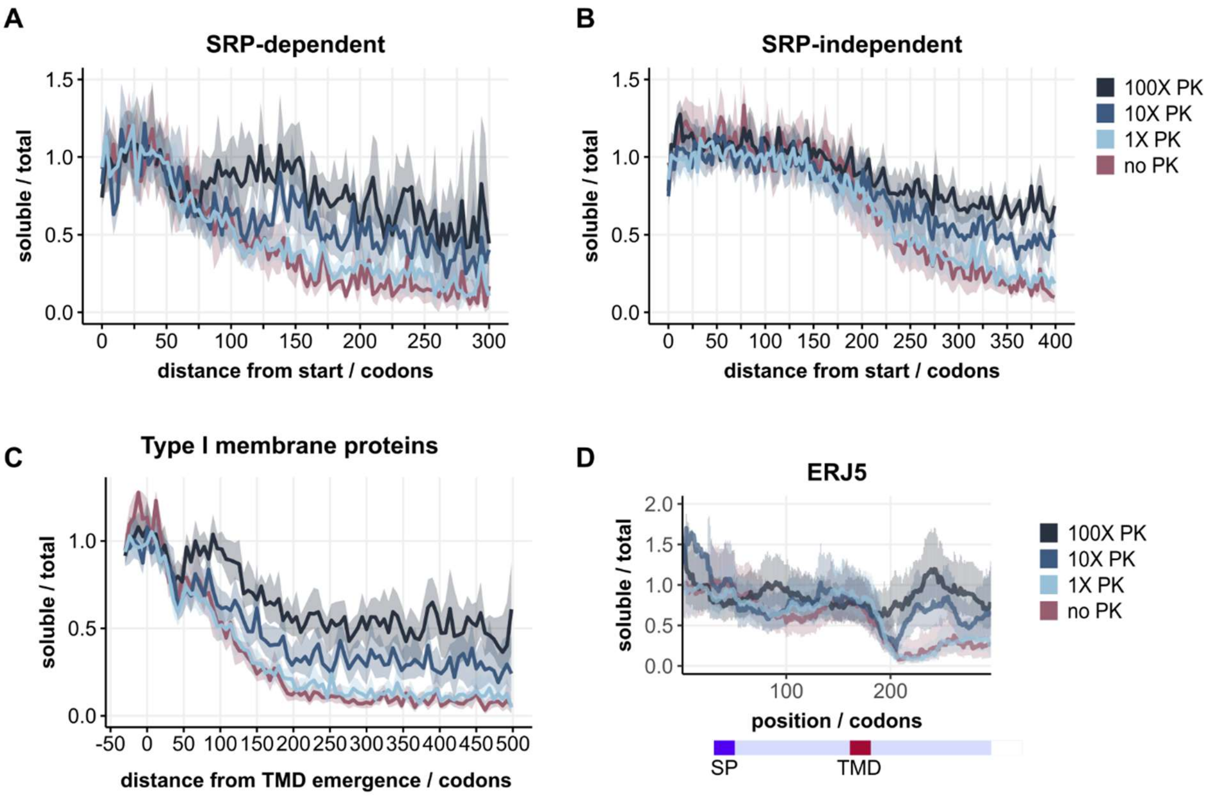

Figure R 19. Ribosome dissociation during synthesis of soluble segments of membrane proteins. A-B) Metagene analysis depicting PK-sensitivity of membrane association for soluble secretory proteins that are SRP-dependent or –independent, as categorized in this study. C) Metagene analysis depicting PK-sensitivity of membrane association for Type I membrane proteins. D) Single gene profile of ERJ5, showing PK-sensitivity of its membrane association.

12) There is a correlation between Ssb binding and ribosome dissociation for the examplars shown in the figure 4, can this also by shown in a meta-plot with Ssb binding against loop length.

The onset of Ssb binding occurs at variable periods relative to loop emergence across different proteins, which makes a metagene analysis poorly suited to capture this relationship. Averaging Ssb binding signals aligned to loop length would obscure protein-specific differences in binding onset and duration. To address the reviewer's question, we instead performed an Ssb peak-detection analysis to compare the number of Ssb binding periods across proteins with long cytosolic loops, long luminal loops, and short loops. To specifically exclude interactions that may occur prior to membrane targeting, we limited the analysis to Ssb binding periods occurring after the onset of cotranslational targeting for each class. This analysis revealed the highest number of post-targeting Ssb interactions with proteins harboring long cytosolic loops, supporting the generality of our findings that ribosome undocking allows chaperones such as Ssb to bind these segments. These results are now presented in Figure 4G and Figure R9, above.

13) In figure 5, there is a mix of mammalian and yeast terms for chaperones eg BiP and Ssb. Would be better to be consistent eg using Kar2 instead of BiP.

We agree with the reviewer and have adjusted Figure 5 accordingly.

REVIEWERS' COMMENTS

Reviewer #1 (Remarks to the Author):

The authors have addressed my concerns, and I have no further comments.

Reviewer #2 (Remarks to the Author):

The authors have provided substantial additional work, analysis, and explanations in response to the referee comments, and this effort is greatly appreciated. The proteome-wide analysis is valuable for the field and will stimulate new directions of research. I am supportive of publication and do not need to review this study again. I would suggest the following minor text revisions before publication.

1) The further profiling analysis without detergent and using a tagged strain that has a normal growth phenotype is helpful and reassuring. Notably however, the difference in the SRP binding score between the two conditions for TMD containing proteins is pretty striking (mean around 10 for the sample with detergent and using the GFP-SRP54 strain, but around 40 for the sample without detergent using the GFP-Sec65 strain). This seems to be mostly down to detergent. The authors should comment about this in the paper because the difference is non-trivial (i.e., similar to the 4-fold difference seen between SP and TMD).

We agree with the reviewer that SRP enrichment is consistently higher in detergent-free SRP-SeRP experiments, suggesting that omitting detergents may enhance the capture of SRP-ribosome interactions. At the same time, detergent-free conditions have drawbacks for selective ribosome profiling, such as reduced resolution. We have now explicitly addressed this point in the revised manuscript.

Importantly, we note that factor-binding enrichment values derived from selective ribosome profiling experiments performed at different times are inherently subject to variability. Such differences likely reflect minor variations in the immunoprecipitation-based purification of factor-engaged ribosomes. Accordingly, absolute enrichment values should be interpreted with caution when comparing datasets that were not generated in parallel. In contrast, the enrichment differences observed between signal peptides and transmembrane domains originate from the same dataset and are therefore directly comparable.

2) In discussing SRP-mediated arrest, it is probably worth noting that the strong arrest originally described (PMID 7309797) was an artefact due to the mixing of plant translation extracts with mammalian SRP. In homologous systems, no such arrest was seen (see PMID 2415357, 3027096, 3087745). Even in the mixed system, arrest was not seen for other substrates (PMID 3027096). Only when excess SRP is added is a modest slowdown seen (PMID 2556403). The structural explanation for the lack of arrest is that the Alu domain can simply move out of the way when elongation factors bind, allowing simultaneous binding of SRP and elongation factors (see Fig. 7 of PMID 26158507).

We have now noted that the strong arrest observed in heterologous systems is likely an artefact of these experimental setups. Vorhees et al., 2015 (PMID 26158507) specifically discuss that although elongation factors can still access the GTPase center when SRP is bound to RNCs via its S domain, the high local concentration due to S domain tethering, allows it to compete with eEF1 and eEF2. They propose that this is the likely cause of translation slow down by SRP. This model is in good agreement with our observations, which likewise support a moderate translation slowdown rather than a pronounced arrest.

3) The authors cite PMID 8626442 as prior evidence that SRP mediates insertion of an internal TMD when the loop preceding it is long. I would suggest that this be removed because the primary purpose of that paper is to investigate features that promote re-initiation, with the role of SRP being a minor part. But more importantly, inspection of the data in that paper does not support the conclusion about SRP. The key data is in Fig. 4B of that study. When the loop is extended from 58 to 100, there is a very small increase in re-initiation, but the error bars are huge and this cannot be considered robust or statistically significant. More notably however, when the loop is further extended to 200, the effect is completely lost. So I would just omit lines 465-467.

We agree that the data in the cited study contain large error bars and that the effect is lost upon extension to 200 residues. In light of this, we have softened our interpretation in the revised manuscript to avoid overstating the strength of the evidence.

At the same time, we note that the reported increase in re-initiation efficiency with increasing SRP concentrations follows a consistent directional trend, which suggests that the effect may be robust. We further note that overall re-initiation efficiencies at the loop length of 200 residues are very low, raising the possibility that additional factors become limiting under these conditions and may mask any contribution of SRP.

While we acknowledge the limitations of this study, we believe it remains informative in the context of our findings and therefore retain a more cautious reference to it in the manuscript.

Reviewer #3 (Remarks to the Author):

The reviewers have now satisfactorily addressed the concerns I had raised adding addition data and correcting the minor errors.
